# LncRNA-BC069792 suppresses tumor progression by targeting KCNQ4 in breast cancer

**DOI:** 10.1186/s12943-023-01747-5

**Published:** 2023-03-01

**Authors:** Yunxiang Zhang, Xiaotong Dong, Xiangyu Guo, Chunsen Li, Yanping Fan, Pengju Liu, Dawei Yuan, Xialin Ma, Jingru Wang, Jie Zheng, Hongli Li, Peng Gao

**Affiliations:** 1 Department of Pathology, The First Clinical Medical College of Weifang Medical University, Weifang people’s Hospital, Weifang, 261100 China; 2 Key Laboratory for Experimental Teratology of the Ministry of Education and Department of Pathology, School of Basic Medical Sciences, Shandong University, Jinan, 250000 China; 3 Department of Pathology, Qilu Hospital, Shandong University, Jinan, 250012 China; 4 College of Pharmacy, Qingdao University, Qingdao, 266071 China; 5 Department of Economics, Qingdao University, Qingdao, 266061 China; 6 Qingdao Geneis Institute of Big Data Mining and Precision Medicine, Qingdao, 266000 China; 7 Department of Diagnostic Pathology, School of Basic Medical Sciences, Weifang Medical University, Weifang, 261053 China

**Keywords:** IncRNA BC069792, Breast cancer, Proliferation, Invasion and metastasis, KCNQ4

## Abstract

**Background:**

Breast cancer is the most common malignant tumor that threatens women's health. Attention has been paid on the study of long- non-coding RNA (lncRNA) in breast cancer. However, the specific mechanism remains not clear.

**Methods:**

In this study, we explored the role of lncRNA BC069792 in breast cancer. In vitro and in vivo functional experiments were carried out in cell culture and mouse models. High-throughput next-generation sequencing technology and real-time fluorescence quantitative PCR technology were used to evaluate differentially expressed genes and mRNA expression, Western blot and immunohistochemical staining were used to detect protein expression. RNA immunoprecipitation assay and dual-luciferase activity assay were used to evaluate the competing endogenous RNAs (ceRNA), and rescue and mutation experiments were used for verification.

**Results:**

We found that lncRNA BC069792 was expressed at a low level in breast cancer tissues, and significantly decreased in breast cancer with high pathological grade, lymph node metastasis and high Ki-67 index groups. Moreover, BC069792 inhibited the proliferation, invasion and metastasis of breast cancer cells in vitro and in vivo. Mechanically, BC069792 acts as a molecular sponge to adsorb hsa-miR-658 and hsa-miR-4739, to up-regulate the protein expression of Potassium Voltage-Gated Channel Q4 (KCNQ4), inhibits the activities of JAK2 and p-AKT, and plays a role in inhibiting breast cancer growth.

**Conclusions:**

LncRNA BC069792 plays the role of tumor suppressor gene in breast cancer and is a new diagnostic index and therapeutic target in breast cancer.

**Supplementary Information:**

The online version contains supplementary material available at 10.1186/s12943-023-01747-5.

## Background

Breast cancer is the most common malignancy that threatens women's health worldwide. The morbidity and mortality of breast cancer is increasing. In 2020, the global incidence of breast cancer ranked the first among new malignant tumors in women, accounting for 30% of the incidence of all malignant tumors [1–3]. According to the statistics released by the National Cancer Center of China, there were around 420,000 new cases of breast cancer among Chinese women in 2020. The most effective way to treat cancer is early detection and precision treatment, which depends on understanding of the pathogenesis of breast cancer. The carcinogenic mechanism of breast cancer is complex, involving endocrine hormones, various genes, epigenetic alterations, and so on [4]. Therefore, finding new molecular markers and therapeutic targets is critical for extending survival time and improving the quality of life of patients.

By interacting with DNA, RNA and proteins, lncRNAs regulate chromatin structure and function, modulate the transcription of neighboring and distant genes, and affect RNA splicing, stability and translation [5]. Accumulating evidence suggests that lncRNAs play complex and precise regulatory roles in cancer initiation and progression by acting as oncogenes or tumor suppressors [6, 7]. At present, the role of lncRNAs in liver cancer, breast cancer and bladder cancer has been reported [8–10]. The effect on breast cancer is the focus of current research. Our studies had found that LncRNA-BM466146 and KRT19P3 play important regulatory roles in the occurrence, development and metastasis of breast cancer [11, 12]. LncRNA BC069792 is a 515-nt RNA from the lncRNA gene chip discovered by our research group in our previous study (GEO: GSE72307). This study investigates the function and mechanism of BC069792 in breast cancer, clarifies it as a key regulatory molecule involved in breast cancer proliferation, invasion and metastasis. This study provides a new target for molecular targeted therapy of breast cancer.

## Methods

### Clinical breast cancer specimen collection and culture of breast cancer cells

A total of 102 pairs of fresh breast cancer specimens and adjacent normal breast tissues were collected from inpatients in Weifang People's Hospital from February 2019 to June 2019. The relevant clinicopathological data, such as age, pathological stage, tumor size, Ki-67 index, lymphatic metastasis, calcification, necrosis were included. Four pairs of specimens with tumor cell content < 50% were excluded. This study was approved by the Ethics Committee of Shandong University. Human breast cancer MDA-MB-231 and MDA-MB-468 cell lines were purchased from ATCC and cultured in L15 medium (Invitrogen, Carlsbad, California, USA) at 37 °C in a 5% CO_2_ cell incubator.

### RNA extraction and content determination

RNA from tissues was extracted using Axygen total RNA miniprep kit (Corning Life Sciences Co., Ltd., Jiangsu, China), and RNA from cells was extracted using TRIzol reagents (Invitrogen, Carlsbad, California, USA). The extracted RNA was reversely transcribed using a reverse transcription kit (TOYOBO, Shanghai, China) and the content of BC069792 was detected using KAPA SYBR FAST qPCR Kits (KAPA, Wilmington, Massachusetts, USA). GAPDH was selected as the internal reference, and the primer sequences were shown in Table 1. miRNAs in paraffin tissues were extracted using the paraffin-embedded tissue miRNA rapid extraction kit (spin column type, Biotech, Beijing, China). Quantitative detection of miRNA was performed on the extracted miRNA or total RNA using the All-in-One™ miRNA qRT-PCR Detection System 2.0 User Manual (GeneCopoeia, Rockville, MD, USA).

**Table 1:** The relationship between the expression of BC069792 in breast cancer and the clinicopathological parameters of patients

Clinical parameter	Quantity	BC069792		Chi-square test	
		LOW expression	High expression	*x2*	*P, price*
age				1.951	0.162
< 65	78	59	19		
≥ 65	20	12	8		
pathological grading				8.120	0.004
I	18	7	11		
II + III	80	59	21		
tumor size				1.395	0.238
< 2 cm	26	3	23		
≥ 2 cm	72	16	56		
Hemorrhage, calcification, necrosis, and cyst change				3.623	0.057
NO	43	11	32		
YES	55	6	49		
lymphatic metastasis^a^				10.324	0.001
NO	54	18	36		
YES	37	25	12		
Out-67 index					0.045
< 30%	36	0	36		
≥ 30%	62	7	55		

^a^Seven cases were not tested for lymph node metastasis. Inspection level *a* = 0.05

### The location of BC069792

Cytoplasmic & Nuclear RNA Purification Kit (Wuhan Aimeijie Technology Co., Ltd., Wuhan, China) was used to extract RNA from the cytoplasm and nucleus, respectively, and the content of BC069792 was detected by teal-time fluorescence quantitative polymerase chain reaction (RT-qPCR) with GAPDH as the cytoplasmic reference and U6 as the nuclear reference.

Ribonucleic acid fluorescence in situ hybridization kit (Guangzhou, RiboBio, China) was used to detect FISH. Cy3-labeled probe BC069792 or control probe for U6 single-stranded RNA and 18 s rRNA. A fluorescence microscope (Olympus, Tokyo, Japan) was used to observe RNA localization and take photos.

### Overexpress or knock down BC069792

Lipofectamine 2000 transfection reagents (Invitrogen,Carlsbad,California, USA) were used to transfect overexpression plasmids and small interference RNA (siRNA) in breast cancer cell lines MDA-MB-231 and MDA-MB-468. The transfection efficiency was detected 24 h after transfection.

### Cell growth and proliferation assay

After 24 h of transfection, 3000–5000 cells per well were seeded into 96-well plates in quadruplicate and incubated at 24 h, 48 h, 72 h, and 96 h. 10 μl CCK-8 reagent (CCK-8 cell proliferation-cytotoxicity detection kit, Bebo, Shanghai, China) was added and cells were incubated for 2–3 h. After that, light absorbance at 450 nm was detected. Transfected Cells were cultured in 24-well plates for 24 h. Fluorescence staining with an EdU assay kit (Biyun, Shanghai, China) was performed. Cells were photographed under a fluorescence microscope and cell proliferation rate was calculated.

### Cell migration and invasion assay

Twenty-four hours after transfection, the cells were resuspended in serum-free medium and placed in a Transwell chamber (Corning, Cambridge, MA, USA) (3 × 10^4^/300μl). Then, 600 μl of medium containing 10% fetal bovine serum was added to the outer side of the chamber. The chamber was collected after incubating for 24 h. The number of cells passing through the chamber was counted after Giemsa staining. For invasion experiments, Transwell chambers were pre-coated with Matrigel matrix (BD Biosciences, San Jose, CA, USA).

In the wound healing assay, the transfected cells were spread into a 6-well plate, and grown to 80% -90% confluence. A scratch was created in the cell monolayer. The width of the scratch area was photographed at 0 h, 12 h, 24 h and 48 h. The migration rate at each time point was calculated and the curve was drawn.

### Stability test of BC069792

Cells with log-growth period were seeded into 24-well plates. The actinomycin D (5 mg/ml) [13] was added after cell starvation for 24 h. Cells were harvested at 0 h, 0.5 h, 1 h, 4 h, 8 h and 12 h in the 37℃ 5% CO_2_ cell incubator and RNA was extracted. Then lncRNA stability was determined by RT-qPCR assay.

### In vivo tumorigenesis and metastasis experiments

Lentivirus LV-BC069792 and LV-NC (9 × 10^8^TU/ml, Jima gene, Shanghai, China) were used to infect breast cancer cell line MDA-MB-231 to construct stable overexpression BC069792 cell lines and control cells. 4-week-old female BALB/c nude mice (purchased from Beijing Weitong Lihua) were subcutaneously injected to construct subcutaneous tumor model and distant metastasis model was constructed by tail vein injection in nude mice [14]. All nude mice were kept in the SPF animal room at Weifang Medical University Animal Center. All animal experiments were operated in accordance with the "Laboratory Animal Management Treaty" and were approved by the Experimental Animal Ethics Committee of Weifang Medical University. After 5 weeks, the nude mice in the subcutaneous tumor-forming group were imaged in vivo, and the tumors were removed and the size was measured (V = 1/2 × the length of the tumor × width^2^). Eight weeks later, the nude mice in the distant metastasis group were sacrificed by cervical dislocation after in vivo imaging. The lung, liver and other tissues were taken out to observe metastatic lesions, photographed and recorded. All the preserved tumors and tissues were divided into two parts, one was used for HE staining to observe tumor formation and metastasis; the other was fresh tissue for future RNA or protein extraction.

### Transcriptome sequencing to detect the changes of downstream genes in BC069792

MDA-MB-231 cells in logarithmic growth phase were inoculated into 6-well plates. After 24 h of incubation in the incubator, pcDNA3.1-BC069792/pcDNA3.1 and si-BC069792/si-NC were transfected. Three replicate wells were set in each group, RNA was extracted 24 h after transfection, and the concentration and purity of RNA were detected. The transfection efficiency was detected by RT-qPCR experiment. RNA-seq was performed by the Beijing Yuancode Biotechnology Co., Ltd., using the BGI sequencer mgiseq2000 instrument.

### Detection of protein content

MDA-MB-231 and MDA-MB-468 cells were lysed and proteins were extracted using high-efficiency RIPA tissue/cell lysis buffer (Solarbio, Beijing, China). Antibodies used were as follows: KCNQ4 antibody (1:1000, Abways, Shanghai, China), PI3K antibody (1:1000, Abways, Shanghai, China), AKT1/2/3 antibody (1:1000, Abways, Shanghai, China), p-AKT antibody (1:1000, Abways, Shanghai, China) and GAPDH antibody (1:5000, CUSABIO, Wuhan, China). Exposure was performed using a chemiluminescence gel imaging system.

### RNA Immunoprecipitation (RIP)

According to the manufacturer's instructions, RIP experiments were carried out using RIP kit (Merck, Shanghai, China) to lyse cells and prepare immunoprecipitation magnetic beads to construct immunoprecipitation of RNA binding protein-RNA complex. According to the instructions, immunoprecipitation RNA was purified by RNA, and the recovered RNA was quantitatively analyzed by RT-qPCR.

### Dual-luciferase assay

MiRNA mimics and GLO plasmids were co-transfected with Lipo2000 in breast cancer cells MDA-MB-231 and MDA-MB-468 for 48 h, and then the relative activity of luciferase was calculated by DL101-Dual Luciferase Reporter Assay Kit (Nanjing, China).

### Statistical analysis

SPSS Statistics 26 software and GraphPad Prism 7 software were used for statistical analysis of the data. The Mann–Whitney U rank sum test was used to count the difference in the expression of BC069792 in normal tissues and breast cancer tissues. The Receiver Operating Characteristic (ROC) curve was used to evaluate the diagnostic value of BC069792 in breast cancer, and the Youden index was used to determine the cut off value of BC069792. The relationship between the expression of BC069792 and clinicopathological parameters was analyzed by chi-square test and Fisher's exact test. Correlation analysis was performed using Spearman's correlation test. Student's t-test and one-way ANOVA were used to analyze the differences of two or more groups. A *P* value < 0.05 was considered statistically significant.

## Results

### The expression of BC069792 in breast cancer tissues

**Fig. 1:**
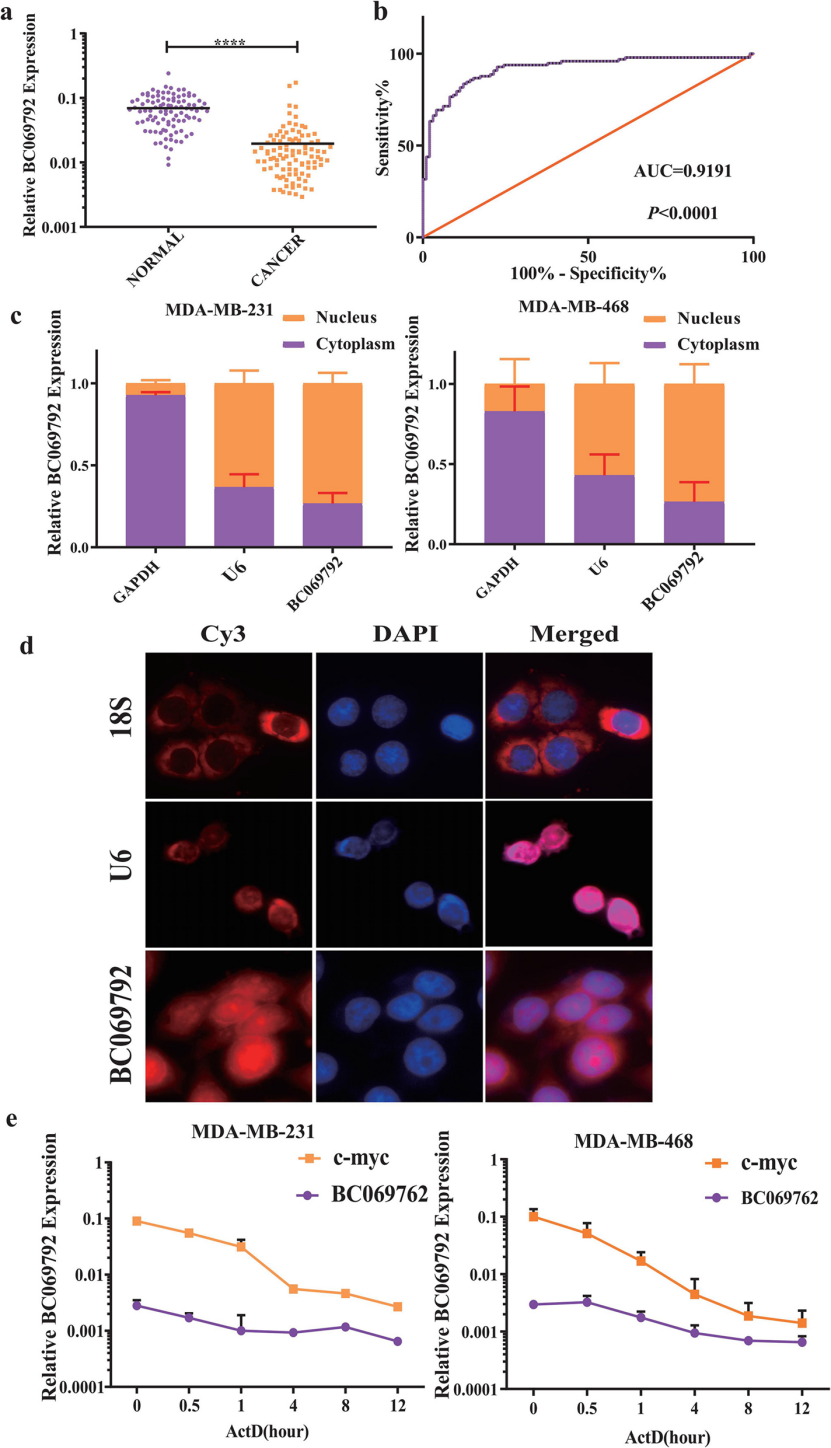
BC069792 expression in breast cancer tissue. **a** The expression of LncRNA BC069792 in breast cancer tissue was significantly lower than that in adjacent normal breast tissue, the difference was statistically significant, *P* < 0.001. **b** The results of ROC curve analysis showed that BC069792 distinguished breast cancer tissue from normal breast tissue, the area under the curve was 0.9191, and the* P* value was < 0.0001. **c** In the MDA-MB-231 cell line, about 27.6% of BC069792 was expressed in the cytoplasm and 72.4% in the nucleus. In the MDA-MB-468 cell line, about 26.4% of BC069792 was expressed in the cytoplasm and 73.6% in the nucleus. **d** The results of RNA FISH assay showed that BC069792 was distributed in both nucleus and cytoplasm in MDA-MB-468 cell line. **e** Stability assay of BC069792 in MDA-MB-231 and MDA-MB-468 (*P* < 0.05, *P* < 0.01, *P* < 0.001,. *P* < 0.0001, ns *P* > 0.05)^*^^**^^***^^****^

The pathological types of these 98 cases of breast cancer were primary invasive carcinoma and non-specified type. RT-qPCR was used to detect the expression of BC069792 in these 98 pairs of breast cancer tissues and normal breast tissues. The results showed that the median expression level of BC069792 in breast cancer tissue was 0.013002, and that in normal breast tissue was 0.063384. Compared with the two groups, the difference in the expression level of lncRNA BC069792 was about 4.87 times different (Fig. 1a, *P* < 0.0001). The results of ROC curve analysis showed that the expression of BC069792 was able to distinguish breast cancer tissue from normal breast tissue, and the area under the curve was 0.9191 (Fig. 1b). This result suggested that BC069792 could be used as a molecular marker for breast cancer.

In breast cancer tissues, the expression of BC069792 was significantly decreased in those with high pathological grade, lymph node metastasis and high Ki-67 index groups. Spearman correlation analysis showed that BC069792 was negatively correlated with pathological grade, lymph node metastasis and high Ki-67 index, but had no significant correlation with age, tumor size, and hemorrhage/calcification/necrosis/cystic degeneration (Table 1).

### The localization and stability of BC069792

In order to determine the cell lines that can be used in the experiment, we extracted RNA from breast cancer cell lines MDA-MB-231, MDA-MB-453, MDA-MB-468, MCF-7 and non-tumor cell line MCF-10A, and carried out RT-qPCR experiment after reverse transcription. The results showed that in breast cancer cell lines MDA-MB-231, MDA-MB-453, MDA-MB-468, MCF-7 and non-tumor cell line MCF-10A, the expression level was relatively low. Because triple negative breast cancer was difficult to treat in clinics, so we selected MDA-MB-231 and MDA-MB-468 cell lines for further experiments, In addition, the results showed that there was no significant difference in BC069792 expression between breast cancer cell lines and non-tumor cell lines (Supplementary Fig. 1a).

RT-qPCR with GAPDH as the internal reference for the cytoplasm and U6 as the internal reference for the nucleus showed that about 27.6% of BC069792 was in the cytoplasm in the MDA-MB-231 cell line. In the MDA-MB-468 cell line, about 26.4% of BC069792 was expressed in the cytoplasm, and 73.6% was expressed in the nucleus, indicating that BC069792 plays roles in both the nucleus and the cytoplasm (Fig. 1c). In addition, RNA-FISH assay also showed that BC069792 was distributed in both nucleus and cytoplasm (Fig. 1d).

Actinomycin D is an inhibitor of RNA polymerase II. In order to detect the stability of BC069792, we performed dosing experiments with actinomycin D in MDA-MB-231 and MDA-MB-468 cells. The content of BC069792 in cells was detected by RT-qPCR at 0 h, 0.5 h, 1 h, 4 h, 8 h and 12 h after dosing. The results showed that compared with C-MYC, BC069792 exists stably for a longer time in MDA-MB-231 and MDA-MB-468 cells (Fig. 1e).

### BC069792 inhibits the proliferation, migration, and invasion abilities of breast cancer cells in vitro

The relationship between BC069792 and clinicopathological parameters suggests that BC069792 plays the role of tumor suppressor gene in breast cancer. In order to clarify the specific function of BC069792 in breast cancer, we performed a series of experiments in vitro.

**Fig. 2:**
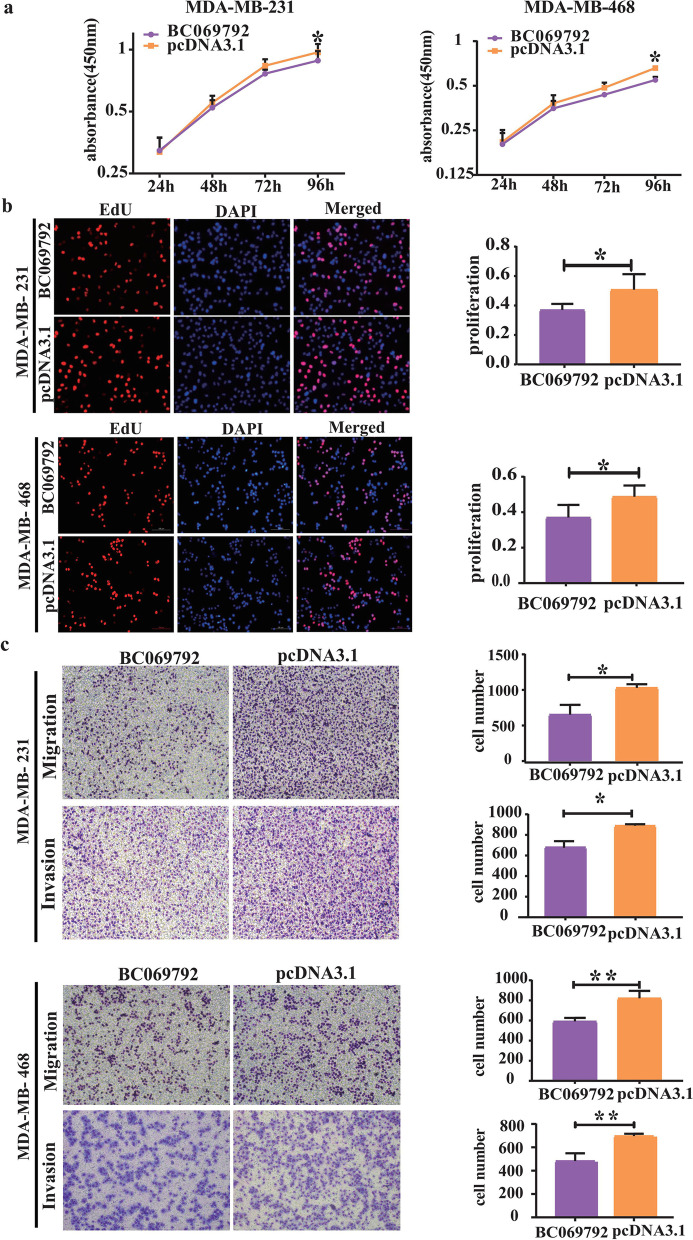
BC069792 inhibited the proliferative capacity of breast cancer cells. **a** The growth curve of CCK-8 assay showed that overexpression of BC069792 significantly inhibited the proliferation of MDA-MB-231 (*P* = 0.035) and MDA-MB-468 (*P* = 0.021) cells. **b** The results of EdU experiment showed that overexpression of BC069792 significantly inhibited the proliferation of MDA-MB-231 (*P* = 0.047) and MDA-MB-468 (*P* = 0.049) cells. **c** Overexpression of BC069792 inhibited the migration of MDA-MB-231 (*P* = 0.011) and MDA-MB-468 (*P* = 0.008) cells. It also inhibited the invasive ability of MDA-MB-231 (*P* = 0.020) and MDA-MB-468 (*P* = 0.006) cells (*P* < 0.05, *P* < 0.01, *P* < 0.001,. *P* < 0.0001, ns represent* P* > 0.05)^*^^**^^***^^****^

CCK-8 and EdU experiments showed that overexpression of BC069792 significantly inhibited the proliferation of MDA-MB-231 and MDA-MB-468 cells compared with the control group (Fig. 2a, b). After knocking down the expression of BC096792, the proliferation of MDA-MB-231 and MDA-MB-468 cells was slightly increased (Supplementary Fig. 1a, b). The results of the Transwell experiment (Fig. 2c) and wound healing assay (Supplementary Fig. 2) showed that compared with the control group, the number of BC069792 overexpressing cells crossing the basement membrane was significantly reduced, and the overexpression of BC069792 significantly inhibited the migration and invasion abilities of MDA-MB-231 and MDA-MB-468. Knockdown of BC069792 effectively promoted the migration and invasion of MDA-MB-231 and MDA-MB-468 cells, and the number of cells passing through the basement membrane in the chamber was significantly increased (Supplementary Fig. 1c).

### BC069792 inhibits the proliferation, invasion, and metastasis abilities of breast cancer cells in vivo

**Fig. 3:**
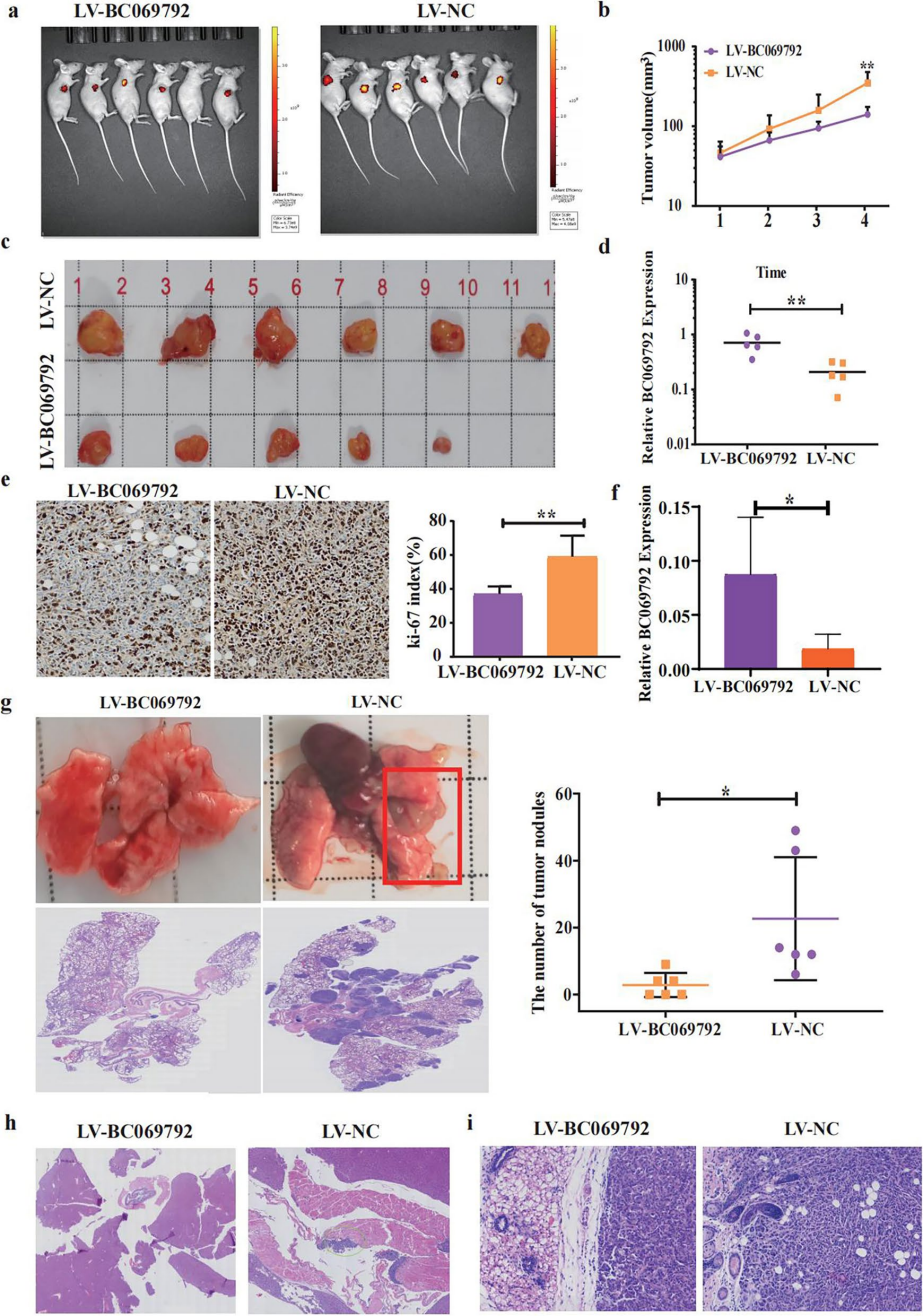
BC069792 inhibits the proliferation and invasion of breast cancer cells in vivo. **a** In vivo imaging of nude mice showed that the volume of the subcutaneous tumor in the LV-BC069792 group was significantly smaller than that of the LV-NC group. One nude mouse in the LV-BC069792 group had no tumor. **b** The growth curve showed that the tumor growth rate of the LV-NC group was significantly higher than that of the LV-BC069792 group (*P* = 0.0081). **c** The removal of subcutaneous tumors showed that the tumor volume in the LV-BC069792 group was significantly smaller than that of the LV-NC group, and the tumor of a nude mouse in the LV-BC069792 group were suppressed. **d** The content of BC069792 in tumor tissue in LV-BC069792 group was significantly higher than that in LV-NC group (*P* = 0.0051). **e** The Ki-67 index of the LV-BC069792 group was about 25%, and the Ki-67 index of the LV-NC group was about 80% (IHC × 200). The Ki-67 index of the LV-BC069792 group was significantly lower than that of the LV-NC group (*P* = 0.0059). **f** In lung metastasis, the expression of BC069792 in LV-BC069792 group was significantly higher than that in LV-NC group (*P* = 0.0228). **g** The gross image of the lung metastases in nude mice showed that the tumor growth at the hilum and outside the lung of the nude mice in the LV-NC group was obvious. In LV-BC069792 group, there were no obvious tumors in the lungs of nude mice. HE staining results showed that the lung metastases in the LV-NC group were significantly increased and the volume was significantly increased. The scatter plot showed that the number of metastases in the overexpression BC069792 group was significantly reduced (*P* = 0.0267). **h** There was liver-diaphragm metastasis in the LV-NC group, but no cancer metastasis in the liver and diaphragm of the LV-BC069792 group. **i** The tumor cells in the LV-NC group invaded the skin adnexa, while the tumor cells in the LV-BC069792 group had a clear boundary with the surrounding adipose tissue and mammary tubules, and no obvious invasion was found. (*P* < 0.05, *P* < 0.01, *P* < 0.001,.*P* < 0.0001, ns represent *P* > 0.05)^*^^**^^***^^****^

The subcutaneous tumorigenesis model of nude mice was successfully established by subcutaneous injection of lentivirus LV-BC069792 and LV-NC infected MDA-MB-231 cells. After 5 weeks, 5 out of 6 nude mice in the LV-BC069792 group developed tumors, and all 6 nude mice in the LV-NC group had tumors (Fig. 3a). The tumor growth curve showed that the tumor growth rate in the LV-NC group was significantly higher than that in the LV-BC069792 group (Fig. 3b), and the tumor size of the mice in the LV-BC069792 group was significantly smaller than that in the LV-NC group (Fig. 3c). By extracting RNA from tumor tissue of nude mice and performing RT-qPCR experiments, it was confirmed that the expression level of BC069792 in the LV-BC069792 group was significantly higher than that in the LV-NC group (Fig. 3d). Ki-67 index in the LV-BC069792 group was significantly lower than that in the LV-NC group (Fig. 3e). These experimental results demonstrated that overexpression of BC069792 inhibited the proliferation of breast cancer cells.

The lentivirus-infected MDA-MB-231 cells was injected into nude mice through the tail vein to construct a distant metastasis model. The results showed that 3 nude mice in the LV-BC069792 group had metastases in the lungs. The median number of metastases in the lungs of the nude mice was 2, and the size was small. In the LV-NC group, 6 nude mice were found to have metastases in the lungs, and the median number of metastases in the lungs of nude mice was 13, and tumor size was larger. The results of RT-PCR experiment showed that the expression of BC069792 in LV-BC069792 group was significantly higher than that in LV-NC group (Fig. 3f). In the LV-NC group, it was found that the tumor invaded the lung, and the liver of one of the nude mice adhered to the diaphragm, and breast cancer metastasis was seen in the diaphragm. In the LV-BC069792 group, no other metastases except the lung were found (Fig. 3g, h). In the subcutaneous xenograft model, the tumor infiltrated the fat, striated muscle and skin appendages in the LV-NC group, while the tumor in the LV-BC069792 group had a clearer boundary and formed a pseudocapsule without obvious invasion (Fig. 3i). The subcutaneous tumorigenesis and distant metastasis models in nude mice confirmed that BC069792 effectively inhibited the migration and invasion of breast cancer cells.

### The RNA-Seq reveals that KCNQ4 is an important downstream target gene of BC069792

To explore the downstream effector molecules regulated by BC069792, we performed transcriptome sequencing (RNA Sequence, RNA-Seq) in BC069792 overexpression group and control group. The results of principal component analysis showed that the two groups of samples submitted for inspection had a certain degree of distinction, and the consistency within each group was high (Supplementary Fig. 3a). A total of 23,365 genes were detected by sequencing. Compared with the pcDNA3.1 group, overexpression of BC069792 caused differential expression of 1209 genes (*p* < 0.05, the log_2_ difference multiple of more than 2 times). A total of 407 genes were up-regulated and 802 genes were down-regulated (Supplementary Fig. 3b). According to the differential genes obtained by sequencing, GO enrichment analysis and KEGG enrichment analysis were performed, and a total of 212 pathways were analyzed. Compared with the control group, 25 pathways were significantly changed in the BC069792 overexpression group (*P* < 0.05), of which 7 pathways were concentrated on synaptic transmission and signal transduction pathways (Supplementary Fig. 3c).

**Fig. 4:**
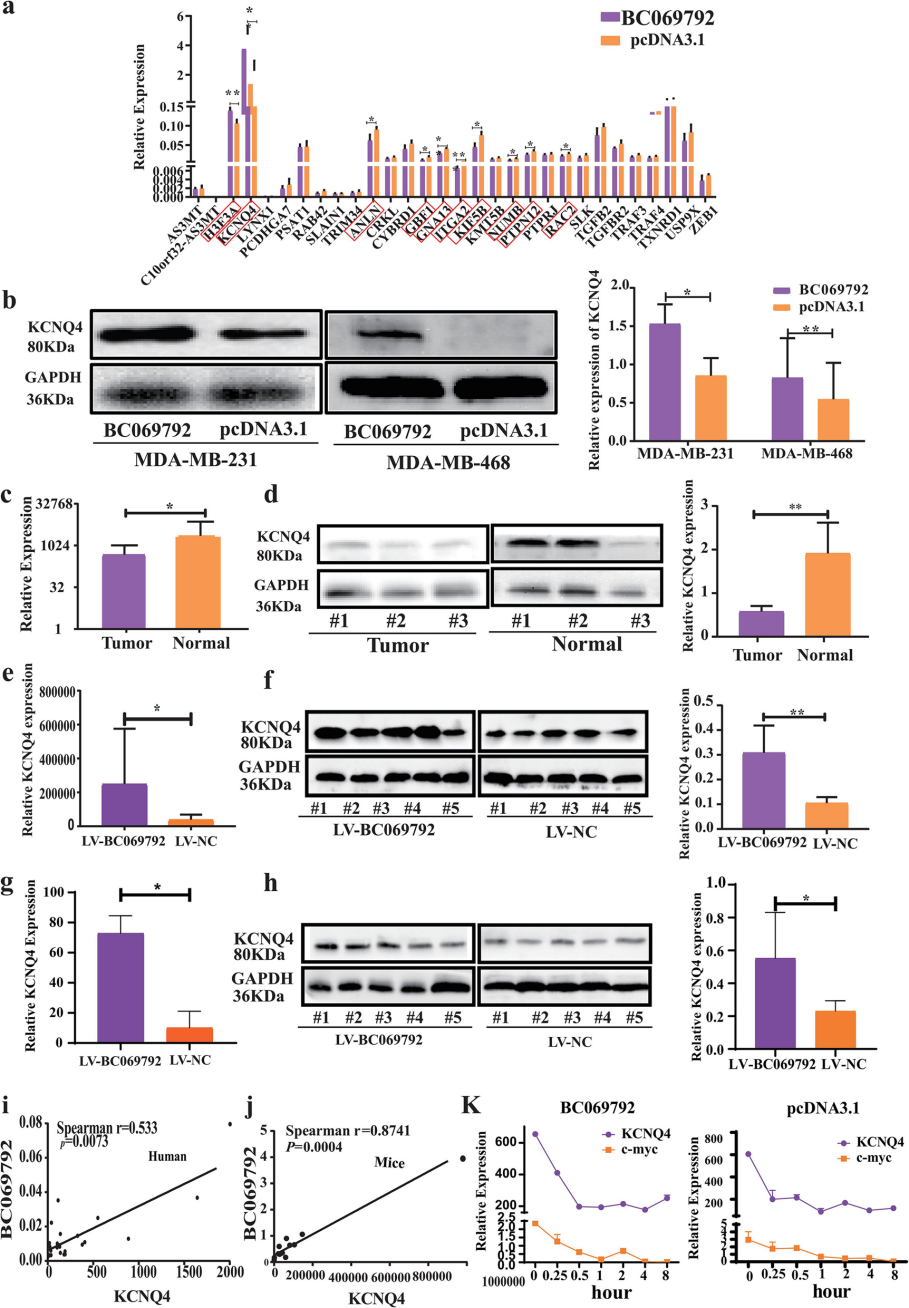
KCNQ4 is a downstream target gene of BC069792. **a** RT-qPCR results showed that overexpression of BC069792 affected the expression of multiple genes in MDA-MB-231 cells, among which the increase of KCNQ4 was the most significant. **b** Western blot showed that the expression of KCNQ4 protein in the BC069792 overexpression group was significantly higher than that in the control group (MDA-MB-231: *P* = 0.0271, MDA-MB-468: *P* = 0.0088). **c** The mRNA expression of KCNQ4 in normal breast tissue was significantly higher than that in breast cancer tissue (*P* = 0.0145). **d** The protein expression of KCNQ4 in normal breast tissue was significantly higher than that in breast cancer tissue (*P* = 0.031). **e **and** f** In axillary tumors of mice, the expression of mRNAand protein of KCNQ4 in LV-BC069792 group was significantly higher than that in LV-NC group. **g **and** h** In lung metastasesof mice, the expression of mRNAand proteinof KCNQ4 in LV-BC069792 group was significantly higher than that in LV-NC group. **i** The content of KCNQ4 protein in LV-BC069792 tumor tissues of nude mice was significantly higher than that in LV-NC nude mice (*P* = 0.0051). **j** In human tumor tissue and nude mouse tumor tissue, BC069792 and KCNQ4 had a significant positive correlation. **k** BC069792 can promote the RNA stability of KCNQ4.(*P* < 0.05, *P* < 0.01, *P* < 0.001,.*P* < 0.0001, ns represent* P* > 0.05)^*^^**^^***^^****^

According to the mRNA expression level, the log_2_ difference multiple of more than 2 times and *p* < 0.05 in the sequencing results, 30 genes were selected from the sequencing results to be verified. RT-qPCR showed that among the 30 genes, the expression of KCNQ4 was the most significantly different between BC069792 overexpression group and control group and had highest expression level in MDA-MB-231 cells (Fig. 4a). The expression of KCNQ4 protein also increased after overexpression of BC069792 (Fig. 4b) but decreased in the BC069792 knockdown cells (Supplementary Fig. 4). Moreover, the mRNA (Fig. 4c) and protein (Fig. 4d) expression of KCNQ4 in normal breast tissue was significantly higher than that in breast cancer tissue. In axillary tumors (Fig. 4e, f) and lung metastases(Fig. 4g, h) of mice, the expression of mRNAand proteinof KCNQ4 in LV-BC069792 group was significantly higher than that in LV-NC group. And the expression of BC069792 was positively correlated with KCNQ4 (Fig. 4i, j). BC069792 can promote the RNA stability of KCNQ4 (Fig. 4k).The above findings indicated that BC069792 regulates the mRNA and protein expression of KCNQ4 in breast cancer, suggesting that BC069792 may inhibit the progression of breast cancer by promoting the expression of KCNQ4.

### BC069792, as an endogenous competing RNA (ceRNA), upregulates the expression of target gene KCNQ4 by adsorbing miR-658 and miR-4739

**Fig. 5:**
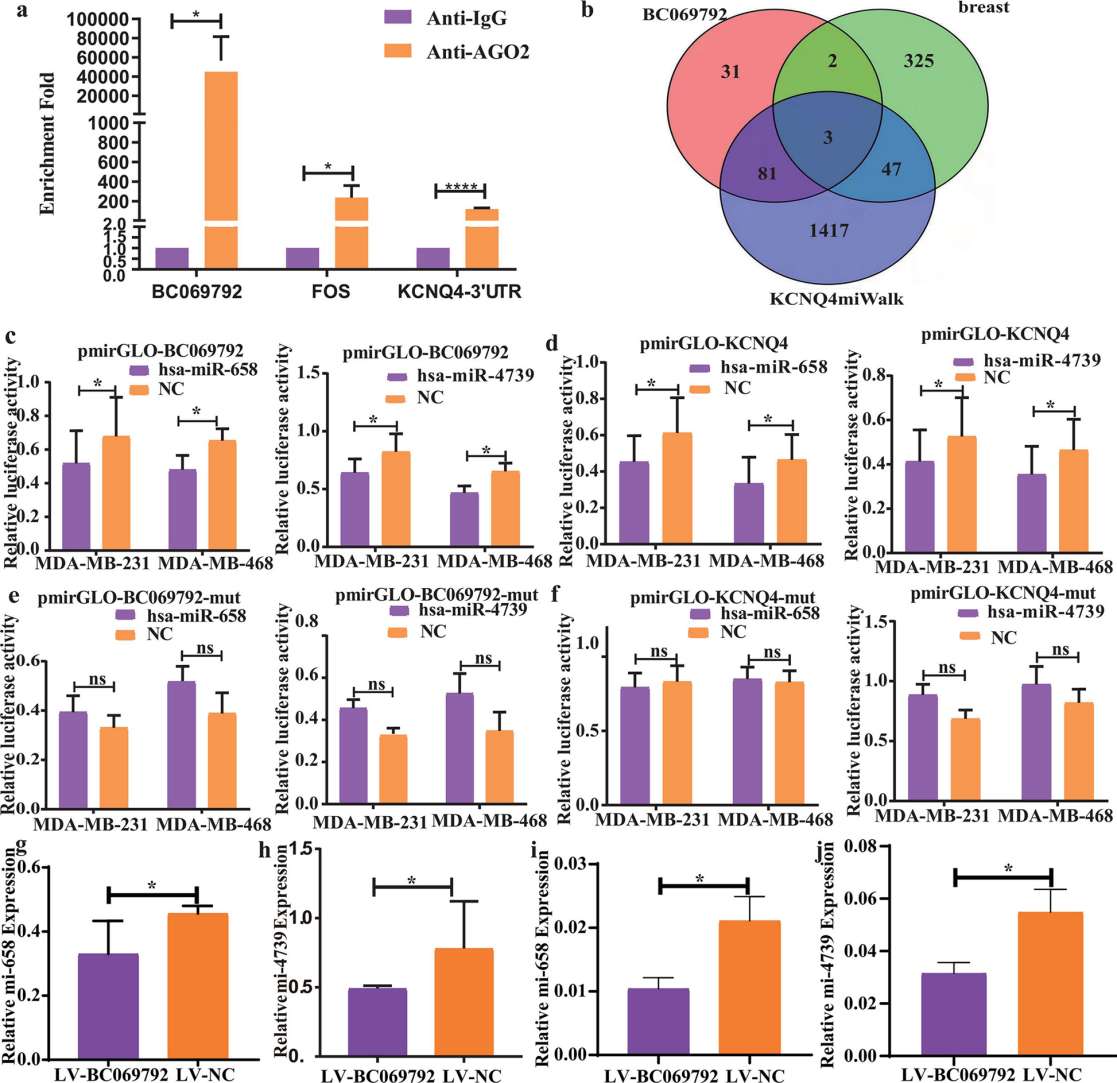
BC069792 upregulates the expression of KCNQ4 by binding to miR-658 and miR-4739. **a** Compared with the IgG control group, endogenous BC069792 and KCNQ4-3'UTR were specifically enriched by Ago2 antibody (BC069792: *P* = 0.017; FOS: *P* = 0.012; KCNQ4-3'UTR:*P* < 0.0001). **b** Through website prediction, database screening and literature query, three miRNAs were identified for further research. **c** After co-transfection of pmirGLO-BC069792 and mimics in MDA-MB-231 and MDA-MB-468 cell lines, miR-658 (MDA-MB-231: *P* = 0.021, MDA-MB-468: *P* = 0.037) and miR-658 -4739 (MDA-MB-231: *P* = 0.019; MDA-MB-468: *P* = 0.029) bound to pmirGLO-BC069792 and significantly inhibit luciferase activity. **d** After co-transfection of pmirGLO-KCNQ4 3'UTR and mimics, both miR-658 (MDA-MB- 231:*P* = 0.032, MDA-MB- 468:*P* = 0.017) and miR-4739(MDA-MB-231: *P* = 0.031; MDA-MB-468:*P* = 0.046) bound to pmirGLO-KCNQ4 3'UTR and reduced dual-luciferase activity. **e** There was no significant difference in luciferase activity between the experimental group and the control group after co-transfection of pmirGLO-BC069792-mut (miR-658 binding site mutation) and miR-658 mimics (MDA-MB-231: *P* = 0.253; MDA-MB-468: *P* = 0.152). After co-transfection of pmirGLO-BC069792-mut (miR-4739 binding site mutation) and miR-4739 mimics, there was no significant difference in luciferase activity between the experimental group and the control group (MDA-MB-231: *P* = 0.194; MDA- MB-468: *P* = 0.084). **f** There was no significant difference in luciferase activity between the experimental group and the control group after co-transfection of pmirGLO-KCNQ4-3'UTR-mut (miR-658 binding site mutation) and miR-658 mimics (MDA-MB-231: *P* = 0.160; MDA-MB-468: *P* = 0.699). After co-transfection of pmirGLO-KCNQ4-3'UTR-mut (miR-4739 binding site mutation) and miR-4739 mimics, there was no significant difference in luciferase activity between the experimental group and the control group (MDA-MB-231:*P* = 0.210; MDA-MB-468: *P* = 0.229).**g-j** In axillary tumors and lung metastases of mice, the expression of miR-658 and miR-4739 in LV-BC069792 group was lower than that in LV-NC group

Argonaute2 protein (Ago2) plays an important role in the occurrence and development of human tumors by participating in the formation of RNA-induced silencing complexes [15]. We performed RIP experiments with IgG as a control. The results showed that endogenous BC069792 and KCNQ4-3'UTR were specifically enriched by Ago2 antibody (Fig. 5a), suggesting that BC069792 and KCNQ4 can bind to specific miRNAs in the cytoplasm. The target miRNAs that can bind to BC069792 and KCNQ4 were predicted through miRWalk, RegRNA2.0 and other websites and ENCORI database, and the differentially expressed miRNAs in breast cancer and normal breast tissues were screened through the TCGA database. Three pro-tumor miRNAs, including miR-658, miR-632, and miR-4739 were found in the intersection of the three groups (Fig. 5b). The mimics or mimics NC of pmirGLO-BC069792/pmirGLO-NC and three miRNAs were co-transfected into MDA-MB-231 and MDA-MB-468 cell lines, and the fluorescein in the cells was detected by a microplate reader 48 h later. Compared with the control group, miR-658 and miR-4739 significantly inhibited the activity of pmirGLO-BC069792 luciferase (Fig. 5c), but not miR-632. Finally, the mimics of miR-658 and miR-4739 and pmirGLO-KCNQ4 3'UTR plasmids were co-transfected into MDA-MB-231 and MDA-MB-468 cells, and the fluorescence in the cells was detected by a microplate reader 48 h later. Both miR-658 and miR-4739 were found to reduce pmirGLO-KCNQ4 3'UTR dual-luciferase activity (Fig. 5d). To demonstrate the specificity of miR-658 or miR-4739 binding to BC069792 and KCNQ4, we performed the above experiments again after mutating the binding sites. The results showed that miR-658 and miR-4739 were no longer able to reduce the luciferase activity of pmirGLO-BC069792 and pmirGLO-KCNQ4 3'UTR after the mutation of the binding sites (Fig. 5e-f). In axillary tumors and lung metastases of mice, the expression of miR-658 and miR-4739 in LV-BC069792 group was lower than that in LV-NC group (Fig. 5g-j). The above results indicated that miR-658 and miR-4739 could specifically bind to the 3'UTR of BC069792 and KCNQ4. When BC069792 and KCNQ4 3'UTR mutations deleted the miR-658 and miR-4739 binding sites, they could not bind to miR-658 and miR-4739. These results indicated that BC069792 adsorbed miR-658 and miR-4739 as a sponge, and relieved their inhibition on the target gene KCNQ4, thereby up-regulating the expression of KCNQ4.

### BC069792 upregulates KCNQ4 and thus inhibits JAK2 and AKT phosphorylation to suppress breast cancer progression

**Fig. 6:**
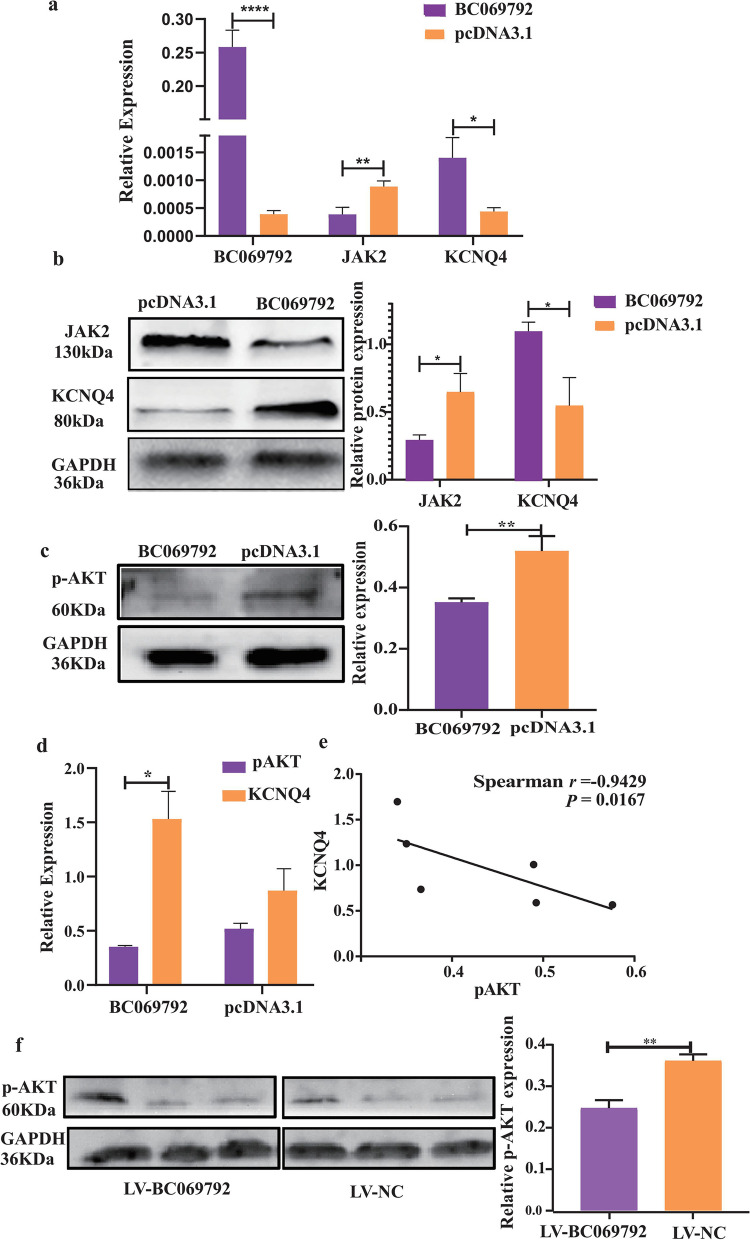
The downstream effector molecule regulated by KCNQ4 is JAK2/p-AKT. **a** After overexpression of BC069792, the mRNA expression of KCNQ4 was positively correlated with BC069792 and negatively correlated with JAK2. **b** After overexpression of BC069792, the protein expression of KCNQ4 was positively correlated with BC069792 and negatively correlated with JAK2. ** c** In MDA-MB-231 (*P* = 0.0047) cell lines, overexpression of BC069792 reduced the expression of p-AKT protein. **d** In the BC069792 overexpression MDA-MB-231 cells, the protein content of KCNQ4 was significantly higher than that of p-AKT (*P* = 0.032), while the content of KCNQ4 and p-AKT in the pcDNA3.1 group was not statistically significant (*P* = 0.194). **e** In MDA-MB-231 cells, KCNQ4 was negatively correlated with p-AKT(*r* = -0.943, *P* = 0.017). **f** The expression of p-AKT protein in tumor tissue of nude mice was significantly different between LV-BC06972 group and LV-NC group, and it was highly expressed in LV-NC group(*P* = 0.001

According to our RNA-Seq data, BC069792 on cholinergic synapses (hsa04725) increased the expression of KCNQ4 (control group 33.37, experimental group 67.12, *P* = 0.011), PIK3R3 (control group 48.19, experiment group 28.25, *P* = 0.015), and AKT (control group 930.34, experiment group 711.99, *P* = 0.0003). It is well known that PI3K/AKT is a classical tumor signaling transduction pathway that plays a crucial role in tumor proliferation, invasion and metastasis. An increasing number of studies have reported that the ion channel function is closely related to the growth of tumors [16, 17]. The human ether-a-go-go-related gene1 (hERG1) potassium channel (aka KV11.1) regulates p-AKT through the formation of hERG1 integrin and phosphatidylinositol-3 kinase p85 subunit macromolecular complex on the plasma membrane, leading to AKT activation, thus promoting the development of human colorectal cancer cells [18, 19]. KCNQ4 is closely related to the non-receptor tyrosine protein kinase JAK 2, which reduces the activity of KCNQ4 in Xenopus oocytes [20], while PI3K/AKT is up-regulated by JAK2 [21]. As reported in literature, hsa04725 pathway was negatively correlated with breast cancer recurrence rate [22]. Our results showed that the mRNA expression of KCNQ4 was positively correlated with BC069792 and negatively correlated with JAK2, and the protein expression of BC069792 was positively correlated with KCNQ4 and negatively correlated with JAK2 (Fig. 6a, b). In MDA-MB-231 cell lines, overexpression of BC069792 significantly decreased the expression of p-AKT (Fig. 6c). The protein expression of KCNQ4 in the same group of samples was significantly higher than that of p-AKT (Fig. 6d). Combined with the results of KCNQ4 and p-AKT Western Blot detection in the BC069792 overexpression group and the control group, the spearman correlation test was performed, and it was found that KCNQ4 was negatively correlated with p-AKT (Fig. 6e). Compared with the LV-NC group, the expression of p-AKT was significantly decreased in the LV-BC069792 group (Fig. 6f). These results indicated that BC069792 inhibited the expression of JAK2 protein and the phosphorylation of AKT protein by up-regulating KCNQ4, decreased the expression of p-AKT, and then inhibited the progression of breast cancer.

**Fig. 7:**
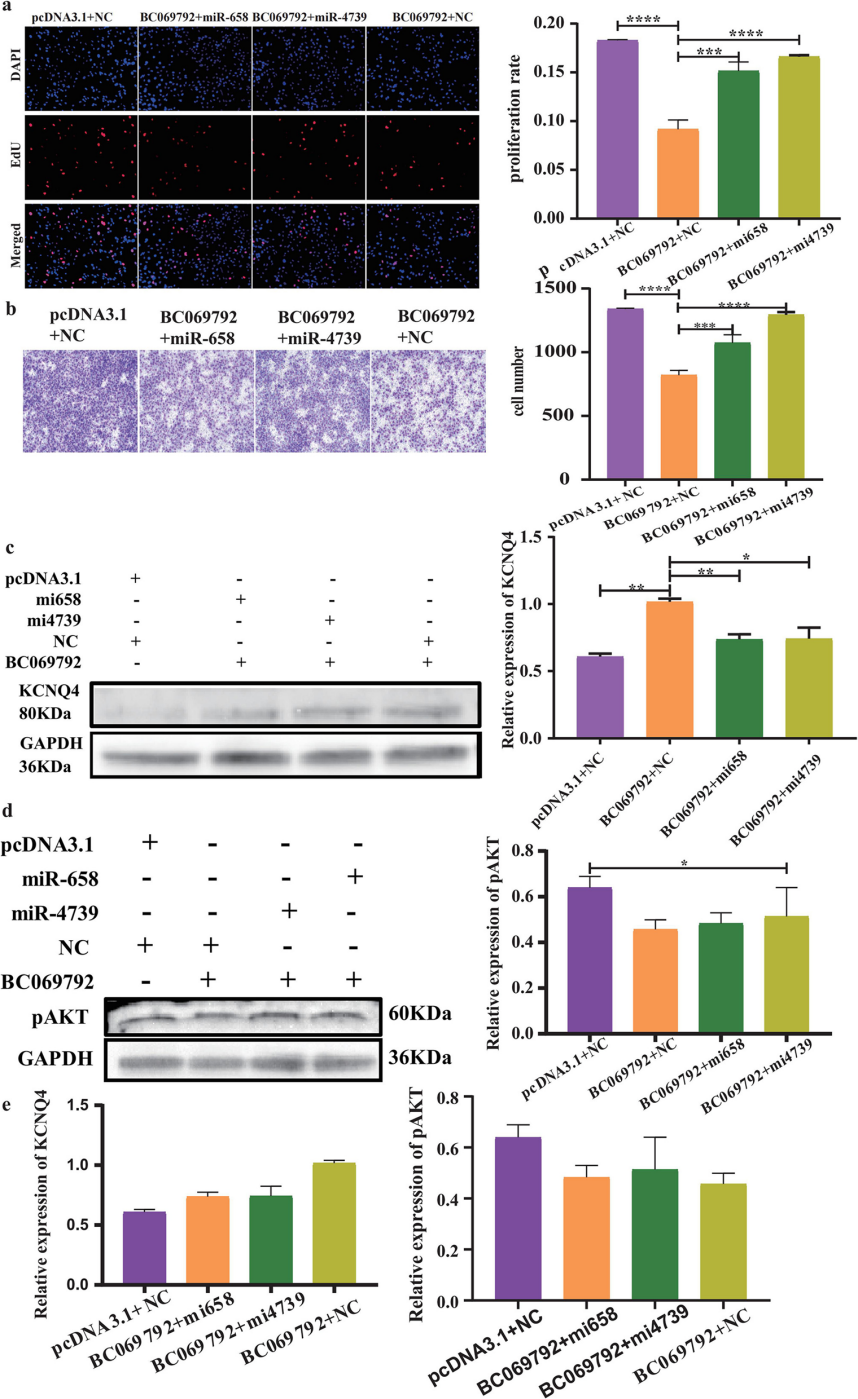
Rescue experiment. ** a** Overexpression of BC069792 inhibits the proliferation of breast cancer cells (*P* < 0.0001), After simultaneously overexpressing miR-658 mimics (*P* = 0.0002) and miR-4739 mimics (*P* < 0.0001), the proliferation of breast cancer cells was enhanced. **b** Overexpression of BC069792 inhibited the migratory ability of breast cancer cells (*P* < 0.0001). breast cancer cells have enhanced migratory ability after transfection with miR-658mimics (*P* = 0.0009) or miR-4739mimics (*P* < 0.0001). **c** After overexpression of BC069792 and simultaneous transfection of miR-658 mimics (*P* = 0.005) or miR-4739 mimics (*P* = 0.026), the elevated KCNQ4 protein expression level was decreased. **d** The p-AKT protein level was elevated in rescue experiments. **e** The negative correlation of KCNQ4 with p-AKT was restored. (:*P* < 0.05, :*P* < 0.01, :*P* < 0.001,.:*P* < 0.0001, ns:* P* > 0.05)^*^^**^^***^^****^

To further verify whether BC069792 exerts biological functions through competitive adsorption of hsa-miR-658 and hsa-miR-4739, we performed rescue experiments. The results showed that breast cancer cells overexpressed BC069792 with reduced proliferation ability, weakened migration ability, and increased KCNQ4 protein expression level. When BC069792 was simultaneously overexpressed with hsa-miR-658 or hsa-miR-4739, the inhibition of breast cancer cell proliferation and migration by BC069792 was reversed (Fig. 7a, b), the elevated KCNQ4 protein expression level was decreased (Fig. 7c), and the level of reduced p-AKT protein was increased (Fig. 7d). Spearman's correlation test showed that the negative correlation of KCNQ4 with p-AKT was restored (Fig. 7e). Based on the above experimental results, it is suggested that BC069792 exerts its biological functions by binding to miRNA, and can act as a molecular sponge to adsorb hsa-miR-658 or hsa-miR-4739, up-regulate the protein expression of the target gene KCNQ4, inhibit the activity of p-AKT, and then play a role in inhibiting breast cancer.

## Discussion

Long non-coding RNAs (lncRNAs) are RNAs with a length greater than 200 nucleotides. LncRNAs have no open reading frames (ORF) and no coding function. Studies have shown that lncRNAs are involved in a number of activities such as epigenetic regulation, transcriptional regulation, and post-transcriptional regulation, and play a tumor-promoting or tumor-suppressing role in the occurrence and development of tumors. A variety of lncRNAs have been found to be dysregulated in breast cancer, and their expression levels are different in breast cancer tissues of different molecular subtypes [23, 24].

Our previous study found that lncRNA BC069792 acts as a tumor suppressor gene in gastric cancer. In order to verify whether BC069792 has the role of tumor suppressor gene in breast cancer, we detected its expression in breast cancer and breast epithelial tissue. It was found that the expression of BC069792 in breast cancer was significantly decreased, and was negatively correlated with pathological grade, lymph node metastasis and proliferation index, indicating that BC069792 has tumor suppressor effect in breast cancer and can be used as a biological indicator of poor prognosis of breast cancer.

A study has shown that abnormally expressed lncRNAs in tumor tissues have different regulatory effects on tumor cell proliferation, apoptosis, invasion and metastasis [25]. Zhao et al. found that lncRNA HOTAIR promotes the proliferation and metastasis of breast cancer cells [26], Zhou et al. found that lncRNA SPINT1-AS1 promotes the proliferation and metastasis of breast cancer by sponging let-7 a/b/i-5p [27]. In order to verify the role of BC069792 in breast cancer cells, we performed cell function tests. The results showed that BC069792 inhibited the proliferation, migration and infiltration of breast cancer cells both in vitro and in vivo, verifying the role of BC069792 as a tumor suppressor gene in breast cancer.

In order to explore the mechanism of lncRNA BC069792 in inhibiting breast cancer proliferation and metastasis, we performed RNA transcriptome high-throughput gene sequencing and KEGG pathway enrichment analysis. BC069792 was found to be closely related to changes in synaptic transmission and signal transduction pathways. This result provides important clues for the selection of downstream genes of BC069792 in inhibiting the proliferation, invasion and migration of breast cancer cells. Through screening, we found that the expression of KCNQ4 was mostly affected by BC069792. From this finding, we further investigated whether KCNQ4 is the target gene of BC069792 in breast cancer, and whether BC069792 exerts its tumor suppressor effect by regulating KCNQ4.

KCNQ4 was named after its first discovery by Kubisch et al. in 1999, also known as the voltage-gated potassium channel subunit Kv7.4. KCNQ4 gene is a homologous tetramer, located on human chromosome 1 1p34.2 (chr1:41,249, 684–41, 306,124) [28]. It is one of the most complex and diverse ion channels found at cell membrane. It has been reported that voltage-gated potassium channels are closely related to the occurrence, proliferation and migration of various tumors [29, 30]. Than et al. [31] found that KCNQ1 was expressed at low level in colon cancer and significantly correlated with patient survival. KCNQ1 knockout in a nude mouse model of colon cancer leads to cancer progression, suggesting that KCNQ1 may function as a tumor suppressor. The expression of Kv3.4 may be related to the occurrence and development of oral squamous cell carcinoma [32]. Santos et al. [33] found that the expression of KCNQ4 was decreased in prostate cancer. Our results were in consistent with the above findings. These research results provide new ideas for using KCNQ4 as a novel therapeutic target for cancer.

The lncRNA-associated ceRNA network plays an important role in the initiation and progression of multiple pathological mechanisms. There are two keywords in the concept of ceRNA, competition and endogenous. CeRNA can be regarded as a kind of balance, when the balance is broken, it will lead to the occurrence of diseases such as tumors. We used bioinformatics analysis to predict miRNAs that could bind to both BC069792 and KCNQ4, and validated the potential interaction of BC069792-miRNA-KCNQ4. The experimental results showed that BC069792 could act as a ceRNA by adsorbing miR-658 and miR-4739, thereby inhibiting the binding of miR-658 and miR-4739 to KCNQ4 mRNA, and up-regulating the expression of the target gene KCNQ4. Further rescue experiments also demonstrated the regulatory network of BC069792-miRNA-KCNQ4. This ceRNA network can provide a basis for new therapeutic regimens and drug development.

MiR-658 and miR-4739 are conserved ncRNA molecules. We demonstrated that miR-658 or miR-4739 significantly promoted the proliferation and migration of MDA-MB-231 and MDA-MB-468 breast cancer cells, which is consistent with the findings of miR-658 in gastric cancer metastasis [34] and in promoting the proliferation of lung cancer PC-9/ZD and PC-9 cells [35]. miR-4739 is also involved in the oncogenic activation pathway of gastric cancer [36]. This study demonstrated that miR-658 or miR-4739 inhibited the expression of KCNQ4 protein in breast cancer cells, and the expression of miR-658 or miR-4739 was negatively correlated with the expression of KCNQ4 in human and nude mouse breast cancer tissues. The rescue experiments showed that when BC069792 was overexpressed and miR-658 or miR-4739 was transfected simultaneously, the proliferation, invasion and migration abilities of breast cancer cells were reversed, and the expression level of elevated KCNQ4 protein was decreased. These experimental results suggest that miR-658 or miR-4739 can promote cancer by reducing the expression of KCNQ4 in breast cancer. It can be seen that BC069792 acts as a ceRNA to relieve its inhibition on the target gene KCNQ4 by adsorbing miR-658 or miR-4739, and upregulate the expression of KCNQ4 to exert a tumor suppressor effect, providing a new mechanism of post-transcriptional regulation of BC069792.

**Fig. 8:**
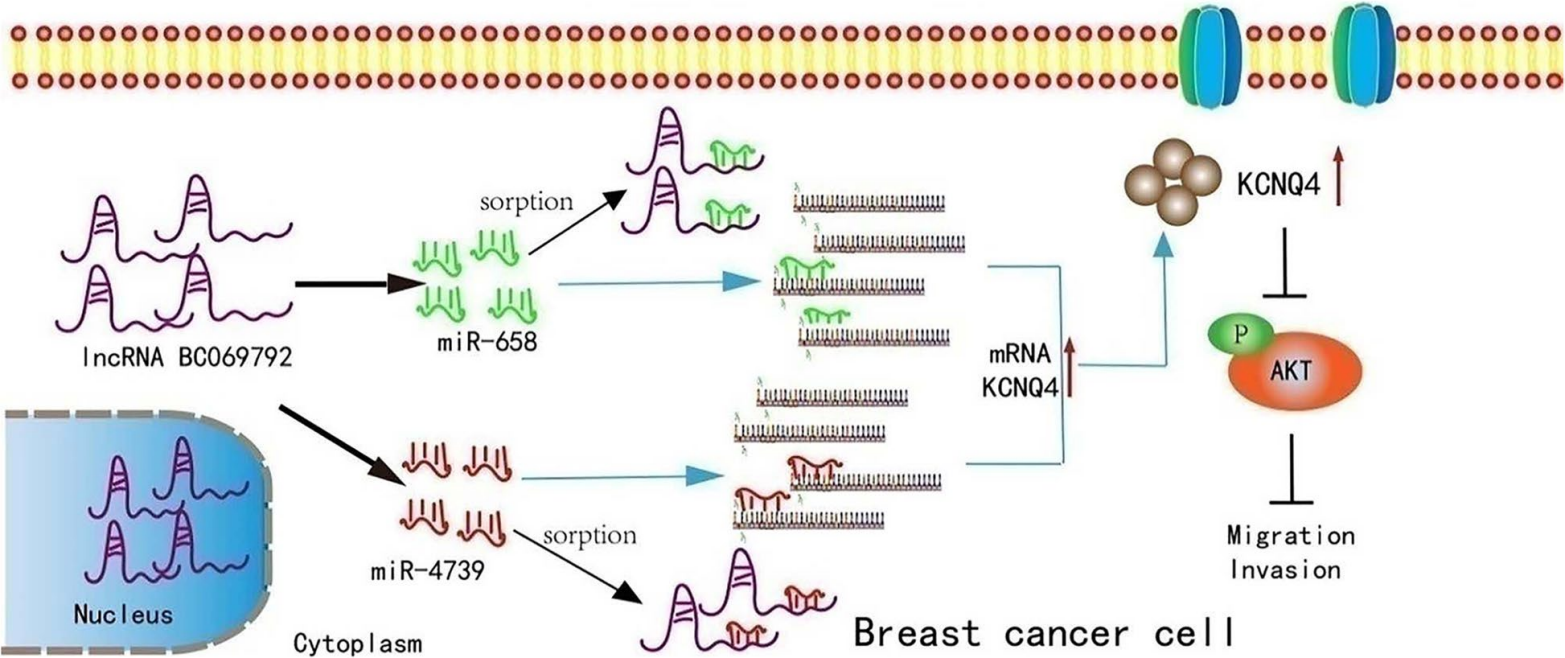
The mechanism of lncRNA BC069792 to suppress tumor progression in breast cancer. Inc RNA BC069792 is expressed in both the cytoplasm and nucleus, which acts as a ceRNA sponge adsorbing miR-658 and miR-4739 and upregulates the transmembrane protein KCNQ4 expression, thereby inhibiting AKT phosphorylation and inhibiting the proliferation and metastasis of breast cancer

In this study, based on the literature [16, 17, 19–21], combined with the results of high-throughput sequencing and KEGG pathway analysis, it was found that the differential expression of KCNQ4 was involved in the AKT signal transduction pathway. The p-AKT level in the BC069792 overexpressing breast cancer cell group was significantly lower than that in the control group. Furthermore, compared with the control group, the level of p-AKT in the BC069792 overexpressing cells in nude mice was also significantly decreased. The expression of p-AKT was negatively correlated with the expression of KCNQ4 and BC069792, and the expression of KCNQ4 and BC069792 was positively correlated. It is inferred that AKT is a downstream signaling molecule of KCNQ4. Serine/threonine kinase AKT (PKB), as a proto-oncogene, plays a role in promoting the proliferation, invasion and metastasis of various tumor cells [37–39]. The rescue experiment results showed that when overexpressing BC069792 and transfecting miR-658mimics or miR-4739mimics, the expression level of the increased KCNQ4 protein was decreased, and the expression level of the decreased p-AKT protein was increased. It is well known that the PI3K/AKT signaling pathway plays a crucial role in tumor proliferation, invasion and metastasis, and is a classic tumor signal transduction pathway. Based on the above experiments, we infer that KCNQ4 can inhibit tumor proliferation, invasion and metastasis by inhibiting the activity of p-AKT. This result is also consistent with the RNA-Seq detection data which showed that KCNQ4, PI3K and AKT are located in the cholinergic synaptic pathway (hsa04725). It is also consistent with the research report that the cholinergic synaptic pathway (hsa04725) is negatively correlated with breast cancer recurrence [22]. Combined with literature studies, the KV11.1 channel, the hERG1 channel inhibitor E4031, can restore the up-regulated p-AKT in colorectal cancer animal models to inhibit the development of colorectal cancer [17], JAK2 can down-regulate the expression of KCNQ4 [21], and PI3K/AKT can be up-regulated by JAK2 [22], further demonstrating that KCNQ4 can inhibit the progression of breast cancer cells by inhibiting the activity of p-AKT(Fig. 8).

## Conclusions

The expression of lncRNA BC069792 was low in breast cancer, and decreased significantly in cancer tissues with high histological grade, lymph node metastasis and high Ki-67 index group. BC069792 can inhibit the proliferation, invasion and metastasis of breast cancer cells in vivo and in vitro by inhibiting the proliferation, invasion and metastasis of breast cancer through the ceRNA regulatory network of BC069792-hsa-miR-658/miR-4739-KCNQ-JAK2-AKT.

We discovered for the first time that lncRNA could target potassium channel proteins to induce AKT phosphorylation. On this basis, it would be interested to study the action mechanism of potassium channel KCNQ4 and its role in cancer. Finding effective potassium channel target therapy will be our focus of future research.

## Supplementary Information

**Additional file 1:** **Supplementary Fig. 1.** a In breast cancer cell line and non-tumor cell line MCF-10A, the expression of BC069792 was the highest in non-tumor cell line MCF-10A, while in breast cancer cell line, the expression of BC069792 in MDA-MB-231 and MDA-MB-468 cell lines was the lowest. b CCK-8 experiments showed that si-BC069792 can promote the proliferation of MDA-MB-231 cells (*P=0.43) and MDA-MB-468 (*P=0.026) cells. c The results of EdU experiments showed that si-BC069792 promoted the proliferation ability of breast MDA-MB-231 (*P=0.042) and MDA-MB-468 (*P=0.050) cancer cells. d Compared with the control group, the si-BC069792 knockdown group can effectively promote the migration (*P*=0.044) and invasion ability (*P*=0.002) of MDA-MB-231 cells, while the si-BC069792 knockdown group can effectively promote the migration (*P*=0.002) and invasion (**P=0.005) of MDA-MB-468 cells, and the number of cells passing through the underfloor membrane of the chamber is significantly increased. *P< 0.05, **P< 0.01, ***P< 0.001. **Supplementary Fig. 2.** Wound healing experiment confirmed that BC069792 can effectively inhibit the migration ability of breast cancer cells. ** Supplementary Fig.** **3.** Gene differential expression results after breast cancer cells overexpressed BC069792 a The results of principal component analysis showed that the consistency within the two sample groups was good and had difference. b The results of gene difference analysis showed that the BC069792 overexpression group could cause differential expression of 1209 downstream genes. c The differential expression pathway shown in the figure related to the transduction function of synaptic transmission signal. **Supplementary Fig. 4.** The exprssion of KCNQ4 protein in the knockdown BC069792 group was significantly reduced (*p=0.014).

## Data Availability

The datasets used and/or analyzed during the current study are available from the corresponding author on reasonable request.

## Abbreviations

IncRNAs: Long non-coding RNAs
ceRNA: Competing endogenous RNA
RT-qPCR: Real-time fluorescence quantitative polymerase chain reaction
CCK-8: Cell Counting Kit-8
h: Hour
RNA-seq: RNA-sequencing
KCNQ4: Potassium Voltage-Gated Channel subfamily q member 4
PI3K: Phosphoinositide3-Kinase
ACT: Protein kinase B
GAPDH: Glyceraldehyde-3-phosphate dehydrogenase
RIP: RNA Immunoprecipitation
ROC: Receiver Operating Characteristic
ns: No significance
GO: Gene Ontology
KEGG: Kyoto Encyclopedia of Genes and Genomes
IgG: Immunoglobulin G
Ago2: Argonaute2 protein
UTR: Untranslated region
ENCORI: The Encyclopedia of RNA Interactomes
JAK2: Janus Kinase 2
ORF: Open reading frames

## References

[1] Sung H, Ferlay J, Siegel RL, Laversanne M, Soerjomataram I, Jemal A (2021). Global Cancer Statistics 2020: GLOBOCAN Estimates of Incidence and Mortality Worldwide for 36 Cancers in 185 Countries. CA Cancer J Clin.

[2] Siegel R, Miller K (2020). Jemal AJCacjfc. Cancer statistics.

[3] Siegel R, Miller K, Fuchs H (2021). Jemal AJCacjfc. Cancer Statistics.

[4] Schmitt AM, Chang HY (2016). Long Noncoding RNAs in Cancer Pathways. Cancer Cell.

[5] Statello L, Guo C-J, Chen L-L, Huarte M (2021). Gene regulation by long non-coding RNAs and its biological functions. Nat Rev Mol Cell Biol.

[6] Esposito R, Bosch N, Lanzos A, Polidori T, Pulido-Quetglas C, Johnson R (2019). Hacking the cancer genome: profiling therapeutically actionable long non-coding RNAs using CRISPR-Cas9 screening. Cancer Cell.

[7] Kim J, Piao HL, Kim BJ, Yao F, Han Z, Wang Y (2018). Long noncoding RNA MALAT1 suppresses breast cancer metastasis. Nat Genet.

[8] Yuan K, Lan J, Lin Xu, Feng X, Liao H, Xie K, Hong Wu, Zeng Y (2022). Long noncoding RNA TLNC1 promotes the growth and metastasis of liver cancer via inhibition of p53 signaling. Mol Cancer.

[9] Park MK, Zhang L, Min K-W, Cho J-H (2021). NEAT1 is essential for metabolic changes that promote breast cancer growth and metastasis. Cell Metab.

[10] Luo W, Wang J, Wenhao Xu (2021). LncRNA RP11-89 facilitates tumorigenesis and ferroptosis resistance through PROM2-activated iron export by sponging miR-129-5p in bladder cancer. Cell Death Dis.

[11] Zhang Y, Dong X, Wang Y, Wang L, Han G, Jin L, Fan Y, Xu G, Yuan D, Zheng J, Guo X, Gao P (2021). Overexpression of LncRNA BM466146 Predicts Better Prognosis of Breast Cancer. Front Oncol.

[12] Fan Y, Dong X, Li M, Liu P, Zheng J, Li H, Zhang Y (2022). LncRNA KRT19P3 Is Involved in Breast Cancer Cell Proliferation, Migration and Invasion. Front Oncol.

[13] Dai M, Huang W, Huang X, Ma C, Wang R, Tian P (2023). BPDE, the Migration and Invasion of Human Trophoblast Cells, and Occurrence of Miscarriage in Humans: Roles of a Novel lncRNA-HZ09. Environ Health Perspect.

[14] Xu M, Xu X, Pan B, Chen X, Lin K, Zeng K (2019). LncRNA SATB2-AS1 inhibits tumor metastasis and affects the tumor immune cell microenvironment in colorectal cancer by regulating SATB2. Mol Cancer.

[15] McKenzie AJ, Hoshino D, Hong NH, Cha DJ, Franklin JL, Coffey RJ (2016). KRAS-MEK Signaling Controls Ago2 Sorting into Exosomes. Cell Rep.

[16] Litan A, Langhans SA (2015). Cancer as a channelopathy: ion channels and pumps in tumor development and progression. Front Cell Neurosci.

[17] Pardo LA (2004). Voltage-gated potassium channels in cell proliferation. Physiology (Bethesda).

[18] Fiore A, Carraresi L, Morabito A, Polvani S, Fortunato A, Lastraioli E (2013). Characterization of hERG1 channel role in mouse colorectal carcinogenesis. Cancer Med.

[19] Crociani O, Zanieri F, Pillozzi S, Lastraioli E, Stefanini M, Fiore A (2013). hERG1 channels modulate integrin signaling to trigger angiogenesis and tumor progression in colorectal cancer. Sci Rep.

[20] Hosseinzadeh Z, Sopjani M, Pakladok T, Bhavsar SK, Lang F (2013). Downregulation of KCNQ4 by Janus kinase 2. J Membr Biol.

[21] Hou Y, Wang K, Wan W, Cheng Y, Pu X, Ye X (2018). Resveratrol provides neuroprotection by regulating the JAK2/STAT3/PI3K/AKT/mTOR pathway after stroke in rats. Genes Dis.

[22] Gao QG, Li ZM, Wu KQ (2013). Partial least squares based analysis of pathways in recurrent breast cancer. Eur Rev Med Pharmacol Sci.

[23] Vishnubalaji R, Shaath H, Elkord E, Alajez NM (2019). Long non-coding RNA (lncRNA) transcriptional landscape in breast cancer identifies LINC01614 as non-favorable prognostic biomarker regulated by TGFβ and focal adhesion kinase (FAK) signaling. Cell Death Discov.

[24] Jin X, Xu XE, Jiang YZ, Liu YR, Sun W, Guo YJ (2019). The endogenous retrovirus-derived long noncoding RNA TROJAN promotes triple-negative breast cancer progression via ZMYND8 degradation. Sci Adv.

[25] Pan J, Fang S, Tian H, Zhou C, Zhao X, Tian H et al. lncRNA JPX/miR-33a-5p/Twist1 axis regulates tumorigenesis and metastasis of lung cancer by activating Wnt/β-catenin signaling. Molecular cancer. 2020;19(1):9. 10.1186/s12943-020-1133-9 PMC6961326 31941509

[26] Zhao W, Geng D, Li S, Chen Z, Sun M (2018). LncRNA HOTAIR influences cell growth, migration, invasion, and apoptosis via the miR-20a-5p/HMGA2 axis in breast cancer. Cancer Med.

[27] Zhou T, Lin K, Nie J, Pan B, He B, Pan Y (2021). LncRNA SPINT1-AS1 promotes breast cancer proliferation and metastasis by sponging let-7 a/b/i-5p. Pathol Res Pract.

[28] Coucke P, Van Camp G, Djoyodiharjo B, Smith SD, Frants RR, Padberg GW (1994). Linkage of autosomal dominant hearing loss to the short arm of chromosome 1 in two families. N Engl J Med.

[29] Feng J, Yu J, Pan X, Li Z, Chen Z, Zhang W (2014). HERG1 functions as an oncogene in pancreatic cancer and is downregulated by miR-96. Oncotarget.

[30] Shao XD, Guo XZ, Ren LN, Lin H (2014). The mechanism of COX-2 regulating HERG channel in gastric cancer cells. Bratisl Lek Listy.

[31] Than BL, Goos JA, Sarver AL, O'Sullivan MG, Rod A, Starr TK (2014). The role of KCNQ1 in mouse and human gastrointestinal cancers. Oncogene.

[32] Fernández-Valle Á, Rodrigo JP, García-Pedrero JM, Rodríguez-Santamarta T, Allonca E, Lequerica-Fernández P (2016). Expression of the voltage-gated potassium channel Kv3.4 in oral leucoplakias and oral squamous cell carcinomas. Histopathology.

[33] Santos NJ, Camargo ACL, Carvalho HF, Justulin LA, Felisbino SL (2022). Prostate Cancer Secretome and Membrane Proteome from Pten Conditional Knockout Mice Identify Potential Biomarkers for Disease Progression. Int J Mol Sci.

[34] Wu Y, Wan X, Ji F, Song Z, Fang X (2018). Serum miR-658 induces metastasis of gastric cancer by activating PAX3-MET pathway: A population-based study. Cancer Biomark.

[35] Azuma Y, Yokobori T, Mogi A, Yajima T, Kosaka T, Iijima M (2020). Cancer exosomal microRNAs from gefitinib-resistant lung cancer cells cause therapeutic resistance in gefitinib-sensitive cells. Surg Today.

[36] Dong L, Deng J, Sun ZM, Pan AP, Xiang XJ, Zhang L (2015). Interference with the β-catenin gene in gastric cancer induces changes to the miRNA expression profile. Tumour Biol.

[37] Guo ZF, Kong FL (2021). Akt regulates RSK2 to alter phosphorylation level of H2A.X in breast cancer. Oncol Lett.

[38] Tang F, Wang Y, Hemmings BA, Rüegg C, Xue G (2018). PKB/Akt-dependent regulation of inflammation in cancer. Semin Cancer Biol.

[39] Savukaitytė A, Gudoitytė G, Bartnykaitė A, Ugenskienė R, Juozaitytė E (2021). siRNA Knockdown of REDD1 Facilitates Aspirin-Mediated Dephosphorylation of mTORC1 Target 4E-BP1 in MDA-MB-468 Human Breast Cancer Cell Line. Cancer Manag Res.

