# Supplementary figures and images for: LncRNA-BC069792 suppresses tumor progression by targeting KCNQ4 in breast cancer
Source: Mol Cancer. 2023 Mar 1;22:41. doi: 10.1186/s12943-023-01747-5 (PMC9976483; doi:10.1186/s12943-023-01747-5)

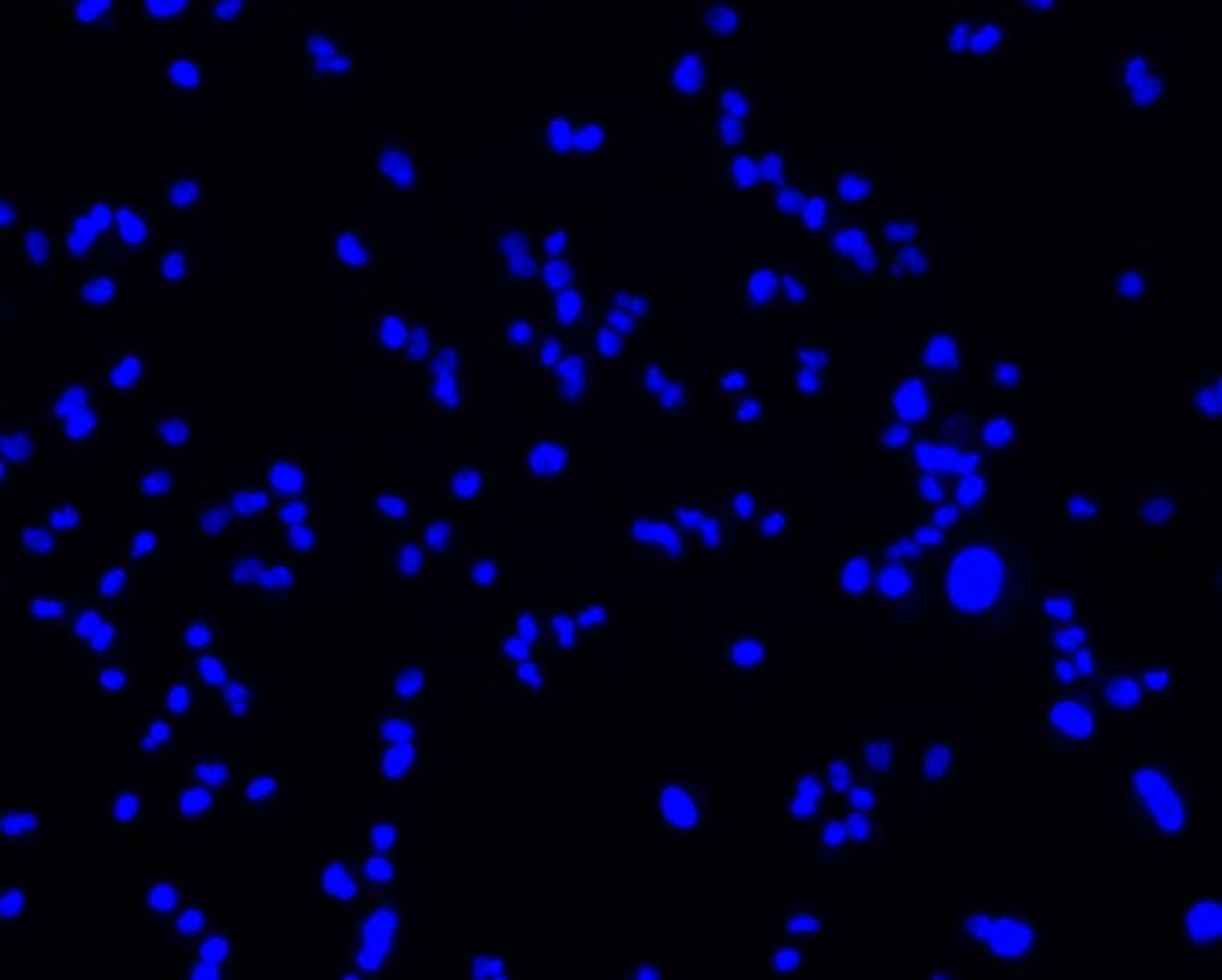

Supplement: Supplementary file 1 — Additional file 1: Supplementary Fig. 1. a In breast cancer cell line and non-tumor cell line MCF-10A, the expression of BC069792 was the highest in non-tumor cell line MCF-10A, while in breast cancer cell line, the expression of BC069792 in MDA-MB-231 and MDA-MB-468 cell lines was the lowest. b CCK-8 experiments showed that si-BC069792 can promote the proliferation of MDA-MB-231 cells (*P=0.43) and MDA-MB-468 (*P=0.026) cells. c The results of EdU experiments showed that si-BC069792 promoted the proliferation ability of breast MDA-MB-231 (*P=0.042) and MDA-MB-468 (*P=0.050) cancer cells. d Compared with the control group, the si-BC069792 knockdown group can effectively promote the migration (P=0.044) and invasion ability (P=0.002) of MDA-MB-231 cells, while the si-BC069792 knockdown group can effectively promote the migration (P=0.002) and invasion (**P=0.005) of MDA-MB-468 cells, and the number of cells passing through the underfloor membrane of the chamber is significantly increased. *P< 0.05, **P< 0.01, ***P< 0.001. Supplementary Fig. 2. Wound healing experiment confirmed that BC069792 can effectively inhibit the migration ability of breast cancer cells. Supplementary Fig. 3. Gene differential expression results after breast cancer cells overexpressed BC069792 a The results of principal component analysis showed that the consistency within the two sample groups was good and had difference. b The results of gene difference analysis showed that the BC069792 overexpression group could cause differential expression of 1209 downstream genes. c The differential expression pathway shown in the figure related to the transduction function of synaptic transmission signal. Supplementary Fig. 4. The exprssion of KCNQ4 protein in the knockdown BC069792 group was significantly reduced (*p=0.014). [file 12943_2023_1747_MOESM1_ESM.zip › Supplementary Materials/Supplementary Figure 1/1c-MDA-MB- 231-NC-DAPI.jpg]

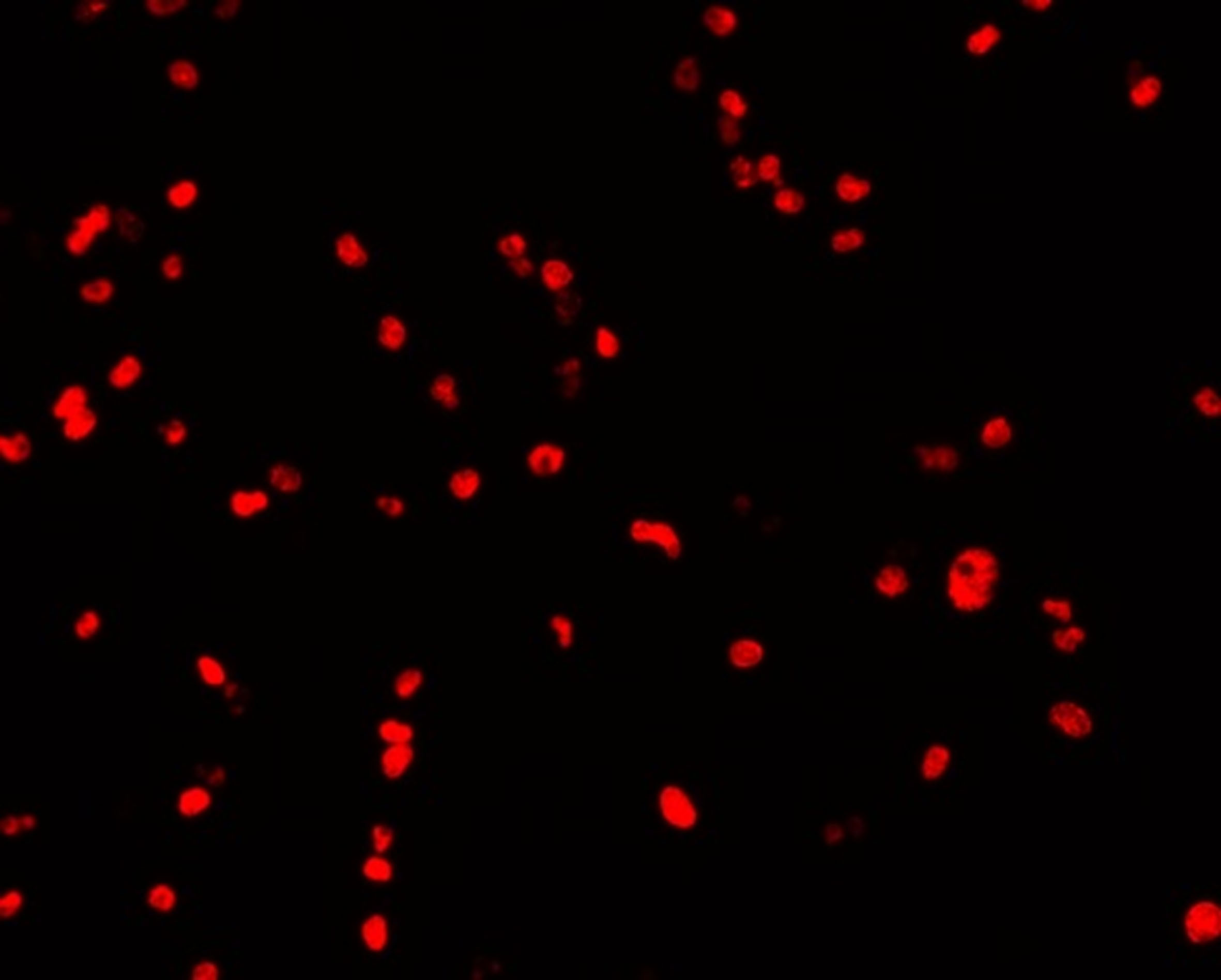

Supplement: Supplementary file 1 — Additional file 1: Supplementary Fig. 1. a In breast cancer cell line and non-tumor cell line MCF-10A, the expression of BC069792 was the highest in non-tumor cell line MCF-10A, while in breast cancer cell line, the expression of BC069792 in MDA-MB-231 and MDA-MB-468 cell lines was the lowest. b CCK-8 experiments showed that si-BC069792 can promote the proliferation of MDA-MB-231 cells (*P=0.43) and MDA-MB-468 (*P=0.026) cells. c The results of EdU experiments showed that si-BC069792 promoted the proliferation ability of breast MDA-MB-231 (*P=0.042) and MDA-MB-468 (*P=0.050) cancer cells. d Compared with the control group, the si-BC069792 knockdown group can effectively promote the migration (P=0.044) and invasion ability (P=0.002) of MDA-MB-231 cells, while the si-BC069792 knockdown group can effectively promote the migration (P=0.002) and invasion (**P=0.005) of MDA-MB-468 cells, and the number of cells passing through the underfloor membrane of the chamber is significantly increased. *P< 0.05, **P< 0.01, ***P< 0.001. Supplementary Fig. 2. Wound healing experiment confirmed that BC069792 can effectively inhibit the migration ability of breast cancer cells. Supplementary Fig. 3. Gene differential expression results after breast cancer cells overexpressed BC069792 a The results of principal component analysis showed that the consistency within the two sample groups was good and had difference. b The results of gene difference analysis showed that the BC069792 overexpression group could cause differential expression of 1209 downstream genes. c The differential expression pathway shown in the figure related to the transduction function of synaptic transmission signal. Supplementary Fig. 4. The exprssion of KCNQ4 protein in the knockdown BC069792 group was significantly reduced (*p=0.014). [file 12943_2023_1747_MOESM1_ESM.zip › Supplementary Materials/Supplementary Figure 1/1c-MDA-MB- 231-NC-EdU.jpg]

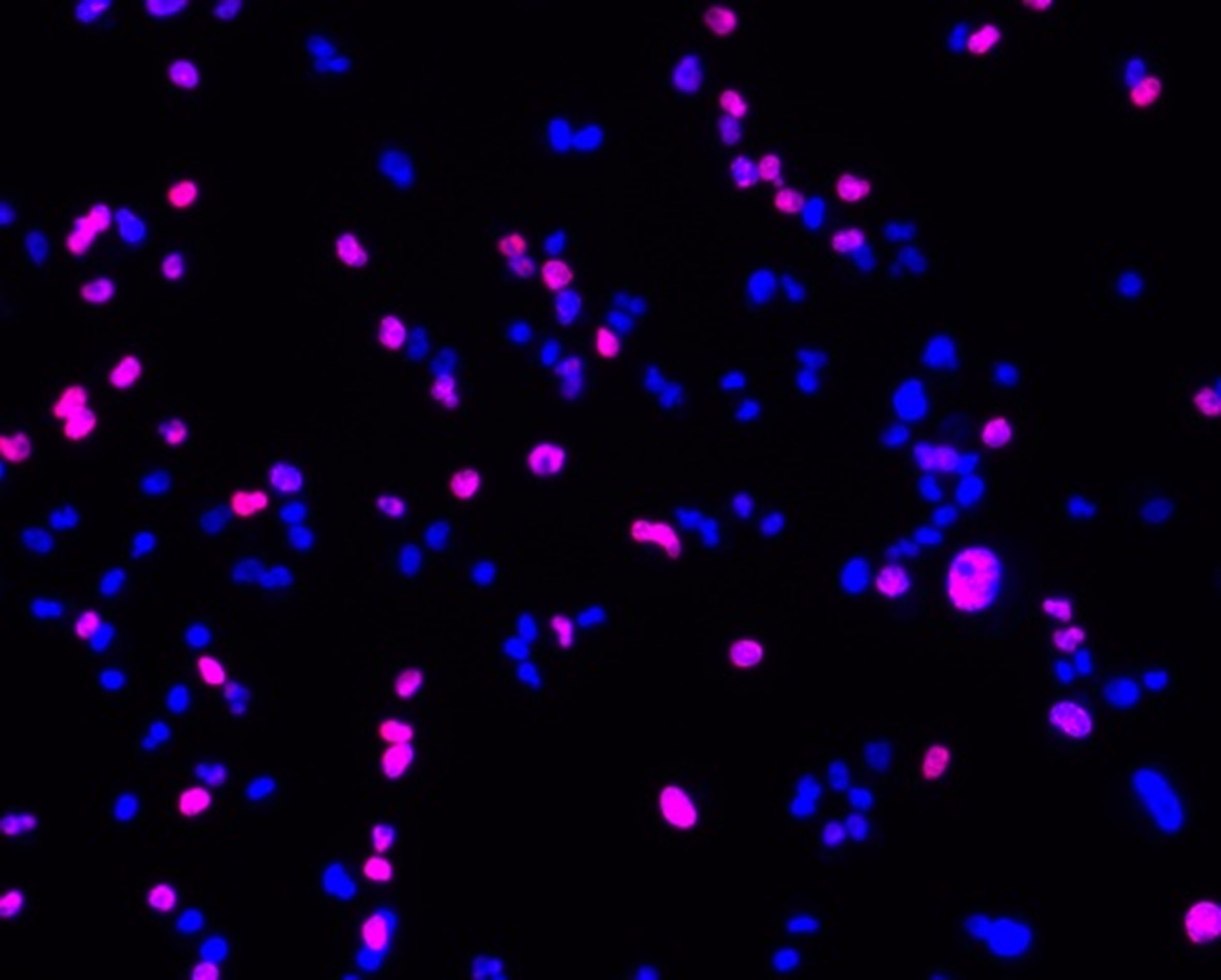

Supplement: Supplementary file 1 — Additional file 1: Supplementary Fig. 1. a In breast cancer cell line and non-tumor cell line MCF-10A, the expression of BC069792 was the highest in non-tumor cell line MCF-10A, while in breast cancer cell line, the expression of BC069792 in MDA-MB-231 and MDA-MB-468 cell lines was the lowest. b CCK-8 experiments showed that si-BC069792 can promote the proliferation of MDA-MB-231 cells (*P=0.43) and MDA-MB-468 (*P=0.026) cells. c The results of EdU experiments showed that si-BC069792 promoted the proliferation ability of breast MDA-MB-231 (*P=0.042) and MDA-MB-468 (*P=0.050) cancer cells. d Compared with the control group, the si-BC069792 knockdown group can effectively promote the migration (P=0.044) and invasion ability (P=0.002) of MDA-MB-231 cells, while the si-BC069792 knockdown group can effectively promote the migration (P=0.002) and invasion (**P=0.005) of MDA-MB-468 cells, and the number of cells passing through the underfloor membrane of the chamber is significantly increased. *P< 0.05, **P< 0.01, ***P< 0.001. Supplementary Fig. 2. Wound healing experiment confirmed that BC069792 can effectively inhibit the migration ability of breast cancer cells. Supplementary Fig. 3. Gene differential expression results after breast cancer cells overexpressed BC069792 a The results of principal component analysis showed that the consistency within the two sample groups was good and had difference. b The results of gene difference analysis showed that the BC069792 overexpression group could cause differential expression of 1209 downstream genes. c The differential expression pathway shown in the figure related to the transduction function of synaptic transmission signal. Supplementary Fig. 4. The exprssion of KCNQ4 protein in the knockdown BC069792 group was significantly reduced (*p=0.014). [file 12943_2023_1747_MOESM1_ESM.zip › Supplementary Materials/Supplementary Figure 1/1c-MDA-MB- 231-NC-Merged.jpg]

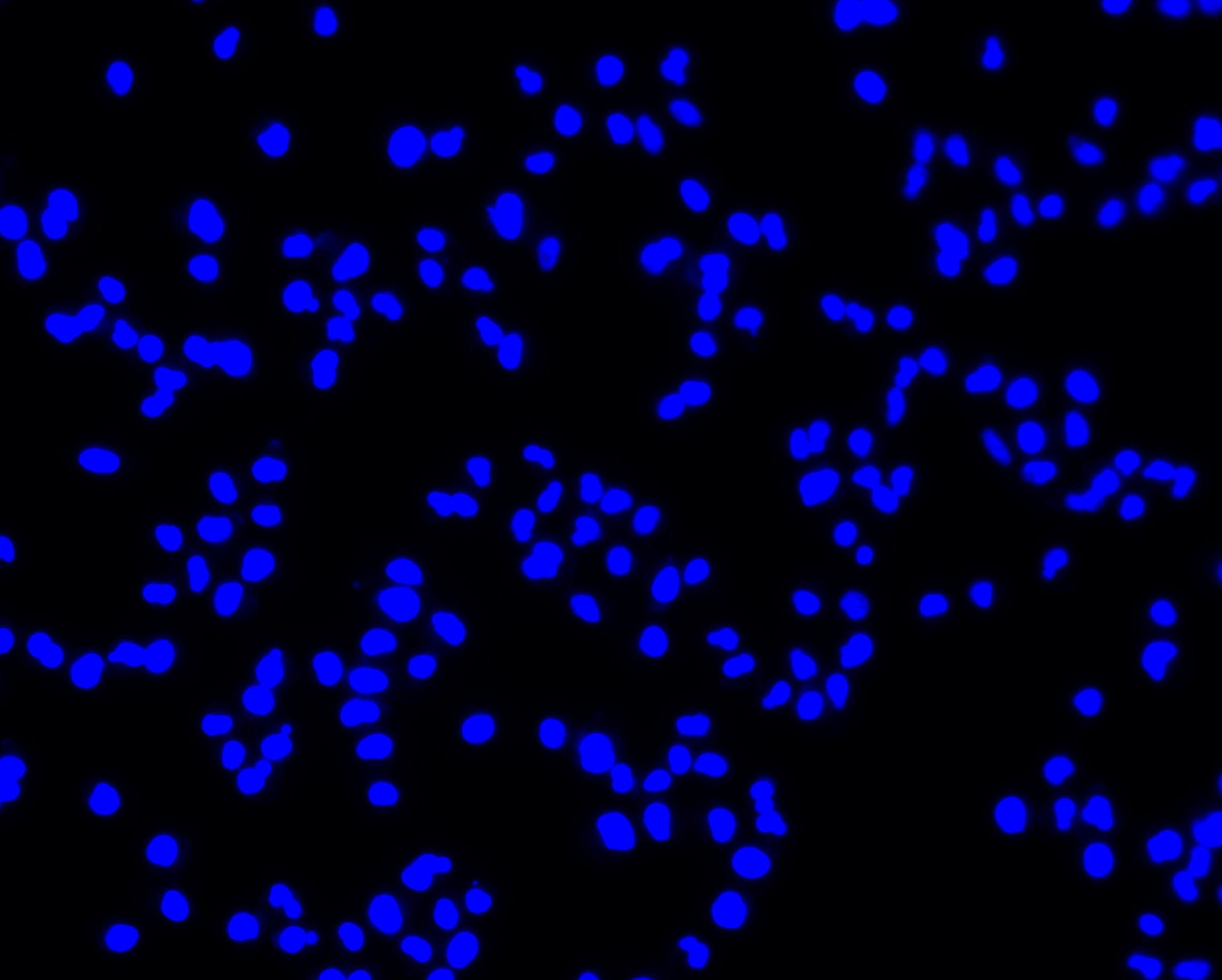

Supplement: Supplementary file 1 — Additional file 1: Supplementary Fig. 1. a In breast cancer cell line and non-tumor cell line MCF-10A, the expression of BC069792 was the highest in non-tumor cell line MCF-10A, while in breast cancer cell line, the expression of BC069792 in MDA-MB-231 and MDA-MB-468 cell lines was the lowest. b CCK-8 experiments showed that si-BC069792 can promote the proliferation of MDA-MB-231 cells (*P=0.43) and MDA-MB-468 (*P=0.026) cells. c The results of EdU experiments showed that si-BC069792 promoted the proliferation ability of breast MDA-MB-231 (*P=0.042) and MDA-MB-468 (*P=0.050) cancer cells. d Compared with the control group, the si-BC069792 knockdown group can effectively promote the migration (P=0.044) and invasion ability (P=0.002) of MDA-MB-231 cells, while the si-BC069792 knockdown group can effectively promote the migration (P=0.002) and invasion (**P=0.005) of MDA-MB-468 cells, and the number of cells passing through the underfloor membrane of the chamber is significantly increased. *P< 0.05, **P< 0.01, ***P< 0.001. Supplementary Fig. 2. Wound healing experiment confirmed that BC069792 can effectively inhibit the migration ability of breast cancer cells. Supplementary Fig. 3. Gene differential expression results after breast cancer cells overexpressed BC069792 a The results of principal component analysis showed that the consistency within the two sample groups was good and had difference. b The results of gene difference analysis showed that the BC069792 overexpression group could cause differential expression of 1209 downstream genes. c The differential expression pathway shown in the figure related to the transduction function of synaptic transmission signal. Supplementary Fig. 4. The exprssion of KCNQ4 protein in the knockdown BC069792 group was significantly reduced (*p=0.014). [file 12943_2023_1747_MOESM1_ESM.zip › Supplementary Materials/Supplementary Figure 1/1c-MDA-MB- 231-si-BC069792-DAPI.jpg]

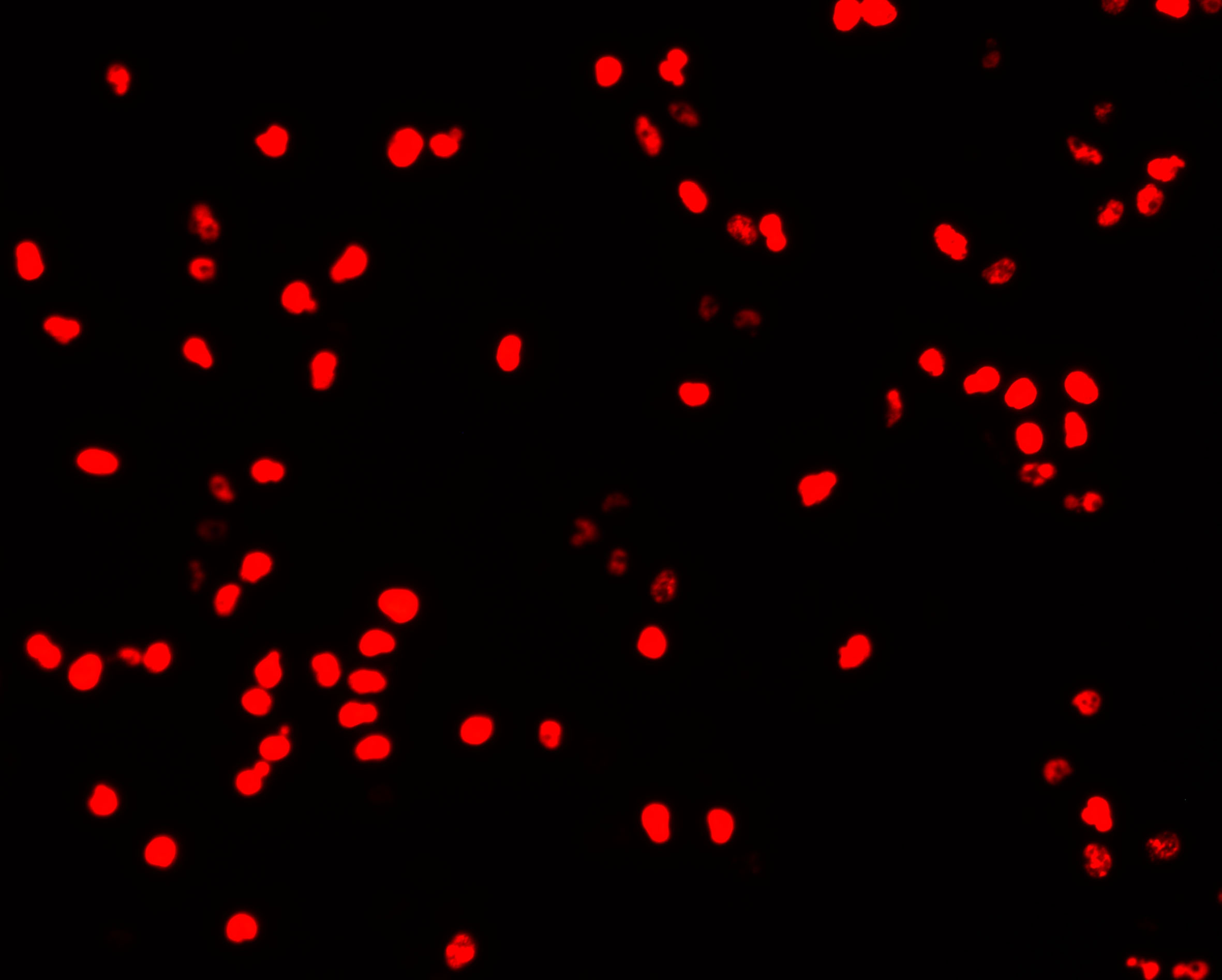

Supplement: Supplementary file 1 — Additional file 1: Supplementary Fig. 1. a In breast cancer cell line and non-tumor cell line MCF-10A, the expression of BC069792 was the highest in non-tumor cell line MCF-10A, while in breast cancer cell line, the expression of BC069792 in MDA-MB-231 and MDA-MB-468 cell lines was the lowest. b CCK-8 experiments showed that si-BC069792 can promote the proliferation of MDA-MB-231 cells (*P=0.43) and MDA-MB-468 (*P=0.026) cells. c The results of EdU experiments showed that si-BC069792 promoted the proliferation ability of breast MDA-MB-231 (*P=0.042) and MDA-MB-468 (*P=0.050) cancer cells. d Compared with the control group, the si-BC069792 knockdown group can effectively promote the migration (P=0.044) and invasion ability (P=0.002) of MDA-MB-231 cells, while the si-BC069792 knockdown group can effectively promote the migration (P=0.002) and invasion (**P=0.005) of MDA-MB-468 cells, and the number of cells passing through the underfloor membrane of the chamber is significantly increased. *P< 0.05, **P< 0.01, ***P< 0.001. Supplementary Fig. 2. Wound healing experiment confirmed that BC069792 can effectively inhibit the migration ability of breast cancer cells. Supplementary Fig. 3. Gene differential expression results after breast cancer cells overexpressed BC069792 a The results of principal component analysis showed that the consistency within the two sample groups was good and had difference. b The results of gene difference analysis showed that the BC069792 overexpression group could cause differential expression of 1209 downstream genes. c The differential expression pathway shown in the figure related to the transduction function of synaptic transmission signal. Supplementary Fig. 4. The exprssion of KCNQ4 protein in the knockdown BC069792 group was significantly reduced (*p=0.014). [file 12943_2023_1747_MOESM1_ESM.zip › Supplementary Materials/Supplementary Figure 1/1c-MDA-MB- 231-si-BC069792-EdU.jpg]

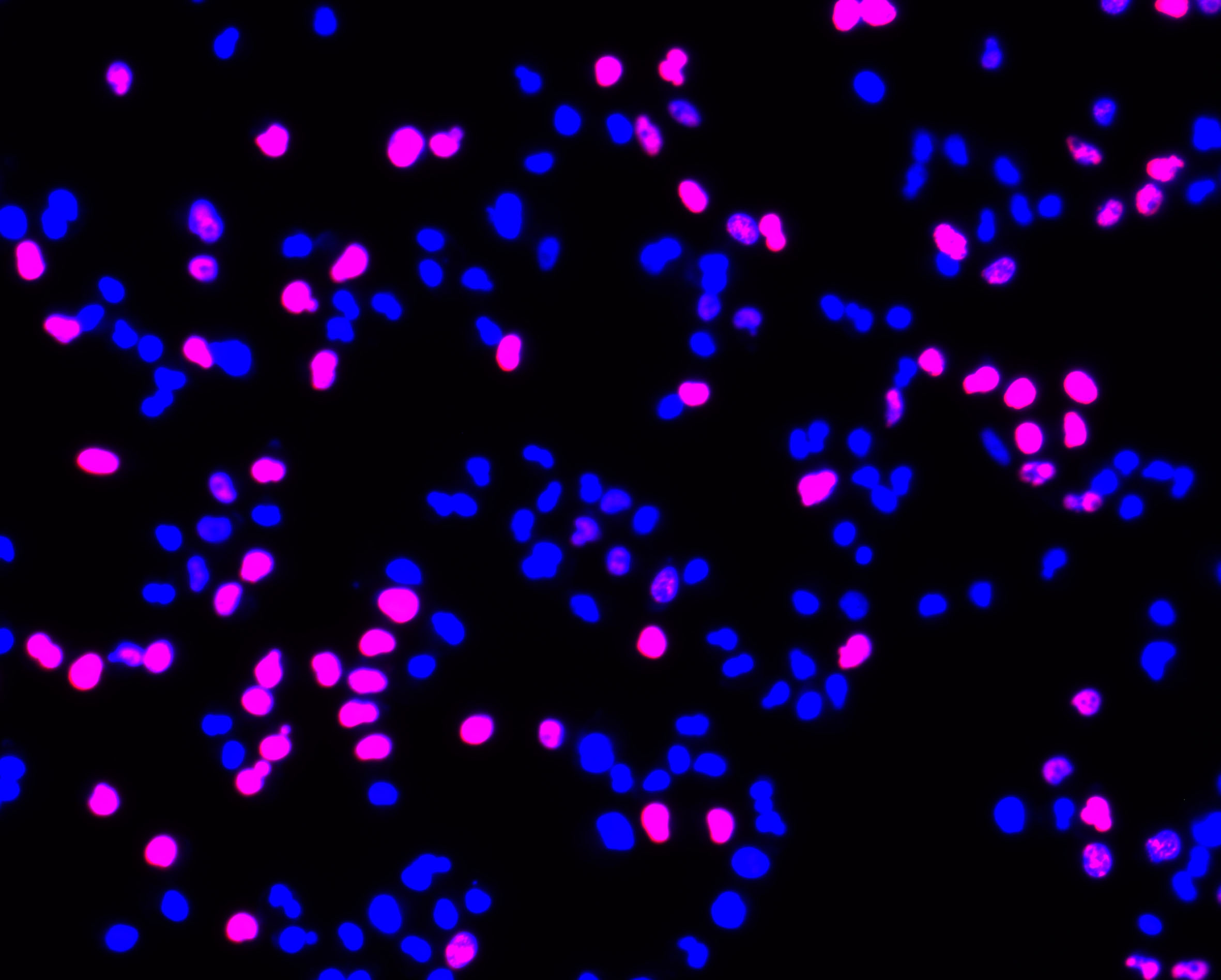

Supplement: Supplementary file 1 — Additional file 1: Supplementary Fig. 1. a In breast cancer cell line and non-tumor cell line MCF-10A, the expression of BC069792 was the highest in non-tumor cell line MCF-10A, while in breast cancer cell line, the expression of BC069792 in MDA-MB-231 and MDA-MB-468 cell lines was the lowest. b CCK-8 experiments showed that si-BC069792 can promote the proliferation of MDA-MB-231 cells (*P=0.43) and MDA-MB-468 (*P=0.026) cells. c The results of EdU experiments showed that si-BC069792 promoted the proliferation ability of breast MDA-MB-231 (*P=0.042) and MDA-MB-468 (*P=0.050) cancer cells. d Compared with the control group, the si-BC069792 knockdown group can effectively promote the migration (P=0.044) and invasion ability (P=0.002) of MDA-MB-231 cells, while the si-BC069792 knockdown group can effectively promote the migration (P=0.002) and invasion (**P=0.005) of MDA-MB-468 cells, and the number of cells passing through the underfloor membrane of the chamber is significantly increased. *P< 0.05, **P< 0.01, ***P< 0.001. Supplementary Fig. 2. Wound healing experiment confirmed that BC069792 can effectively inhibit the migration ability of breast cancer cells. Supplementary Fig. 3. Gene differential expression results after breast cancer cells overexpressed BC069792 a The results of principal component analysis showed that the consistency within the two sample groups was good and had difference. b The results of gene difference analysis showed that the BC069792 overexpression group could cause differential expression of 1209 downstream genes. c The differential expression pathway shown in the figure related to the transduction function of synaptic transmission signal. Supplementary Fig. 4. The exprssion of KCNQ4 protein in the knockdown BC069792 group was significantly reduced (*p=0.014). [file 12943_2023_1747_MOESM1_ESM.zip › Supplementary Materials/Supplementary Figure 1/1c-MDA-MB- 231-si-BC069792-Merged.jpg]

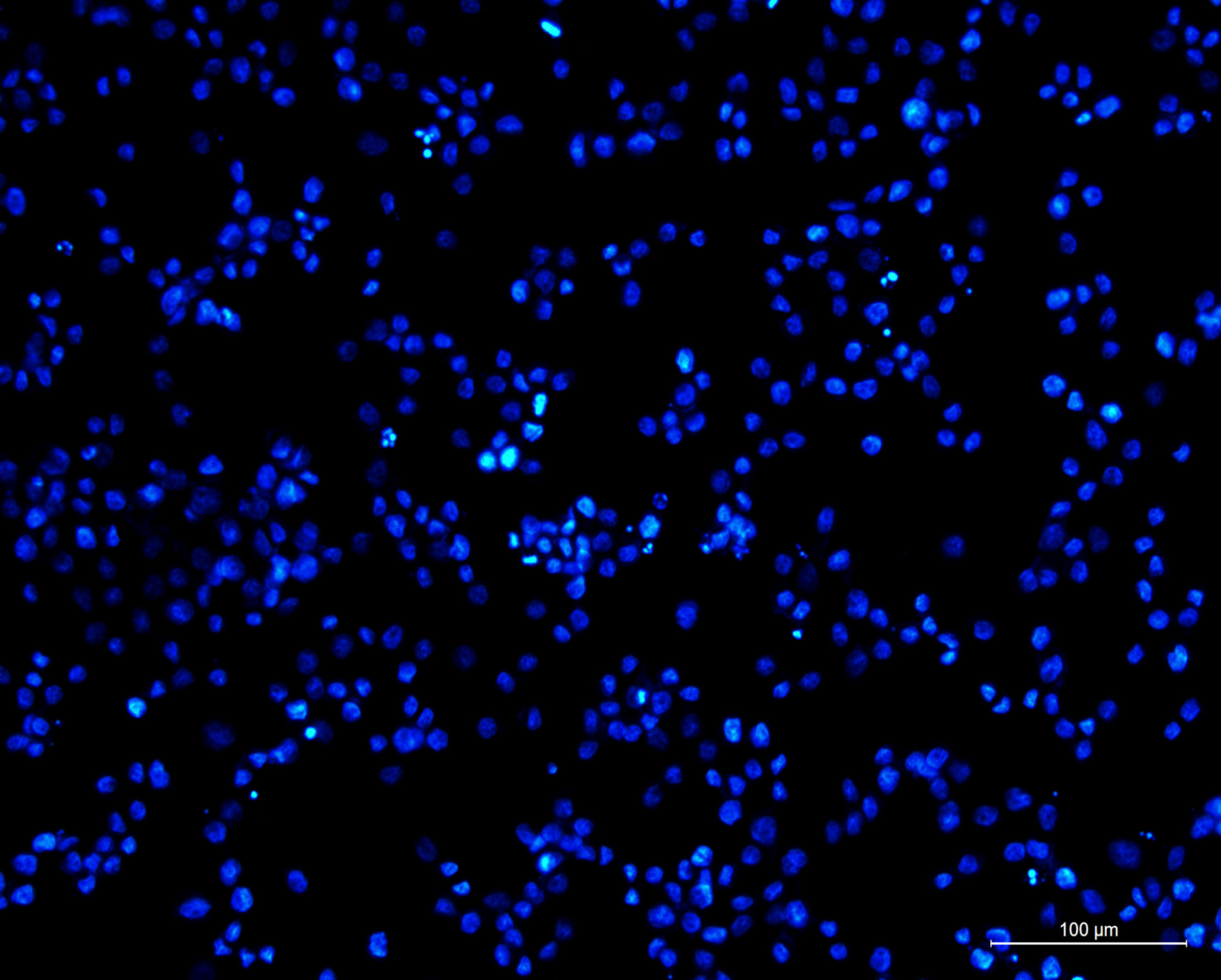

Supplement: Supplementary file 1 — Additional file 1: Supplementary Fig. 1. a In breast cancer cell line and non-tumor cell line MCF-10A, the expression of BC069792 was the highest in non-tumor cell line MCF-10A, while in breast cancer cell line, the expression of BC069792 in MDA-MB-231 and MDA-MB-468 cell lines was the lowest. b CCK-8 experiments showed that si-BC069792 can promote the proliferation of MDA-MB-231 cells (*P=0.43) and MDA-MB-468 (*P=0.026) cells. c The results of EdU experiments showed that si-BC069792 promoted the proliferation ability of breast MDA-MB-231 (*P=0.042) and MDA-MB-468 (*P=0.050) cancer cells. d Compared with the control group, the si-BC069792 knockdown group can effectively promote the migration (P=0.044) and invasion ability (P=0.002) of MDA-MB-231 cells, while the si-BC069792 knockdown group can effectively promote the migration (P=0.002) and invasion (**P=0.005) of MDA-MB-468 cells, and the number of cells passing through the underfloor membrane of the chamber is significantly increased. *P< 0.05, **P< 0.01, ***P< 0.001. Supplementary Fig. 2. Wound healing experiment confirmed that BC069792 can effectively inhibit the migration ability of breast cancer cells. Supplementary Fig. 3. Gene differential expression results after breast cancer cells overexpressed BC069792 a The results of principal component analysis showed that the consistency within the two sample groups was good and had difference. b The results of gene difference analysis showed that the BC069792 overexpression group could cause differential expression of 1209 downstream genes. c The differential expression pathway shown in the figure related to the transduction function of synaptic transmission signal. Supplementary Fig. 4. The exprssion of KCNQ4 protein in the knockdown BC069792 group was significantly reduced (*p=0.014). [file 12943_2023_1747_MOESM1_ESM.zip › Supplementary Materials/Supplementary Figure 1/1c-MDA-MB-468-NC-DAPI.jpg]

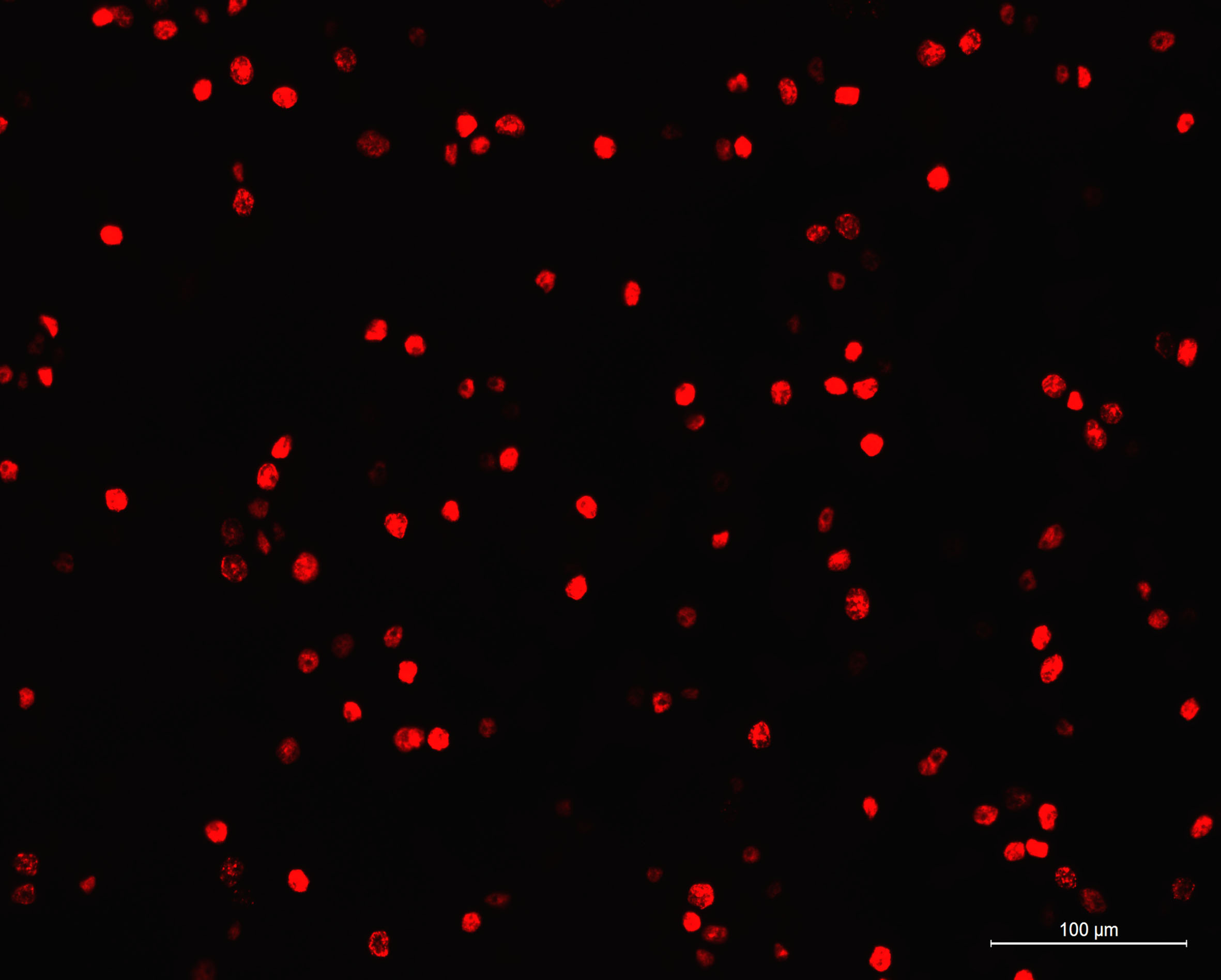

Supplement: Supplementary file 1 — Additional file 1: Supplementary Fig. 1. a In breast cancer cell line and non-tumor cell line MCF-10A, the expression of BC069792 was the highest in non-tumor cell line MCF-10A, while in breast cancer cell line, the expression of BC069792 in MDA-MB-231 and MDA-MB-468 cell lines was the lowest. b CCK-8 experiments showed that si-BC069792 can promote the proliferation of MDA-MB-231 cells (*P=0.43) and MDA-MB-468 (*P=0.026) cells. c The results of EdU experiments showed that si-BC069792 promoted the proliferation ability of breast MDA-MB-231 (*P=0.042) and MDA-MB-468 (*P=0.050) cancer cells. d Compared with the control group, the si-BC069792 knockdown group can effectively promote the migration (P=0.044) and invasion ability (P=0.002) of MDA-MB-231 cells, while the si-BC069792 knockdown group can effectively promote the migration (P=0.002) and invasion (**P=0.005) of MDA-MB-468 cells, and the number of cells passing through the underfloor membrane of the chamber is significantly increased. *P< 0.05, **P< 0.01, ***P< 0.001. Supplementary Fig. 2. Wound healing experiment confirmed that BC069792 can effectively inhibit the migration ability of breast cancer cells. Supplementary Fig. 3. Gene differential expression results after breast cancer cells overexpressed BC069792 a The results of principal component analysis showed that the consistency within the two sample groups was good and had difference. b The results of gene difference analysis showed that the BC069792 overexpression group could cause differential expression of 1209 downstream genes. c The differential expression pathway shown in the figure related to the transduction function of synaptic transmission signal. Supplementary Fig. 4. The exprssion of KCNQ4 protein in the knockdown BC069792 group was significantly reduced (*p=0.014). [file 12943_2023_1747_MOESM1_ESM.zip › Supplementary Materials/Supplementary Figure 1/1c-MDA-MB-468-NC-EdU.jpg]

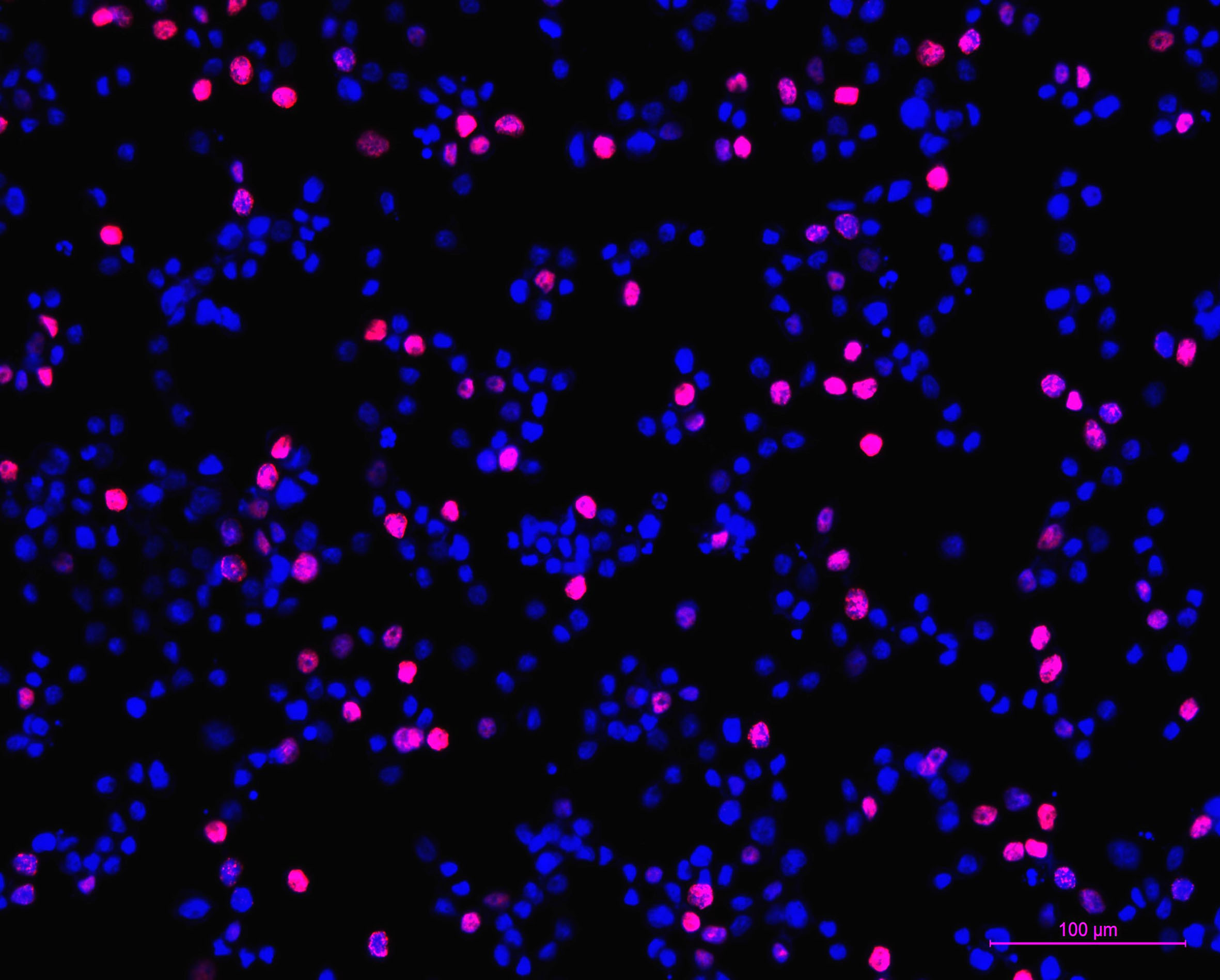

Supplement: Supplementary file 1 — Additional file 1: Supplementary Fig. 1. a In breast cancer cell line and non-tumor cell line MCF-10A, the expression of BC069792 was the highest in non-tumor cell line MCF-10A, while in breast cancer cell line, the expression of BC069792 in MDA-MB-231 and MDA-MB-468 cell lines was the lowest. b CCK-8 experiments showed that si-BC069792 can promote the proliferation of MDA-MB-231 cells (*P=0.43) and MDA-MB-468 (*P=0.026) cells. c The results of EdU experiments showed that si-BC069792 promoted the proliferation ability of breast MDA-MB-231 (*P=0.042) and MDA-MB-468 (*P=0.050) cancer cells. d Compared with the control group, the si-BC069792 knockdown group can effectively promote the migration (P=0.044) and invasion ability (P=0.002) of MDA-MB-231 cells, while the si-BC069792 knockdown group can effectively promote the migration (P=0.002) and invasion (**P=0.005) of MDA-MB-468 cells, and the number of cells passing through the underfloor membrane of the chamber is significantly increased. *P< 0.05, **P< 0.01, ***P< 0.001. Supplementary Fig. 2. Wound healing experiment confirmed that BC069792 can effectively inhibit the migration ability of breast cancer cells. Supplementary Fig. 3. Gene differential expression results after breast cancer cells overexpressed BC069792 a The results of principal component analysis showed that the consistency within the two sample groups was good and had difference. b The results of gene difference analysis showed that the BC069792 overexpression group could cause differential expression of 1209 downstream genes. c The differential expression pathway shown in the figure related to the transduction function of synaptic transmission signal. Supplementary Fig. 4. The exprssion of KCNQ4 protein in the knockdown BC069792 group was significantly reduced (*p=0.014). [file 12943_2023_1747_MOESM1_ESM.zip › Supplementary Materials/Supplementary Figure 1/1c-MDA-MB-468-NC-Merged.jpg]

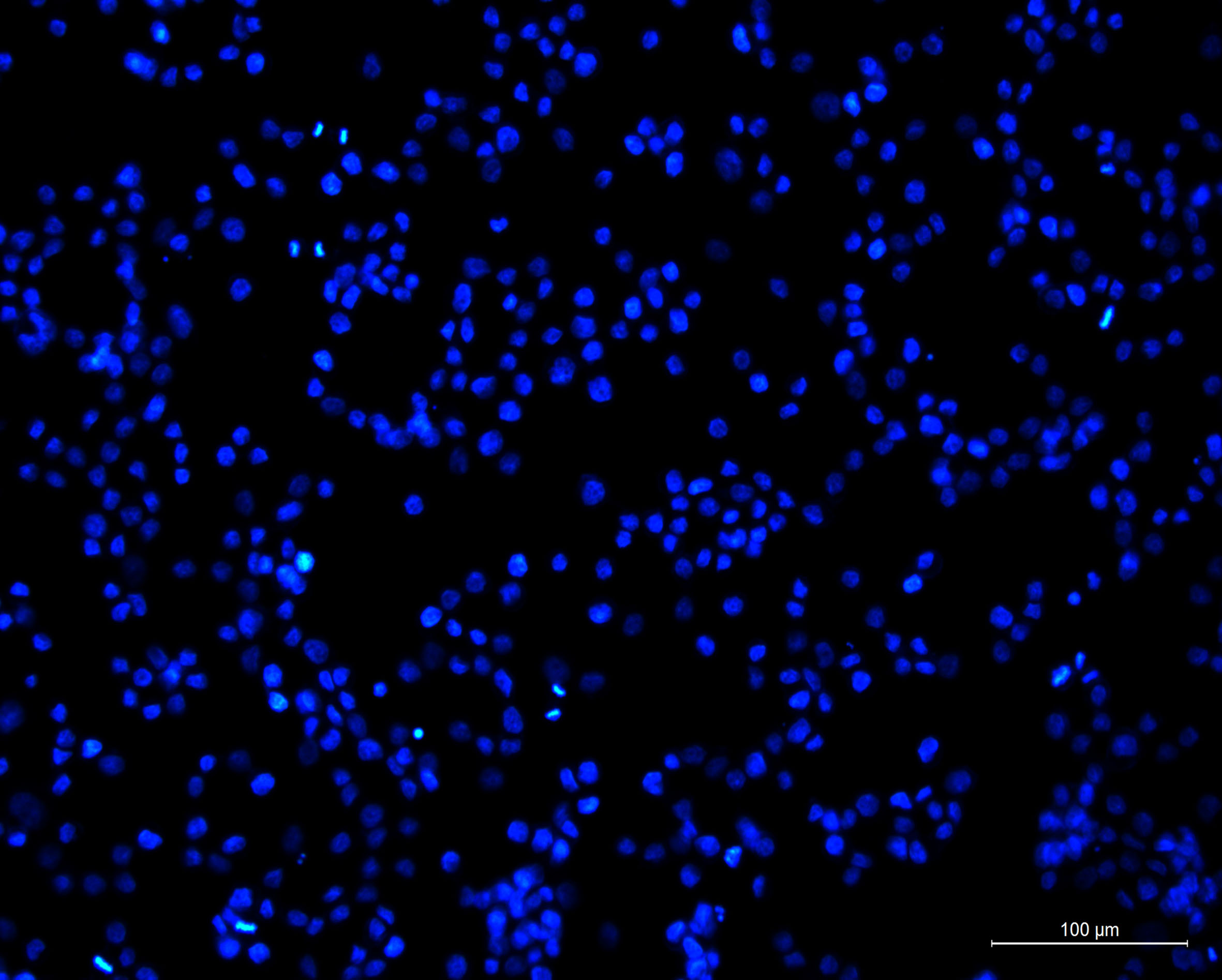

Supplement: Supplementary file 1 — Additional file 1: Supplementary Fig. 1. a In breast cancer cell line and non-tumor cell line MCF-10A, the expression of BC069792 was the highest in non-tumor cell line MCF-10A, while in breast cancer cell line, the expression of BC069792 in MDA-MB-231 and MDA-MB-468 cell lines was the lowest. b CCK-8 experiments showed that si-BC069792 can promote the proliferation of MDA-MB-231 cells (*P=0.43) and MDA-MB-468 (*P=0.026) cells. c The results of EdU experiments showed that si-BC069792 promoted the proliferation ability of breast MDA-MB-231 (*P=0.042) and MDA-MB-468 (*P=0.050) cancer cells. d Compared with the control group, the si-BC069792 knockdown group can effectively promote the migration (P=0.044) and invasion ability (P=0.002) of MDA-MB-231 cells, while the si-BC069792 knockdown group can effectively promote the migration (P=0.002) and invasion (**P=0.005) of MDA-MB-468 cells, and the number of cells passing through the underfloor membrane of the chamber is significantly increased. *P< 0.05, **P< 0.01, ***P< 0.001. Supplementary Fig. 2. Wound healing experiment confirmed that BC069792 can effectively inhibit the migration ability of breast cancer cells. Supplementary Fig. 3. Gene differential expression results after breast cancer cells overexpressed BC069792 a The results of principal component analysis showed that the consistency within the two sample groups was good and had difference. b The results of gene difference analysis showed that the BC069792 overexpression group could cause differential expression of 1209 downstream genes. c The differential expression pathway shown in the figure related to the transduction function of synaptic transmission signal. Supplementary Fig. 4. The exprssion of KCNQ4 protein in the knockdown BC069792 group was significantly reduced (*p=0.014). [file 12943_2023_1747_MOESM1_ESM.zip › Supplementary Materials/Supplementary Figure 1/1c-MDA-MB-468-si-BC069792-DAPI.jpg]

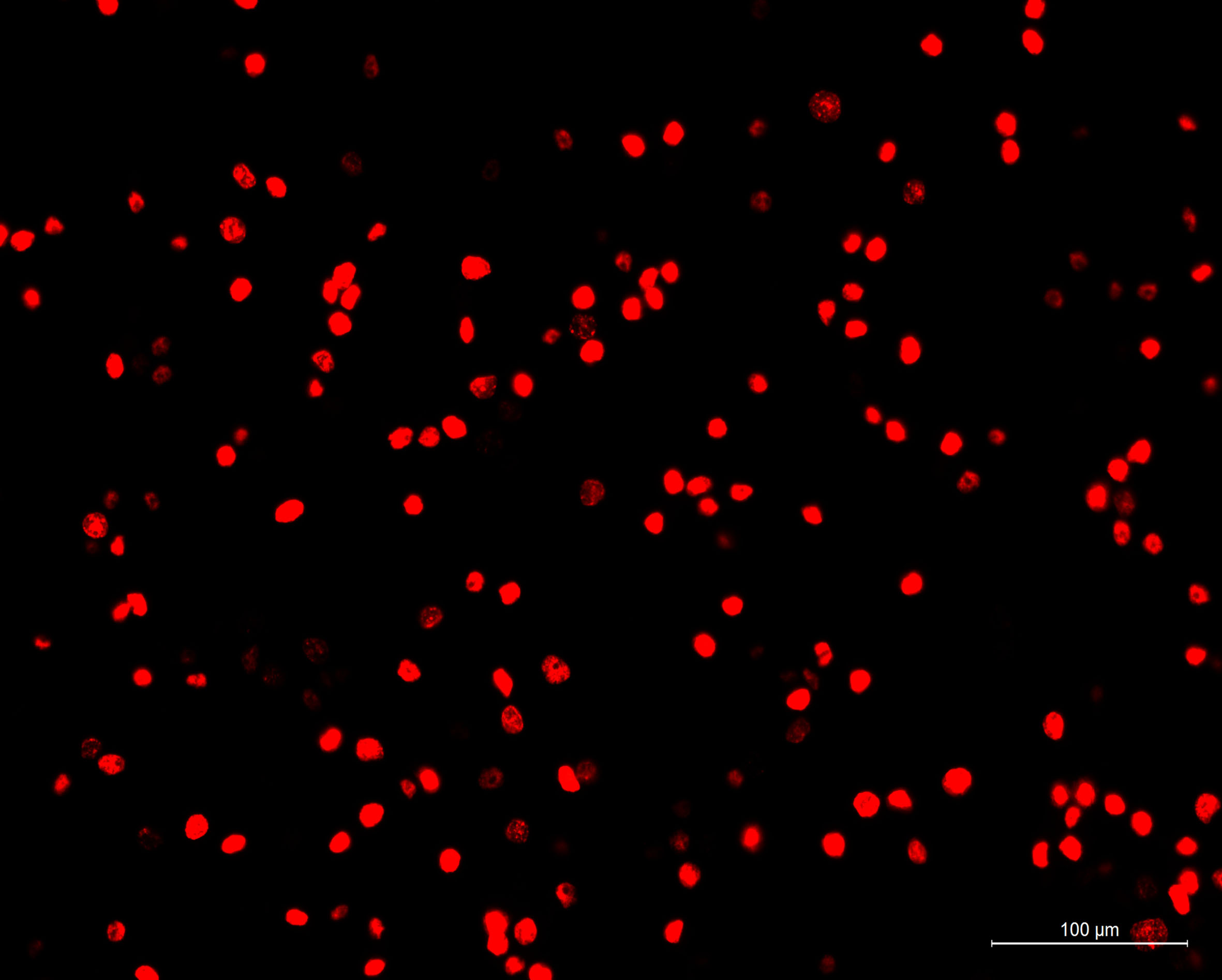

Supplement: Supplementary file 1 — Additional file 1: Supplementary Fig. 1. a In breast cancer cell line and non-tumor cell line MCF-10A, the expression of BC069792 was the highest in non-tumor cell line MCF-10A, while in breast cancer cell line, the expression of BC069792 in MDA-MB-231 and MDA-MB-468 cell lines was the lowest. b CCK-8 experiments showed that si-BC069792 can promote the proliferation of MDA-MB-231 cells (*P=0.43) and MDA-MB-468 (*P=0.026) cells. c The results of EdU experiments showed that si-BC069792 promoted the proliferation ability of breast MDA-MB-231 (*P=0.042) and MDA-MB-468 (*P=0.050) cancer cells. d Compared with the control group, the si-BC069792 knockdown group can effectively promote the migration (P=0.044) and invasion ability (P=0.002) of MDA-MB-231 cells, while the si-BC069792 knockdown group can effectively promote the migration (P=0.002) and invasion (**P=0.005) of MDA-MB-468 cells, and the number of cells passing through the underfloor membrane of the chamber is significantly increased. *P< 0.05, **P< 0.01, ***P< 0.001. Supplementary Fig. 2. Wound healing experiment confirmed that BC069792 can effectively inhibit the migration ability of breast cancer cells. Supplementary Fig. 3. Gene differential expression results after breast cancer cells overexpressed BC069792 a The results of principal component analysis showed that the consistency within the two sample groups was good and had difference. b The results of gene difference analysis showed that the BC069792 overexpression group could cause differential expression of 1209 downstream genes. c The differential expression pathway shown in the figure related to the transduction function of synaptic transmission signal. Supplementary Fig. 4. The exprssion of KCNQ4 protein in the knockdown BC069792 group was significantly reduced (*p=0.014). [file 12943_2023_1747_MOESM1_ESM.zip › Supplementary Materials/Supplementary Figure 1/1c-MDA-MB-468-si-BC069792-EdU.jpg]

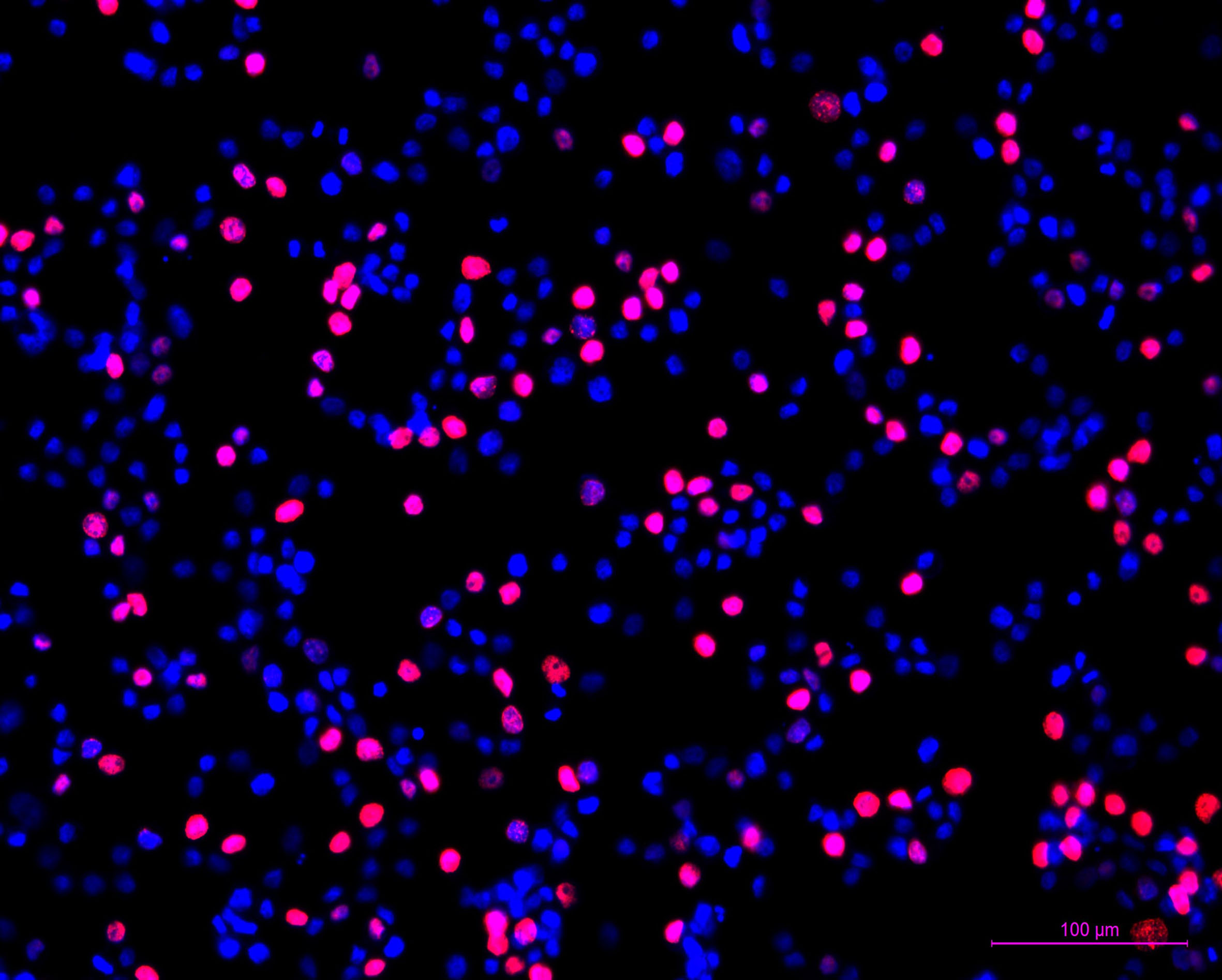

Supplement: Supplementary file 1 — Additional file 1: Supplementary Fig. 1. a In breast cancer cell line and non-tumor cell line MCF-10A, the expression of BC069792 was the highest in non-tumor cell line MCF-10A, while in breast cancer cell line, the expression of BC069792 in MDA-MB-231 and MDA-MB-468 cell lines was the lowest. b CCK-8 experiments showed that si-BC069792 can promote the proliferation of MDA-MB-231 cells (*P=0.43) and MDA-MB-468 (*P=0.026) cells. c The results of EdU experiments showed that si-BC069792 promoted the proliferation ability of breast MDA-MB-231 (*P=0.042) and MDA-MB-468 (*P=0.050) cancer cells. d Compared with the control group, the si-BC069792 knockdown group can effectively promote the migration (P=0.044) and invasion ability (P=0.002) of MDA-MB-231 cells, while the si-BC069792 knockdown group can effectively promote the migration (P=0.002) and invasion (**P=0.005) of MDA-MB-468 cells, and the number of cells passing through the underfloor membrane of the chamber is significantly increased. *P< 0.05, **P< 0.01, ***P< 0.001. Supplementary Fig. 2. Wound healing experiment confirmed that BC069792 can effectively inhibit the migration ability of breast cancer cells. Supplementary Fig. 3. Gene differential expression results after breast cancer cells overexpressed BC069792 a The results of principal component analysis showed that the consistency within the two sample groups was good and had difference. b The results of gene difference analysis showed that the BC069792 overexpression group could cause differential expression of 1209 downstream genes. c The differential expression pathway shown in the figure related to the transduction function of synaptic transmission signal. Supplementary Fig. 4. The exprssion of KCNQ4 protein in the knockdown BC069792 group was significantly reduced (*p=0.014). [file 12943_2023_1747_MOESM1_ESM.zip › Supplementary Materials/Supplementary Figure 1/1c-MDA-MB-468-si-BC069792-Merged.jpg]

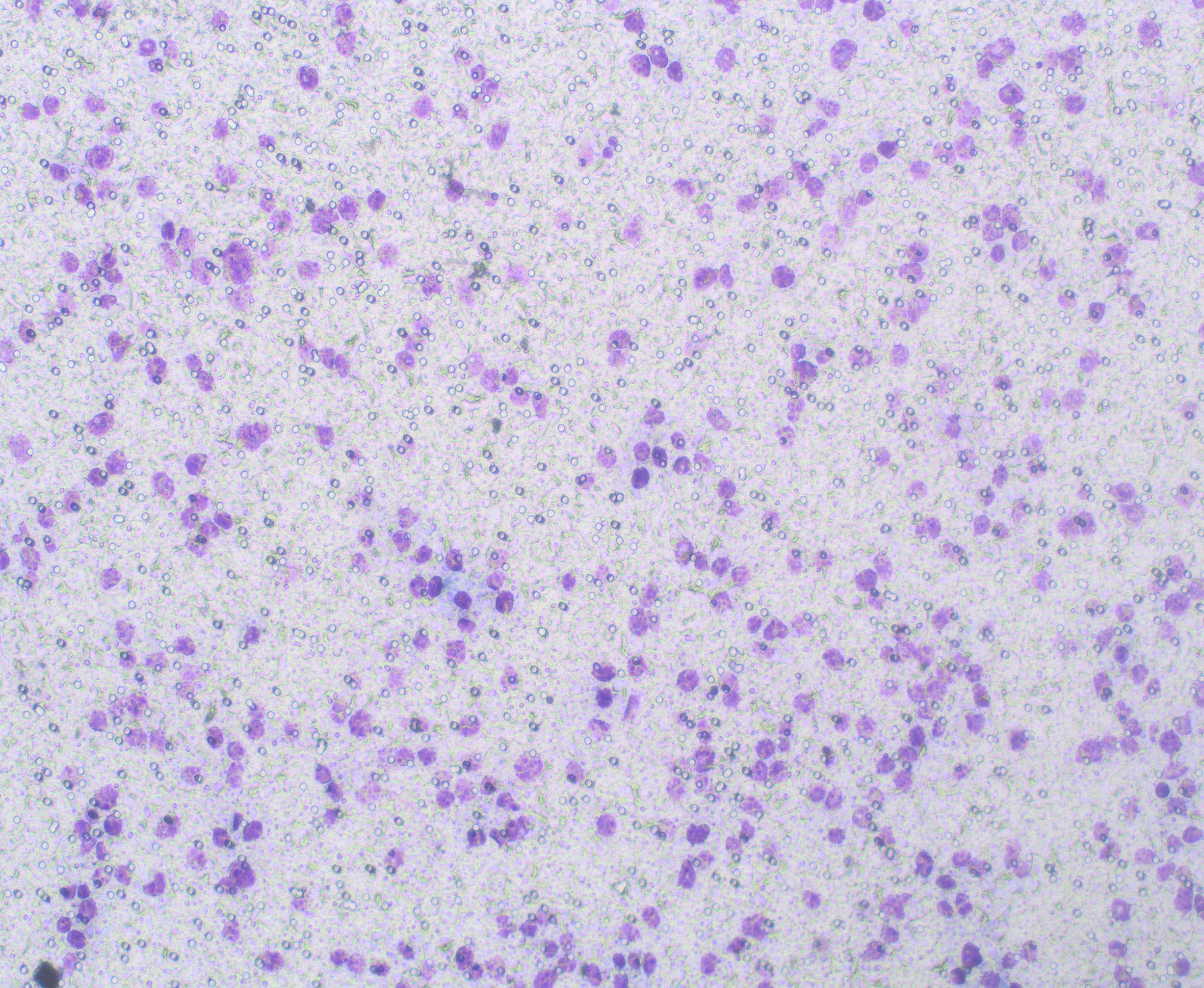

Supplement: Supplementary file 1 — Additional file 1: Supplementary Fig. 1. a In breast cancer cell line and non-tumor cell line MCF-10A, the expression of BC069792 was the highest in non-tumor cell line MCF-10A, while in breast cancer cell line, the expression of BC069792 in MDA-MB-231 and MDA-MB-468 cell lines was the lowest. b CCK-8 experiments showed that si-BC069792 can promote the proliferation of MDA-MB-231 cells (*P=0.43) and MDA-MB-468 (*P=0.026) cells. c The results of EdU experiments showed that si-BC069792 promoted the proliferation ability of breast MDA-MB-231 (*P=0.042) and MDA-MB-468 (*P=0.050) cancer cells. d Compared with the control group, the si-BC069792 knockdown group can effectively promote the migration (P=0.044) and invasion ability (P=0.002) of MDA-MB-231 cells, while the si-BC069792 knockdown group can effectively promote the migration (P=0.002) and invasion (**P=0.005) of MDA-MB-468 cells, and the number of cells passing through the underfloor membrane of the chamber is significantly increased. *P< 0.05, **P< 0.01, ***P< 0.001. Supplementary Fig. 2. Wound healing experiment confirmed that BC069792 can effectively inhibit the migration ability of breast cancer cells. Supplementary Fig. 3. Gene differential expression results after breast cancer cells overexpressed BC069792 a The results of principal component analysis showed that the consistency within the two sample groups was good and had difference. b The results of gene difference analysis showed that the BC069792 overexpression group could cause differential expression of 1209 downstream genes. c The differential expression pathway shown in the figure related to the transduction function of synaptic transmission signal. Supplementary Fig. 4. The exprssion of KCNQ4 protein in the knockdown BC069792 group was significantly reduced (*p=0.014). [file 12943_2023_1747_MOESM1_ESM.zip › Supplementary Materials/Supplementary Figure 1/1d-MDA-MB-231-Invasion-NC.jpg]

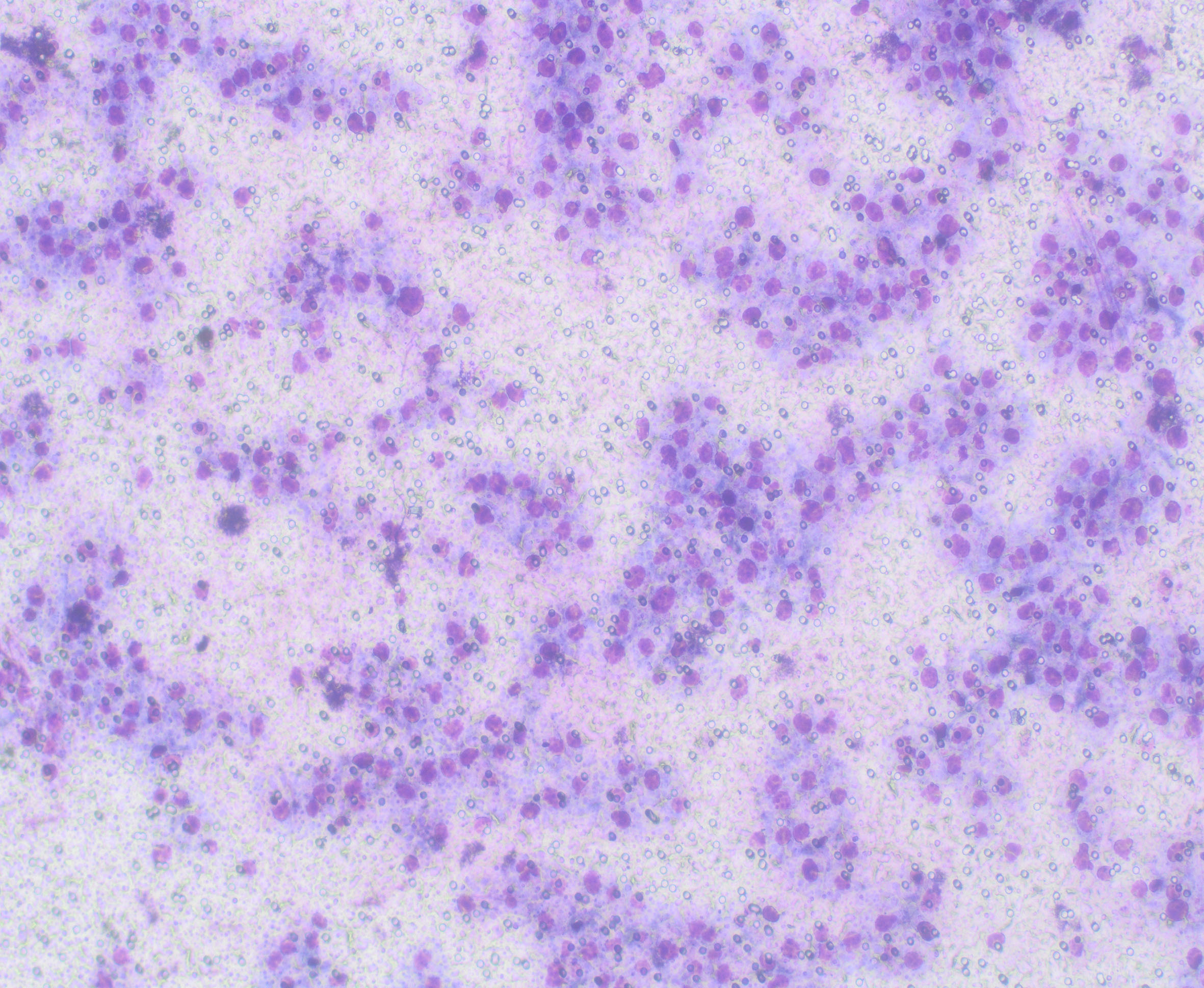

Supplement: Supplementary file 1 — Additional file 1: Supplementary Fig. 1. a In breast cancer cell line and non-tumor cell line MCF-10A, the expression of BC069792 was the highest in non-tumor cell line MCF-10A, while in breast cancer cell line, the expression of BC069792 in MDA-MB-231 and MDA-MB-468 cell lines was the lowest. b CCK-8 experiments showed that si-BC069792 can promote the proliferation of MDA-MB-231 cells (*P=0.43) and MDA-MB-468 (*P=0.026) cells. c The results of EdU experiments showed that si-BC069792 promoted the proliferation ability of breast MDA-MB-231 (*P=0.042) and MDA-MB-468 (*P=0.050) cancer cells. d Compared with the control group, the si-BC069792 knockdown group can effectively promote the migration (P=0.044) and invasion ability (P=0.002) of MDA-MB-231 cells, while the si-BC069792 knockdown group can effectively promote the migration (P=0.002) and invasion (**P=0.005) of MDA-MB-468 cells, and the number of cells passing through the underfloor membrane of the chamber is significantly increased. *P< 0.05, **P< 0.01, ***P< 0.001. Supplementary Fig. 2. Wound healing experiment confirmed that BC069792 can effectively inhibit the migration ability of breast cancer cells. Supplementary Fig. 3. Gene differential expression results after breast cancer cells overexpressed BC069792 a The results of principal component analysis showed that the consistency within the two sample groups was good and had difference. b The results of gene difference analysis showed that the BC069792 overexpression group could cause differential expression of 1209 downstream genes. c The differential expression pathway shown in the figure related to the transduction function of synaptic transmission signal. Supplementary Fig. 4. The exprssion of KCNQ4 protein in the knockdown BC069792 group was significantly reduced (*p=0.014). [file 12943_2023_1747_MOESM1_ESM.zip › Supplementary Materials/Supplementary Figure 1/1d-MDA-MB-231-Invasion-si-BC069792.jpg]

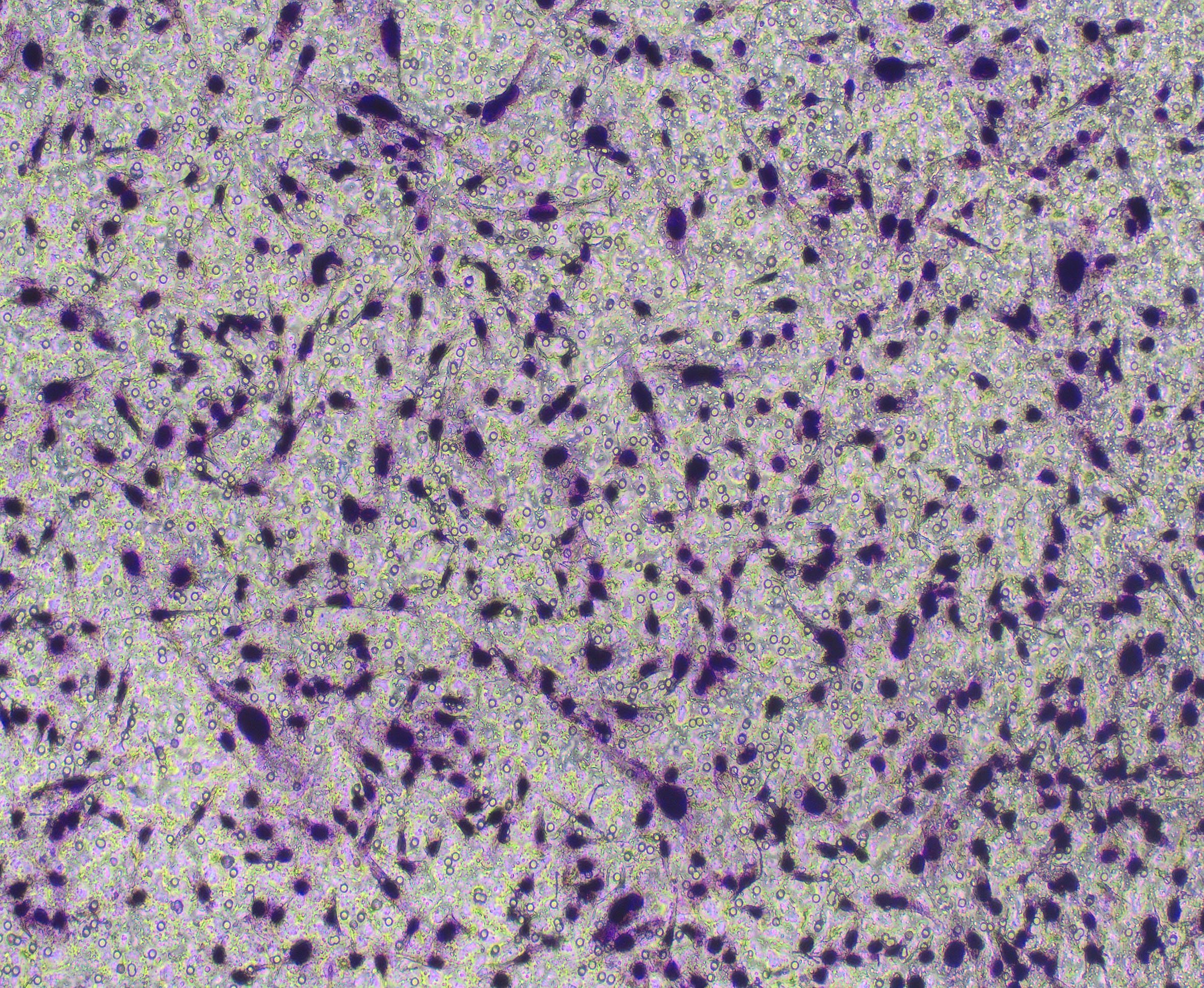

Supplement: Supplementary file 1 — Additional file 1: Supplementary Fig. 1. a In breast cancer cell line and non-tumor cell line MCF-10A, the expression of BC069792 was the highest in non-tumor cell line MCF-10A, while in breast cancer cell line, the expression of BC069792 in MDA-MB-231 and MDA-MB-468 cell lines was the lowest. b CCK-8 experiments showed that si-BC069792 can promote the proliferation of MDA-MB-231 cells (*P=0.43) and MDA-MB-468 (*P=0.026) cells. c The results of EdU experiments showed that si-BC069792 promoted the proliferation ability of breast MDA-MB-231 (*P=0.042) and MDA-MB-468 (*P=0.050) cancer cells. d Compared with the control group, the si-BC069792 knockdown group can effectively promote the migration (P=0.044) and invasion ability (P=0.002) of MDA-MB-231 cells, while the si-BC069792 knockdown group can effectively promote the migration (P=0.002) and invasion (**P=0.005) of MDA-MB-468 cells, and the number of cells passing through the underfloor membrane of the chamber is significantly increased. *P< 0.05, **P< 0.01, ***P< 0.001. Supplementary Fig. 2. Wound healing experiment confirmed that BC069792 can effectively inhibit the migration ability of breast cancer cells. Supplementary Fig. 3. Gene differential expression results after breast cancer cells overexpressed BC069792 a The results of principal component analysis showed that the consistency within the two sample groups was good and had difference. b The results of gene difference analysis showed that the BC069792 overexpression group could cause differential expression of 1209 downstream genes. c The differential expression pathway shown in the figure related to the transduction function of synaptic transmission signal. Supplementary Fig. 4. The exprssion of KCNQ4 protein in the knockdown BC069792 group was significantly reduced (*p=0.014). [file 12943_2023_1747_MOESM1_ESM.zip › Supplementary Materials/Supplementary Figure 1/1d-MDA-MB-231-Migration-NC.jpg]

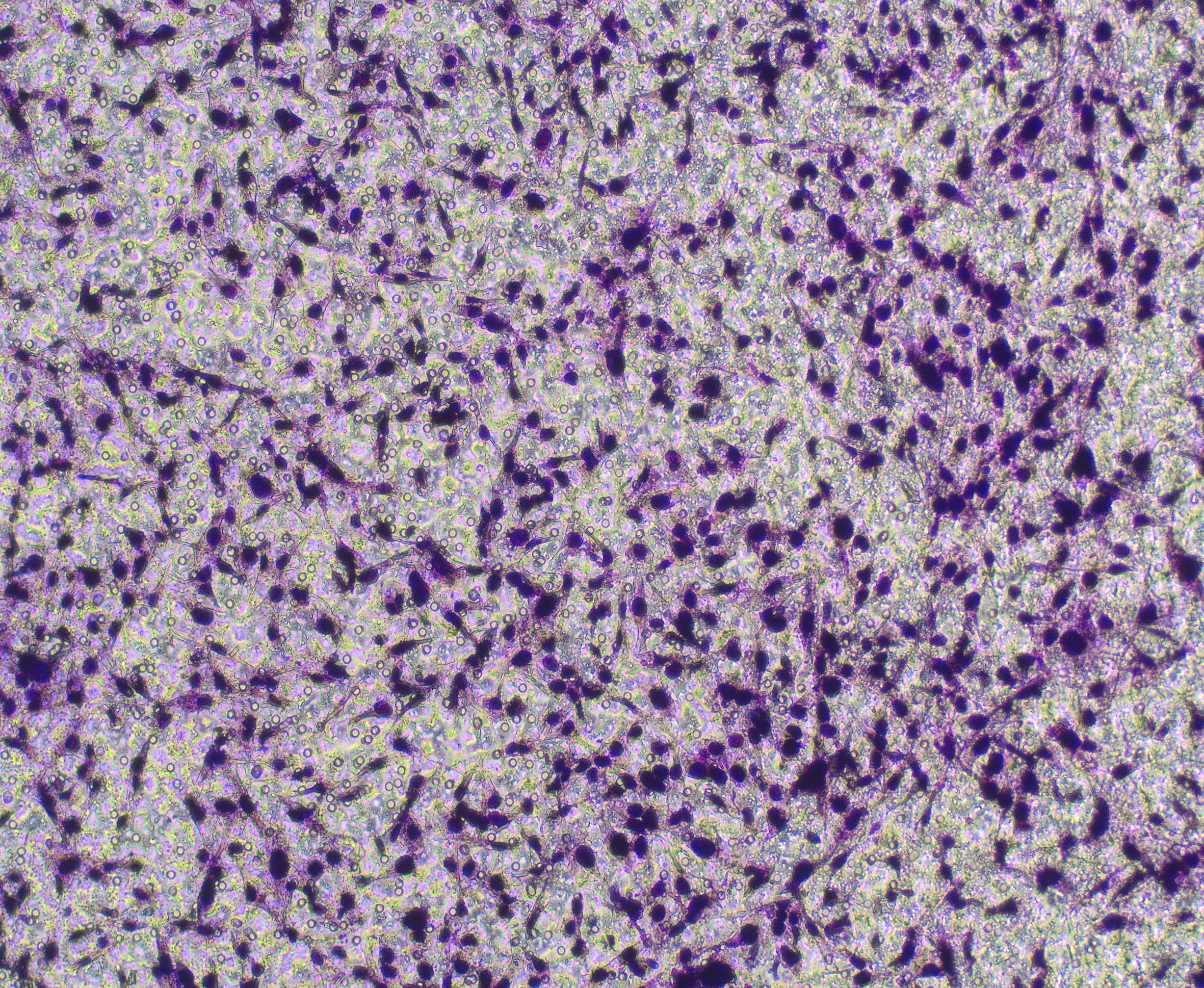

Supplement: Supplementary file 1 — Additional file 1: Supplementary Fig. 1. a In breast cancer cell line and non-tumor cell line MCF-10A, the expression of BC069792 was the highest in non-tumor cell line MCF-10A, while in breast cancer cell line, the expression of BC069792 in MDA-MB-231 and MDA-MB-468 cell lines was the lowest. b CCK-8 experiments showed that si-BC069792 can promote the proliferation of MDA-MB-231 cells (*P=0.43) and MDA-MB-468 (*P=0.026) cells. c The results of EdU experiments showed that si-BC069792 promoted the proliferation ability of breast MDA-MB-231 (*P=0.042) and MDA-MB-468 (*P=0.050) cancer cells. d Compared with the control group, the si-BC069792 knockdown group can effectively promote the migration (P=0.044) and invasion ability (P=0.002) of MDA-MB-231 cells, while the si-BC069792 knockdown group can effectively promote the migration (P=0.002) and invasion (**P=0.005) of MDA-MB-468 cells, and the number of cells passing through the underfloor membrane of the chamber is significantly increased. *P< 0.05, **P< 0.01, ***P< 0.001. Supplementary Fig. 2. Wound healing experiment confirmed that BC069792 can effectively inhibit the migration ability of breast cancer cells. Supplementary Fig. 3. Gene differential expression results after breast cancer cells overexpressed BC069792 a The results of principal component analysis showed that the consistency within the two sample groups was good and had difference. b The results of gene difference analysis showed that the BC069792 overexpression group could cause differential expression of 1209 downstream genes. c The differential expression pathway shown in the figure related to the transduction function of synaptic transmission signal. Supplementary Fig. 4. The exprssion of KCNQ4 protein in the knockdown BC069792 group was significantly reduced (*p=0.014). [file 12943_2023_1747_MOESM1_ESM.zip › Supplementary Materials/Supplementary Figure 1/1d-MDA-MB-231-Migration-si-BC069792.jpg]

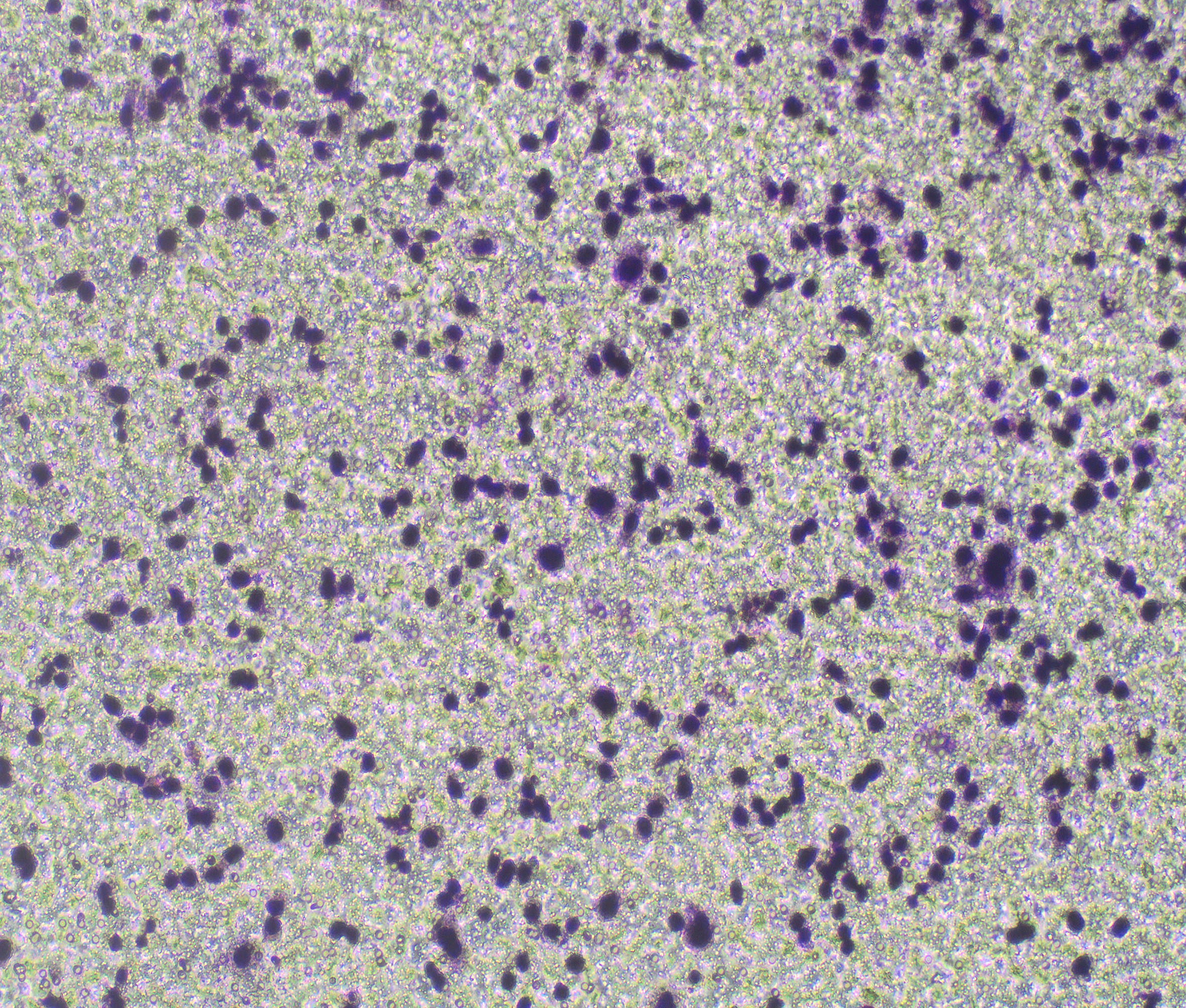

Supplement: Supplementary file 1 — Additional file 1: Supplementary Fig. 1. a In breast cancer cell line and non-tumor cell line MCF-10A, the expression of BC069792 was the highest in non-tumor cell line MCF-10A, while in breast cancer cell line, the expression of BC069792 in MDA-MB-231 and MDA-MB-468 cell lines was the lowest. b CCK-8 experiments showed that si-BC069792 can promote the proliferation of MDA-MB-231 cells (*P=0.43) and MDA-MB-468 (*P=0.026) cells. c The results of EdU experiments showed that si-BC069792 promoted the proliferation ability of breast MDA-MB-231 (*P=0.042) and MDA-MB-468 (*P=0.050) cancer cells. d Compared with the control group, the si-BC069792 knockdown group can effectively promote the migration (P=0.044) and invasion ability (P=0.002) of MDA-MB-231 cells, while the si-BC069792 knockdown group can effectively promote the migration (P=0.002) and invasion (**P=0.005) of MDA-MB-468 cells, and the number of cells passing through the underfloor membrane of the chamber is significantly increased. *P< 0.05, **P< 0.01, ***P< 0.001. Supplementary Fig. 2. Wound healing experiment confirmed that BC069792 can effectively inhibit the migration ability of breast cancer cells. Supplementary Fig. 3. Gene differential expression results after breast cancer cells overexpressed BC069792 a The results of principal component analysis showed that the consistency within the two sample groups was good and had difference. b The results of gene difference analysis showed that the BC069792 overexpression group could cause differential expression of 1209 downstream genes. c The differential expression pathway shown in the figure related to the transduction function of synaptic transmission signal. Supplementary Fig. 4. The exprssion of KCNQ4 protein in the knockdown BC069792 group was significantly reduced (*p=0.014). [file 12943_2023_1747_MOESM1_ESM.zip › Supplementary Materials/Supplementary Figure 1/1d-MDA-MB-468-Invasion-NC.jpg]

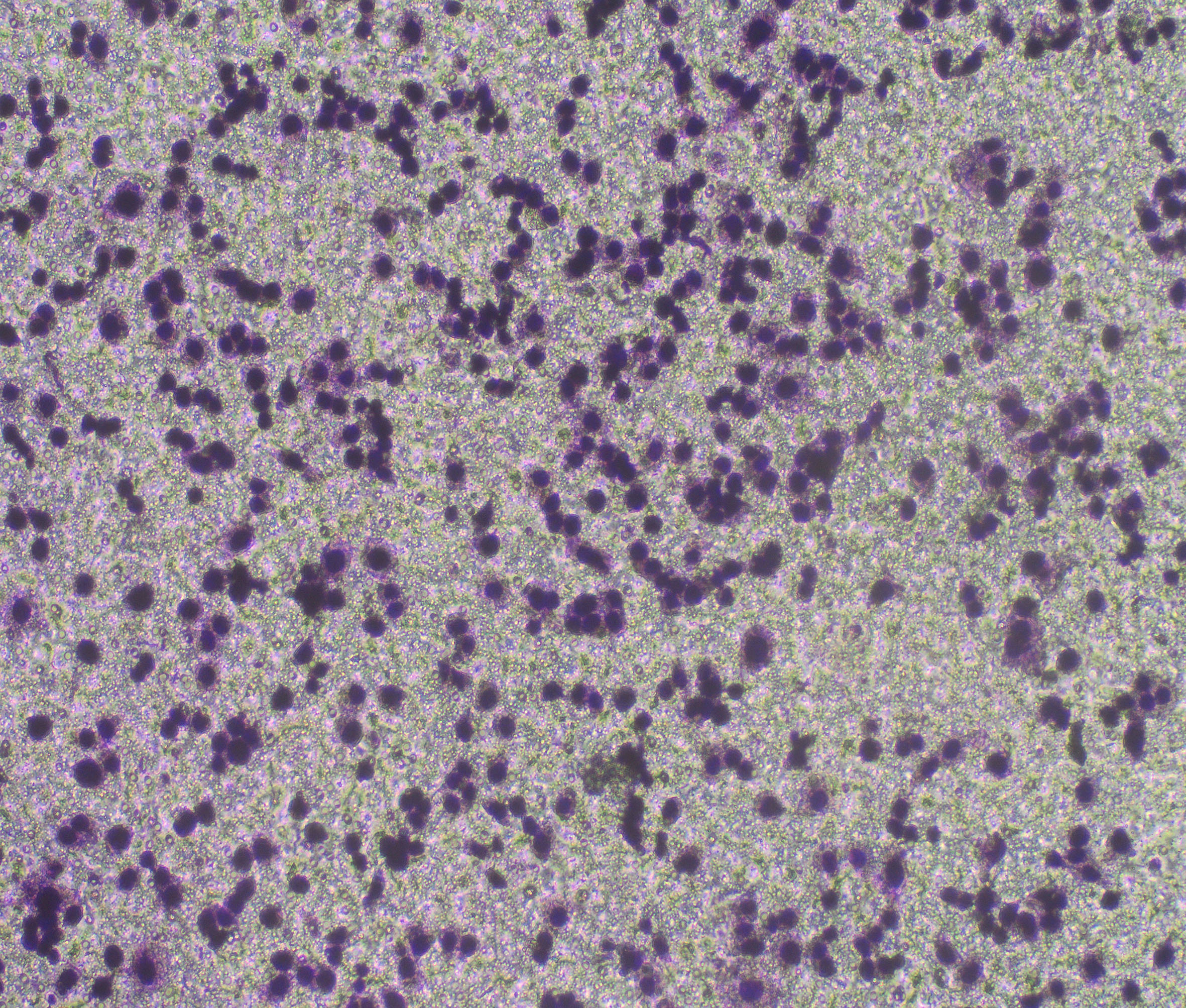

Supplement: Supplementary file 1 — Additional file 1: Supplementary Fig. 1. a In breast cancer cell line and non-tumor cell line MCF-10A, the expression of BC069792 was the highest in non-tumor cell line MCF-10A, while in breast cancer cell line, the expression of BC069792 in MDA-MB-231 and MDA-MB-468 cell lines was the lowest. b CCK-8 experiments showed that si-BC069792 can promote the proliferation of MDA-MB-231 cells (*P=0.43) and MDA-MB-468 (*P=0.026) cells. c The results of EdU experiments showed that si-BC069792 promoted the proliferation ability of breast MDA-MB-231 (*P=0.042) and MDA-MB-468 (*P=0.050) cancer cells. d Compared with the control group, the si-BC069792 knockdown group can effectively promote the migration (P=0.044) and invasion ability (P=0.002) of MDA-MB-231 cells, while the si-BC069792 knockdown group can effectively promote the migration (P=0.002) and invasion (**P=0.005) of MDA-MB-468 cells, and the number of cells passing through the underfloor membrane of the chamber is significantly increased. *P< 0.05, **P< 0.01, ***P< 0.001. Supplementary Fig. 2. Wound healing experiment confirmed that BC069792 can effectively inhibit the migration ability of breast cancer cells. Supplementary Fig. 3. Gene differential expression results after breast cancer cells overexpressed BC069792 a The results of principal component analysis showed that the consistency within the two sample groups was good and had difference. b The results of gene difference analysis showed that the BC069792 overexpression group could cause differential expression of 1209 downstream genes. c The differential expression pathway shown in the figure related to the transduction function of synaptic transmission signal. Supplementary Fig. 4. The exprssion of KCNQ4 protein in the knockdown BC069792 group was significantly reduced (*p=0.014). [file 12943_2023_1747_MOESM1_ESM.zip › Supplementary Materials/Supplementary Figure 1/1d-MDA-MB-468-Invasion-si-BC069792.jpg]

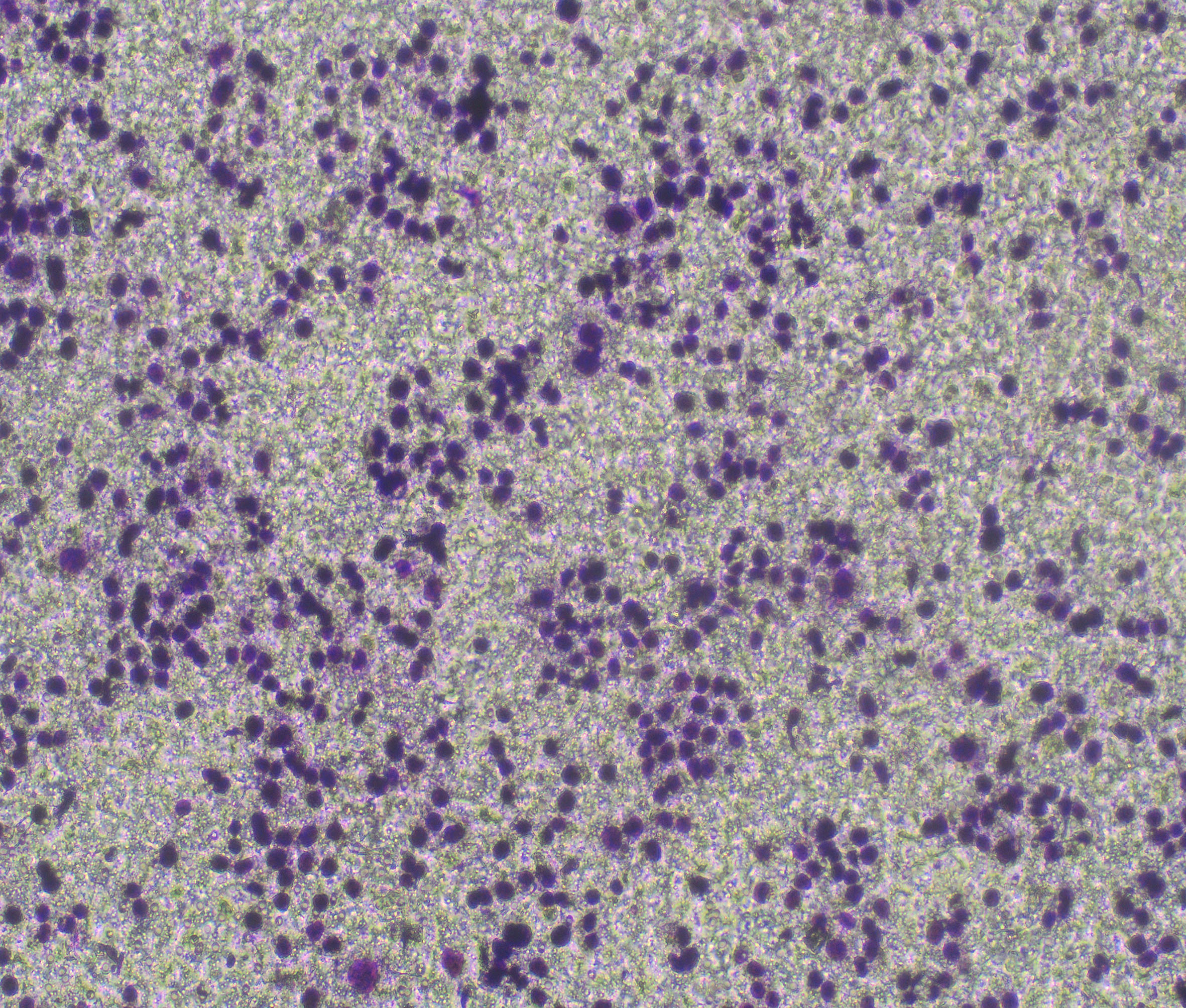

Supplement: Supplementary file 1 — Additional file 1: Supplementary Fig. 1. a In breast cancer cell line and non-tumor cell line MCF-10A, the expression of BC069792 was the highest in non-tumor cell line MCF-10A, while in breast cancer cell line, the expression of BC069792 in MDA-MB-231 and MDA-MB-468 cell lines was the lowest. b CCK-8 experiments showed that si-BC069792 can promote the proliferation of MDA-MB-231 cells (*P=0.43) and MDA-MB-468 (*P=0.026) cells. c The results of EdU experiments showed that si-BC069792 promoted the proliferation ability of breast MDA-MB-231 (*P=0.042) and MDA-MB-468 (*P=0.050) cancer cells. d Compared with the control group, the si-BC069792 knockdown group can effectively promote the migration (P=0.044) and invasion ability (P=0.002) of MDA-MB-231 cells, while the si-BC069792 knockdown group can effectively promote the migration (P=0.002) and invasion (**P=0.005) of MDA-MB-468 cells, and the number of cells passing through the underfloor membrane of the chamber is significantly increased. *P< 0.05, **P< 0.01, ***P< 0.001. Supplementary Fig. 2. Wound healing experiment confirmed that BC069792 can effectively inhibit the migration ability of breast cancer cells. Supplementary Fig. 3. Gene differential expression results after breast cancer cells overexpressed BC069792 a The results of principal component analysis showed that the consistency within the two sample groups was good and had difference. b The results of gene difference analysis showed that the BC069792 overexpression group could cause differential expression of 1209 downstream genes. c The differential expression pathway shown in the figure related to the transduction function of synaptic transmission signal. Supplementary Fig. 4. The exprssion of KCNQ4 protein in the knockdown BC069792 group was significantly reduced (*p=0.014). [file 12943_2023_1747_MOESM1_ESM.zip › Supplementary Materials/Supplementary Figure 1/1d-MDA-MB-468-Migration-NC.jpg]

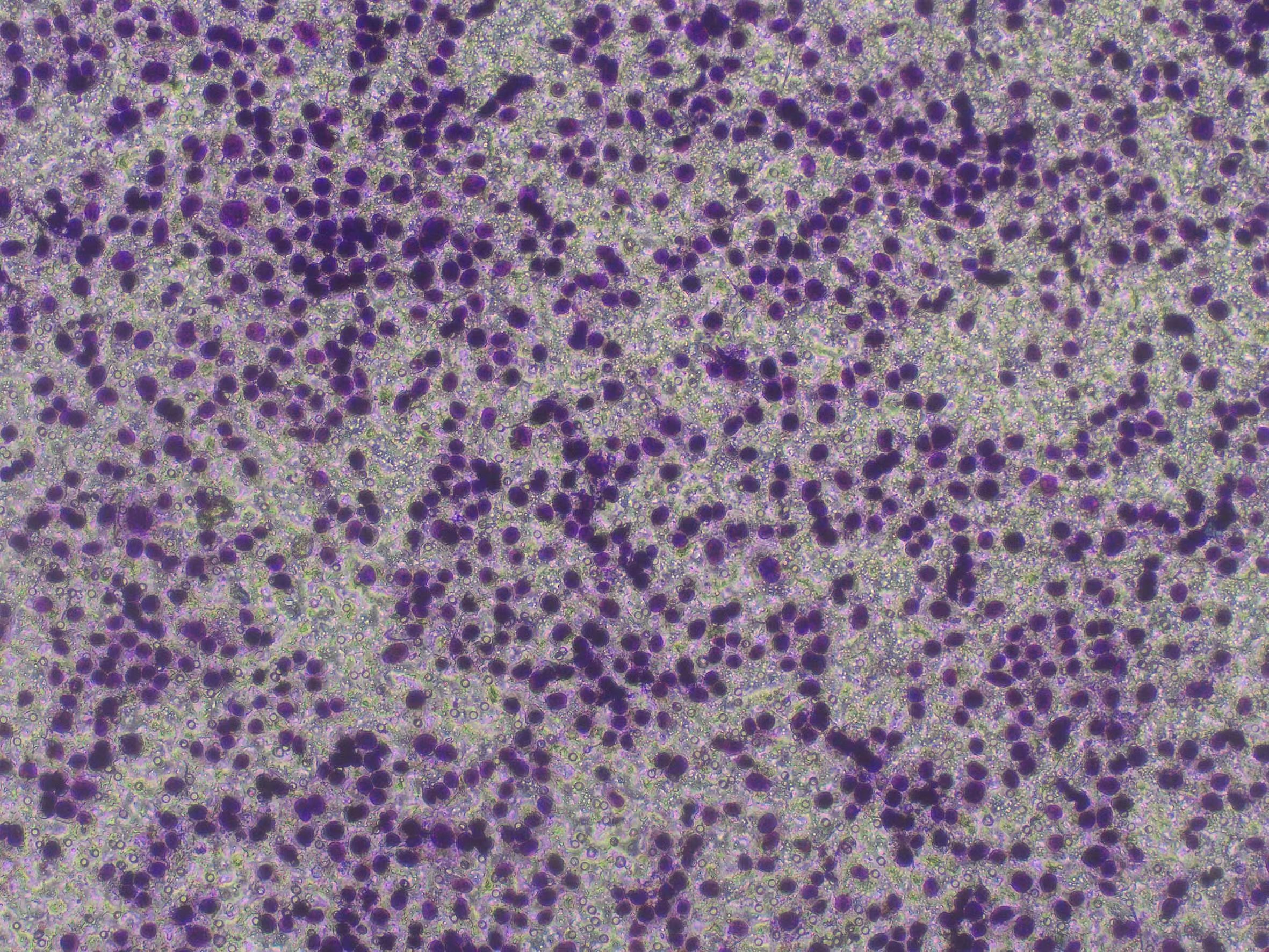

Supplement: Supplementary file 1 — Additional file 1: Supplementary Fig. 1. a In breast cancer cell line and non-tumor cell line MCF-10A, the expression of BC069792 was the highest in non-tumor cell line MCF-10A, while in breast cancer cell line, the expression of BC069792 in MDA-MB-231 and MDA-MB-468 cell lines was the lowest. b CCK-8 experiments showed that si-BC069792 can promote the proliferation of MDA-MB-231 cells (*P=0.43) and MDA-MB-468 (*P=0.026) cells. c The results of EdU experiments showed that si-BC069792 promoted the proliferation ability of breast MDA-MB-231 (*P=0.042) and MDA-MB-468 (*P=0.050) cancer cells. d Compared with the control group, the si-BC069792 knockdown group can effectively promote the migration (P=0.044) and invasion ability (P=0.002) of MDA-MB-231 cells, while the si-BC069792 knockdown group can effectively promote the migration (P=0.002) and invasion (**P=0.005) of MDA-MB-468 cells, and the number of cells passing through the underfloor membrane of the chamber is significantly increased. *P< 0.05, **P< 0.01, ***P< 0.001. Supplementary Fig. 2. Wound healing experiment confirmed that BC069792 can effectively inhibit the migration ability of breast cancer cells. Supplementary Fig. 3. Gene differential expression results after breast cancer cells overexpressed BC069792 a The results of principal component analysis showed that the consistency within the two sample groups was good and had difference. b The results of gene difference analysis showed that the BC069792 overexpression group could cause differential expression of 1209 downstream genes. c The differential expression pathway shown in the figure related to the transduction function of synaptic transmission signal. Supplementary Fig. 4. The exprssion of KCNQ4 protein in the knockdown BC069792 group was significantly reduced (*p=0.014). [file 12943_2023_1747_MOESM1_ESM.zip › Supplementary Materials/Supplementary Figure 1/1d-MDA-MB-468-Migration-si-BC069792.jpg]

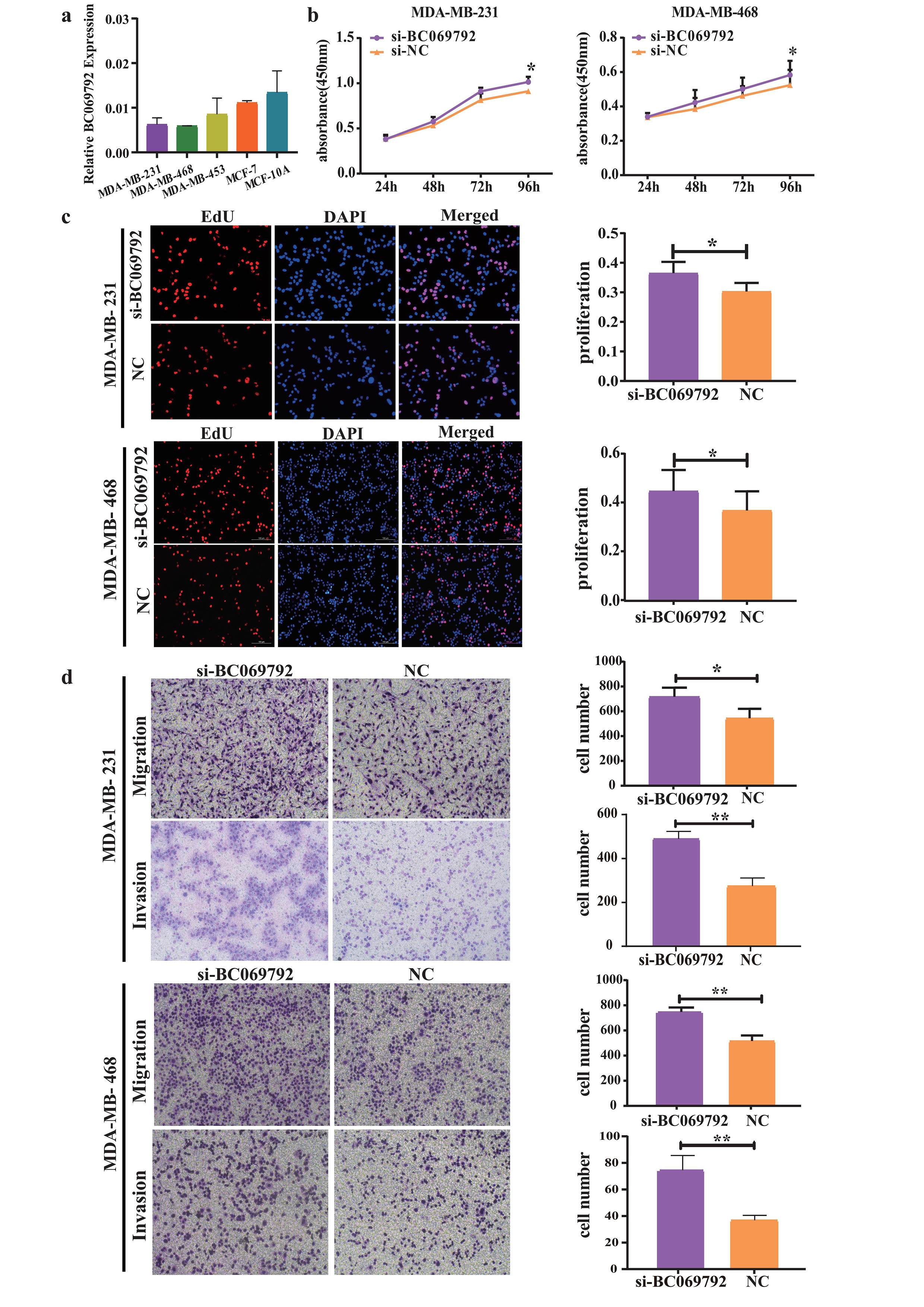

Supplement: Supplementary file 1 — Additional file 1: Supplementary Fig. 1. a In breast cancer cell line and non-tumor cell line MCF-10A, the expression of BC069792 was the highest in non-tumor cell line MCF-10A, while in breast cancer cell line, the expression of BC069792 in MDA-MB-231 and MDA-MB-468 cell lines was the lowest. b CCK-8 experiments showed that si-BC069792 can promote the proliferation of MDA-MB-231 cells (*P=0.43) and MDA-MB-468 (*P=0.026) cells. c The results of EdU experiments showed that si-BC069792 promoted the proliferation ability of breast MDA-MB-231 (*P=0.042) and MDA-MB-468 (*P=0.050) cancer cells. d Compared with the control group, the si-BC069792 knockdown group can effectively promote the migration (P=0.044) and invasion ability (P=0.002) of MDA-MB-231 cells, while the si-BC069792 knockdown group can effectively promote the migration (P=0.002) and invasion (**P=0.005) of MDA-MB-468 cells, and the number of cells passing through the underfloor membrane of the chamber is significantly increased. *P< 0.05, **P< 0.01, ***P< 0.001. Supplementary Fig. 2. Wound healing experiment confirmed that BC069792 can effectively inhibit the migration ability of breast cancer cells. Supplementary Fig. 3. Gene differential expression results after breast cancer cells overexpressed BC069792 a The results of principal component analysis showed that the consistency within the two sample groups was good and had difference. b The results of gene difference analysis showed that the BC069792 overexpression group could cause differential expression of 1209 downstream genes. c The differential expression pathway shown in the figure related to the transduction function of synaptic transmission signal. Supplementary Fig. 4. The exprssion of KCNQ4 protein in the knockdown BC069792 group was significantly reduced (*p=0.014). [file 12943_2023_1747_MOESM1_ESM.zip › Supplementary Materials/Supplementary Figure 1/sup. 1.jpg]

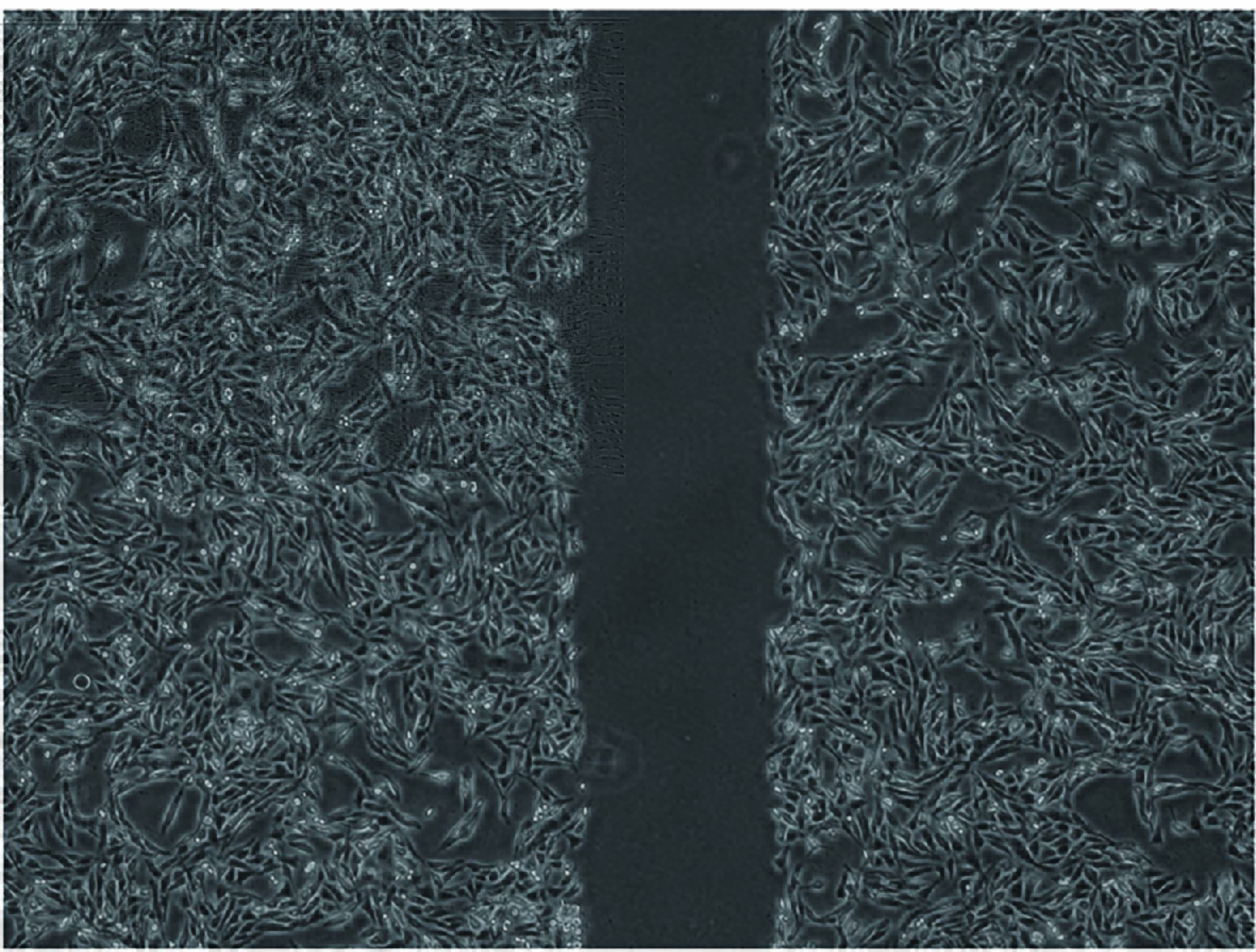

Supplement: Supplementary file 1 — Additional file 1: Supplementary Fig. 1. a In breast cancer cell line and non-tumor cell line MCF-10A, the expression of BC069792 was the highest in non-tumor cell line MCF-10A, while in breast cancer cell line, the expression of BC069792 in MDA-MB-231 and MDA-MB-468 cell lines was the lowest. b CCK-8 experiments showed that si-BC069792 can promote the proliferation of MDA-MB-231 cells (*P=0.43) and MDA-MB-468 (*P=0.026) cells. c The results of EdU experiments showed that si-BC069792 promoted the proliferation ability of breast MDA-MB-231 (*P=0.042) and MDA-MB-468 (*P=0.050) cancer cells. d Compared with the control group, the si-BC069792 knockdown group can effectively promote the migration (P=0.044) and invasion ability (P=0.002) of MDA-MB-231 cells, while the si-BC069792 knockdown group can effectively promote the migration (P=0.002) and invasion (**P=0.005) of MDA-MB-468 cells, and the number of cells passing through the underfloor membrane of the chamber is significantly increased. *P< 0.05, **P< 0.01, ***P< 0.001. Supplementary Fig. 2. Wound healing experiment confirmed that BC069792 can effectively inhibit the migration ability of breast cancer cells. Supplementary Fig. 3. Gene differential expression results after breast cancer cells overexpressed BC069792 a The results of principal component analysis showed that the consistency within the two sample groups was good and had difference. b The results of gene difference analysis showed that the BC069792 overexpression group could cause differential expression of 1209 downstream genes. c The differential expression pathway shown in the figure related to the transduction function of synaptic transmission signal. Supplementary Fig. 4. The exprssion of KCNQ4 protein in the knockdown BC069792 group was significantly reduced (*p=0.014). [file 12943_2023_1747_MOESM1_ESM.zip › Supplementary Materials/Supplementary Figure 2/2 MDA-MB-231-BC069792-0h.jpg]

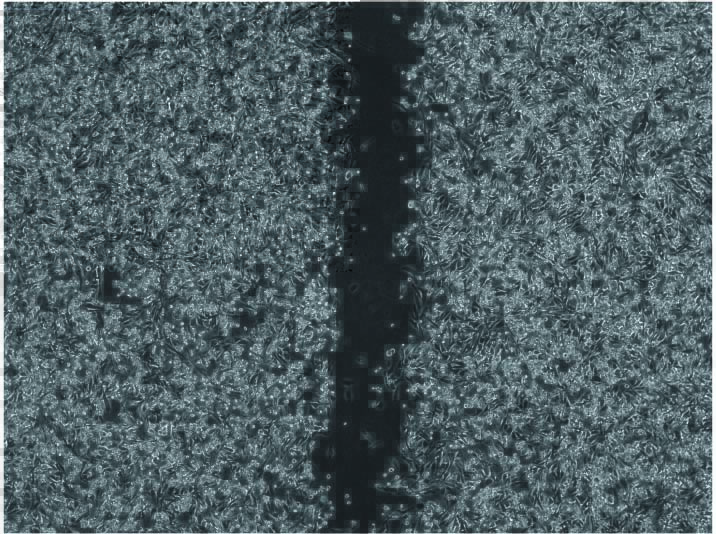

Supplement: Supplementary file 1 — Additional file 1: Supplementary Fig. 1. a In breast cancer cell line and non-tumor cell line MCF-10A, the expression of BC069792 was the highest in non-tumor cell line MCF-10A, while in breast cancer cell line, the expression of BC069792 in MDA-MB-231 and MDA-MB-468 cell lines was the lowest. b CCK-8 experiments showed that si-BC069792 can promote the proliferation of MDA-MB-231 cells (*P=0.43) and MDA-MB-468 (*P=0.026) cells. c The results of EdU experiments showed that si-BC069792 promoted the proliferation ability of breast MDA-MB-231 (*P=0.042) and MDA-MB-468 (*P=0.050) cancer cells. d Compared with the control group, the si-BC069792 knockdown group can effectively promote the migration (P=0.044) and invasion ability (P=0.002) of MDA-MB-231 cells, while the si-BC069792 knockdown group can effectively promote the migration (P=0.002) and invasion (**P=0.005) of MDA-MB-468 cells, and the number of cells passing through the underfloor membrane of the chamber is significantly increased. *P< 0.05, **P< 0.01, ***P< 0.001. Supplementary Fig. 2. Wound healing experiment confirmed that BC069792 can effectively inhibit the migration ability of breast cancer cells. Supplementary Fig. 3. Gene differential expression results after breast cancer cells overexpressed BC069792 a The results of principal component analysis showed that the consistency within the two sample groups was good and had difference. b The results of gene difference analysis showed that the BC069792 overexpression group could cause differential expression of 1209 downstream genes. c The differential expression pathway shown in the figure related to the transduction function of synaptic transmission signal. Supplementary Fig. 4. The exprssion of KCNQ4 protein in the knockdown BC069792 group was significantly reduced (*p=0.014). [file 12943_2023_1747_MOESM1_ESM.zip › Supplementary Materials/Supplementary Figure 2/2 MDA-MB-231-BC069792-48h.jpg]

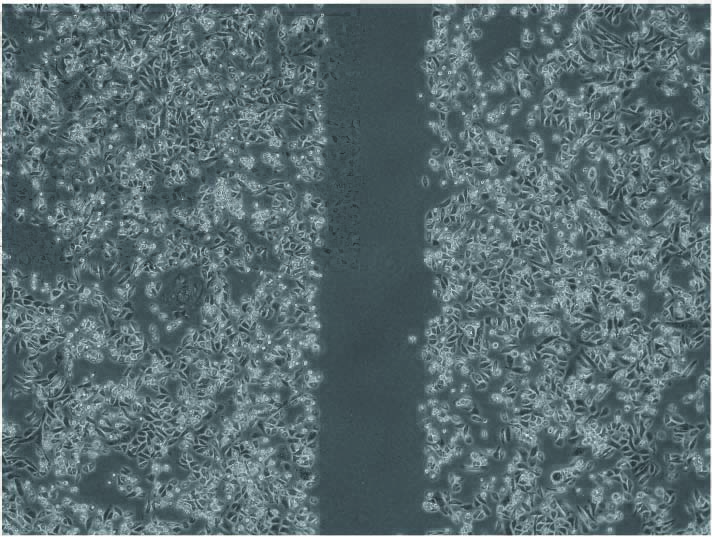

Supplement: Supplementary file 1 — Additional file 1: Supplementary Fig. 1. a In breast cancer cell line and non-tumor cell line MCF-10A, the expression of BC069792 was the highest in non-tumor cell line MCF-10A, while in breast cancer cell line, the expression of BC069792 in MDA-MB-231 and MDA-MB-468 cell lines was the lowest. b CCK-8 experiments showed that si-BC069792 can promote the proliferation of MDA-MB-231 cells (*P=0.43) and MDA-MB-468 (*P=0.026) cells. c The results of EdU experiments showed that si-BC069792 promoted the proliferation ability of breast MDA-MB-231 (*P=0.042) and MDA-MB-468 (*P=0.050) cancer cells. d Compared with the control group, the si-BC069792 knockdown group can effectively promote the migration (P=0.044) and invasion ability (P=0.002) of MDA-MB-231 cells, while the si-BC069792 knockdown group can effectively promote the migration (P=0.002) and invasion (**P=0.005) of MDA-MB-468 cells, and the number of cells passing through the underfloor membrane of the chamber is significantly increased. *P< 0.05, **P< 0.01, ***P< 0.001. Supplementary Fig. 2. Wound healing experiment confirmed that BC069792 can effectively inhibit the migration ability of breast cancer cells. Supplementary Fig. 3. Gene differential expression results after breast cancer cells overexpressed BC069792 a The results of principal component analysis showed that the consistency within the two sample groups was good and had difference. b The results of gene difference analysis showed that the BC069792 overexpression group could cause differential expression of 1209 downstream genes. c The differential expression pathway shown in the figure related to the transduction function of synaptic transmission signal. Supplementary Fig. 4. The exprssion of KCNQ4 protein in the knockdown BC069792 group was significantly reduced (*p=0.014). [file 12943_2023_1747_MOESM1_ESM.zip › Supplementary Materials/Supplementary Figure 2/2 MDA-MB-231-pc-DNA3.1-0h.jpg]

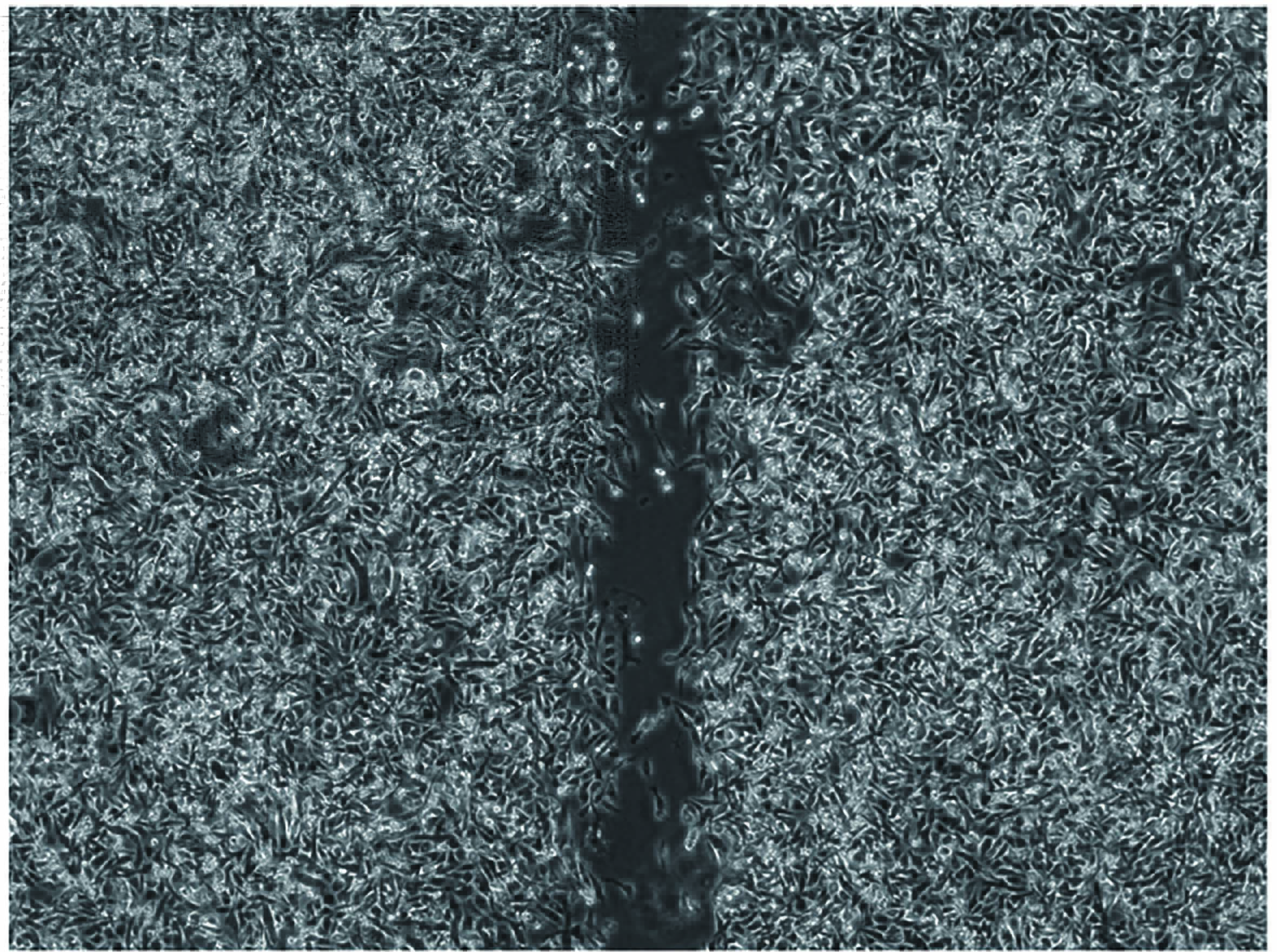

Supplement: Supplementary file 1 — Additional file 1: Supplementary Fig. 1. a In breast cancer cell line and non-tumor cell line MCF-10A, the expression of BC069792 was the highest in non-tumor cell line MCF-10A, while in breast cancer cell line, the expression of BC069792 in MDA-MB-231 and MDA-MB-468 cell lines was the lowest. b CCK-8 experiments showed that si-BC069792 can promote the proliferation of MDA-MB-231 cells (*P=0.43) and MDA-MB-468 (*P=0.026) cells. c The results of EdU experiments showed that si-BC069792 promoted the proliferation ability of breast MDA-MB-231 (*P=0.042) and MDA-MB-468 (*P=0.050) cancer cells. d Compared with the control group, the si-BC069792 knockdown group can effectively promote the migration (P=0.044) and invasion ability (P=0.002) of MDA-MB-231 cells, while the si-BC069792 knockdown group can effectively promote the migration (P=0.002) and invasion (**P=0.005) of MDA-MB-468 cells, and the number of cells passing through the underfloor membrane of the chamber is significantly increased. *P< 0.05, **P< 0.01, ***P< 0.001. Supplementary Fig. 2. Wound healing experiment confirmed that BC069792 can effectively inhibit the migration ability of breast cancer cells. Supplementary Fig. 3. Gene differential expression results after breast cancer cells overexpressed BC069792 a The results of principal component analysis showed that the consistency within the two sample groups was good and had difference. b The results of gene difference analysis showed that the BC069792 overexpression group could cause differential expression of 1209 downstream genes. c The differential expression pathway shown in the figure related to the transduction function of synaptic transmission signal. Supplementary Fig. 4. The exprssion of KCNQ4 protein in the knockdown BC069792 group was significantly reduced (*p=0.014). [file 12943_2023_1747_MOESM1_ESM.zip › Supplementary Materials/Supplementary Figure 2/2 MDA-MB-231-pcDNA3.1-48h.jpg]

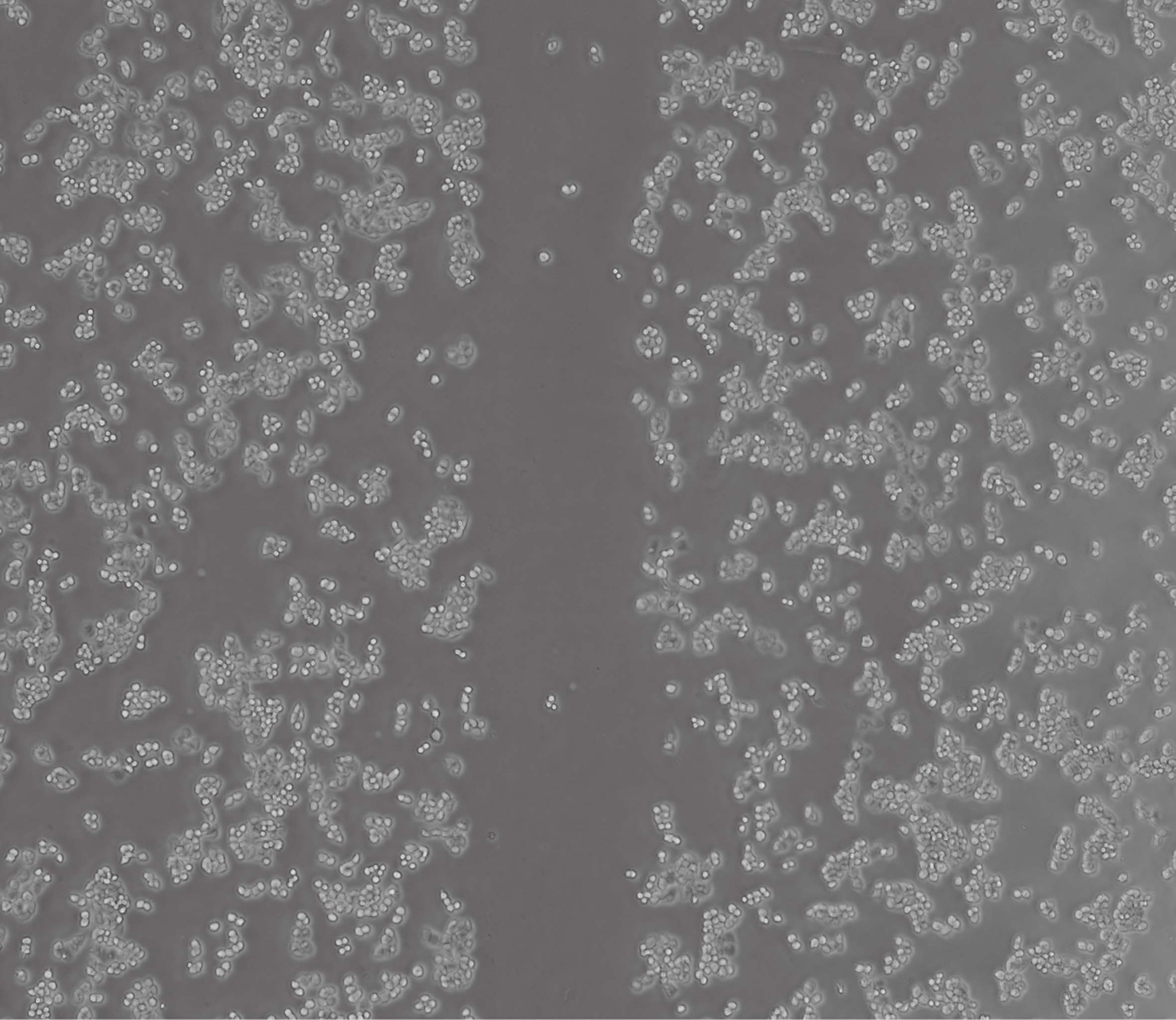

Supplement: Supplementary file 1 — Additional file 1: Supplementary Fig. 1. a In breast cancer cell line and non-tumor cell line MCF-10A, the expression of BC069792 was the highest in non-tumor cell line MCF-10A, while in breast cancer cell line, the expression of BC069792 in MDA-MB-231 and MDA-MB-468 cell lines was the lowest. b CCK-8 experiments showed that si-BC069792 can promote the proliferation of MDA-MB-231 cells (*P=0.43) and MDA-MB-468 (*P=0.026) cells. c The results of EdU experiments showed that si-BC069792 promoted the proliferation ability of breast MDA-MB-231 (*P=0.042) and MDA-MB-468 (*P=0.050) cancer cells. d Compared with the control group, the si-BC069792 knockdown group can effectively promote the migration (P=0.044) and invasion ability (P=0.002) of MDA-MB-231 cells, while the si-BC069792 knockdown group can effectively promote the migration (P=0.002) and invasion (**P=0.005) of MDA-MB-468 cells, and the number of cells passing through the underfloor membrane of the chamber is significantly increased. *P< 0.05, **P< 0.01, ***P< 0.001. Supplementary Fig. 2. Wound healing experiment confirmed that BC069792 can effectively inhibit the migration ability of breast cancer cells. Supplementary Fig. 3. Gene differential expression results after breast cancer cells overexpressed BC069792 a The results of principal component analysis showed that the consistency within the two sample groups was good and had difference. b The results of gene difference analysis showed that the BC069792 overexpression group could cause differential expression of 1209 downstream genes. c The differential expression pathway shown in the figure related to the transduction function of synaptic transmission signal. Supplementary Fig. 4. The exprssion of KCNQ4 protein in the knockdown BC069792 group was significantly reduced (*p=0.014). [file 12943_2023_1747_MOESM1_ESM.zip › Supplementary Materials/Supplementary Figure 2/2 MDA-MB-468-BC069792-0h.jpg]

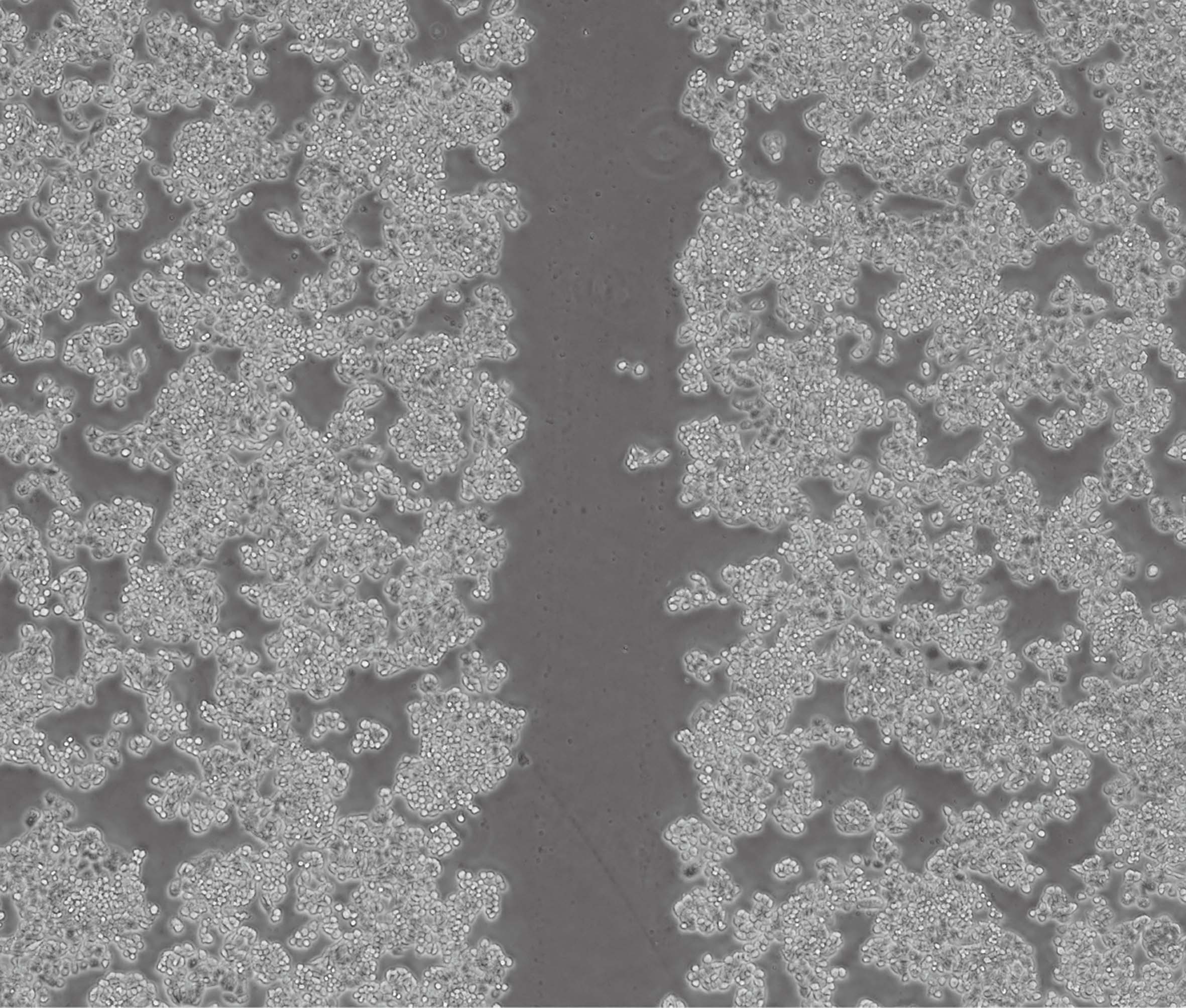

Supplement: Supplementary file 1 — Additional file 1: Supplementary Fig. 1. a In breast cancer cell line and non-tumor cell line MCF-10A, the expression of BC069792 was the highest in non-tumor cell line MCF-10A, while in breast cancer cell line, the expression of BC069792 in MDA-MB-231 and MDA-MB-468 cell lines was the lowest. b CCK-8 experiments showed that si-BC069792 can promote the proliferation of MDA-MB-231 cells (*P=0.43) and MDA-MB-468 (*P=0.026) cells. c The results of EdU experiments showed that si-BC069792 promoted the proliferation ability of breast MDA-MB-231 (*P=0.042) and MDA-MB-468 (*P=0.050) cancer cells. d Compared with the control group, the si-BC069792 knockdown group can effectively promote the migration (P=0.044) and invasion ability (P=0.002) of MDA-MB-231 cells, while the si-BC069792 knockdown group can effectively promote the migration (P=0.002) and invasion (**P=0.005) of MDA-MB-468 cells, and the number of cells passing through the underfloor membrane of the chamber is significantly increased. *P< 0.05, **P< 0.01, ***P< 0.001. Supplementary Fig. 2. Wound healing experiment confirmed that BC069792 can effectively inhibit the migration ability of breast cancer cells. Supplementary Fig. 3. Gene differential expression results after breast cancer cells overexpressed BC069792 a The results of principal component analysis showed that the consistency within the two sample groups was good and had difference. b The results of gene difference analysis showed that the BC069792 overexpression group could cause differential expression of 1209 downstream genes. c The differential expression pathway shown in the figure related to the transduction function of synaptic transmission signal. Supplementary Fig. 4. The exprssion of KCNQ4 protein in the knockdown BC069792 group was significantly reduced (*p=0.014). [file 12943_2023_1747_MOESM1_ESM.zip › Supplementary Materials/Supplementary Figure 2/2 MDA-MB-468-BC069792-72h.jpg]

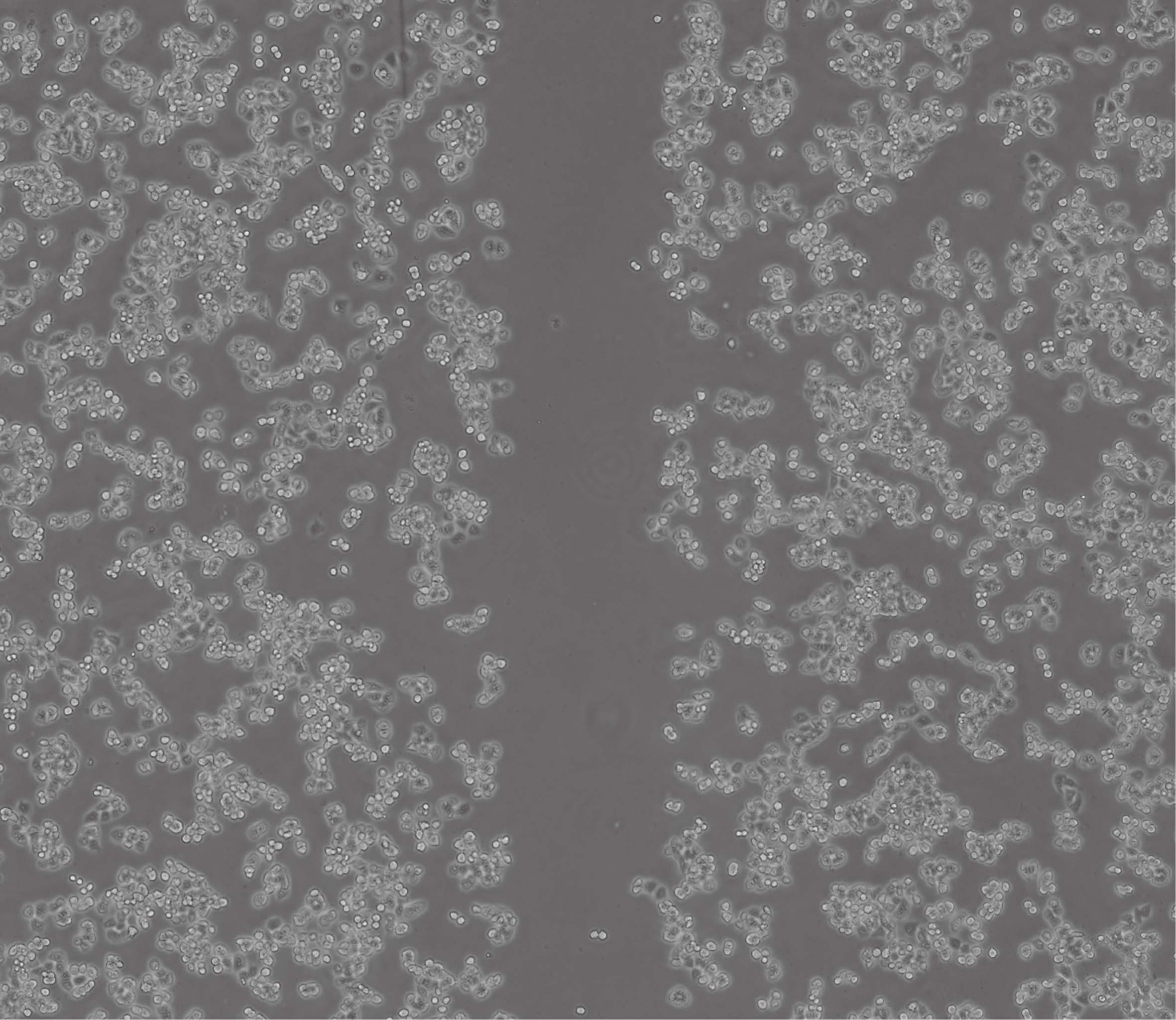

Supplement: Supplementary file 1 — Additional file 1: Supplementary Fig. 1. a In breast cancer cell line and non-tumor cell line MCF-10A, the expression of BC069792 was the highest in non-tumor cell line MCF-10A, while in breast cancer cell line, the expression of BC069792 in MDA-MB-231 and MDA-MB-468 cell lines was the lowest. b CCK-8 experiments showed that si-BC069792 can promote the proliferation of MDA-MB-231 cells (*P=0.43) and MDA-MB-468 (*P=0.026) cells. c The results of EdU experiments showed that si-BC069792 promoted the proliferation ability of breast MDA-MB-231 (*P=0.042) and MDA-MB-468 (*P=0.050) cancer cells. d Compared with the control group, the si-BC069792 knockdown group can effectively promote the migration (P=0.044) and invasion ability (P=0.002) of MDA-MB-231 cells, while the si-BC069792 knockdown group can effectively promote the migration (P=0.002) and invasion (**P=0.005) of MDA-MB-468 cells, and the number of cells passing through the underfloor membrane of the chamber is significantly increased. *P< 0.05, **P< 0.01, ***P< 0.001. Supplementary Fig. 2. Wound healing experiment confirmed that BC069792 can effectively inhibit the migration ability of breast cancer cells. Supplementary Fig. 3. Gene differential expression results after breast cancer cells overexpressed BC069792 a The results of principal component analysis showed that the consistency within the two sample groups was good and had difference. b The results of gene difference analysis showed that the BC069792 overexpression group could cause differential expression of 1209 downstream genes. c The differential expression pathway shown in the figure related to the transduction function of synaptic transmission signal. Supplementary Fig. 4. The exprssion of KCNQ4 protein in the knockdown BC069792 group was significantly reduced (*p=0.014). [file 12943_2023_1747_MOESM1_ESM.zip › Supplementary Materials/Supplementary Figure 2/2 MDA-MB-468-pc-DNA3.1-0h.jpg]

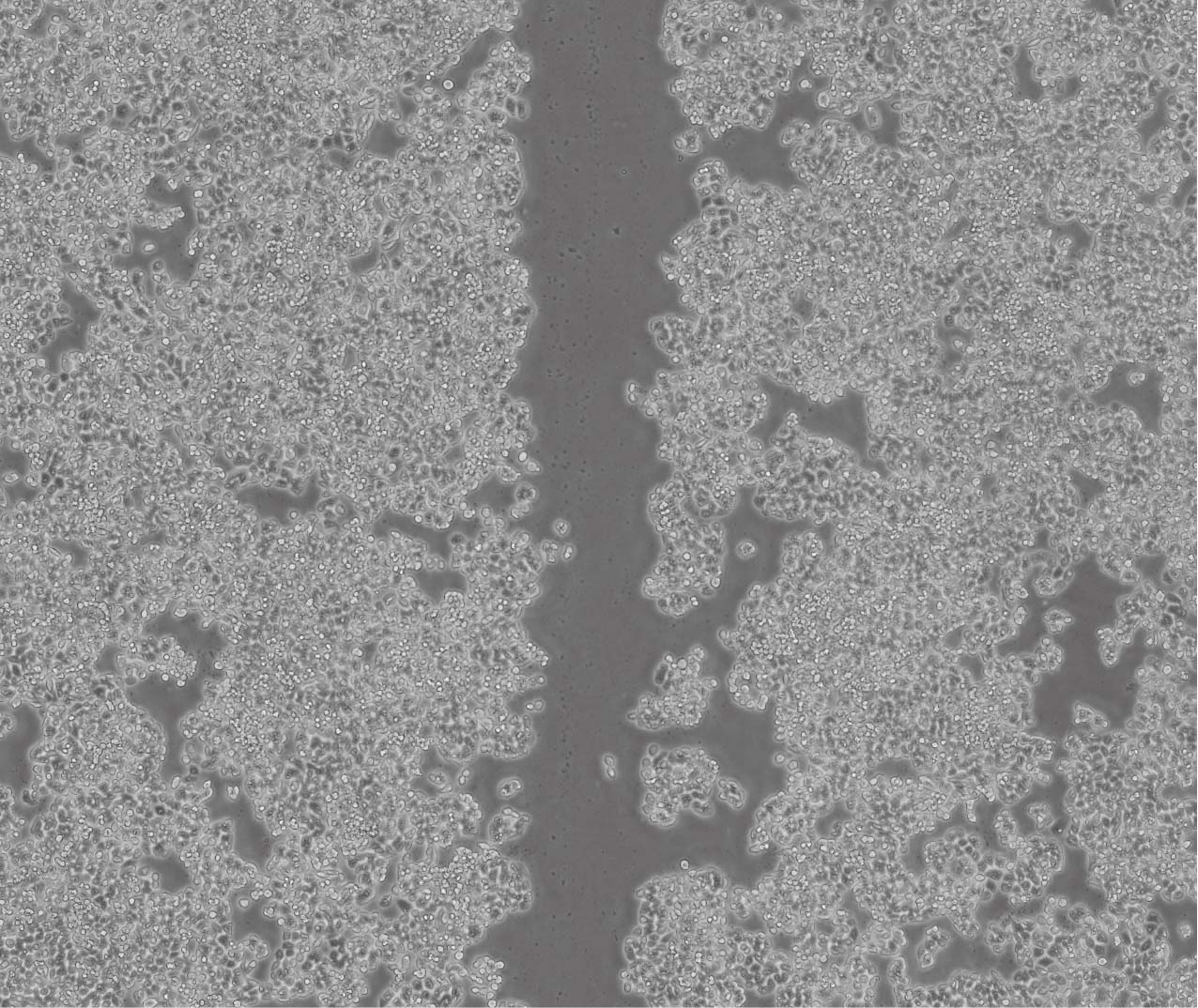

Supplement: Supplementary file 1 — Additional file 1: Supplementary Fig. 1. a In breast cancer cell line and non-tumor cell line MCF-10A, the expression of BC069792 was the highest in non-tumor cell line MCF-10A, while in breast cancer cell line, the expression of BC069792 in MDA-MB-231 and MDA-MB-468 cell lines was the lowest. b CCK-8 experiments showed that si-BC069792 can promote the proliferation of MDA-MB-231 cells (*P=0.43) and MDA-MB-468 (*P=0.026) cells. c The results of EdU experiments showed that si-BC069792 promoted the proliferation ability of breast MDA-MB-231 (*P=0.042) and MDA-MB-468 (*P=0.050) cancer cells. d Compared with the control group, the si-BC069792 knockdown group can effectively promote the migration (P=0.044) and invasion ability (P=0.002) of MDA-MB-231 cells, while the si-BC069792 knockdown group can effectively promote the migration (P=0.002) and invasion (**P=0.005) of MDA-MB-468 cells, and the number of cells passing through the underfloor membrane of the chamber is significantly increased. *P< 0.05, **P< 0.01, ***P< 0.001. Supplementary Fig. 2. Wound healing experiment confirmed that BC069792 can effectively inhibit the migration ability of breast cancer cells. Supplementary Fig. 3. Gene differential expression results after breast cancer cells overexpressed BC069792 a The results of principal component analysis showed that the consistency within the two sample groups was good and had difference. b The results of gene difference analysis showed that the BC069792 overexpression group could cause differential expression of 1209 downstream genes. c The differential expression pathway shown in the figure related to the transduction function of synaptic transmission signal. Supplementary Fig. 4. The exprssion of KCNQ4 protein in the knockdown BC069792 group was significantly reduced (*p=0.014). [file 12943_2023_1747_MOESM1_ESM.zip › Supplementary Materials/Supplementary Figure 2/2 MDA-MB-468-pc-DNA3.1-72h.jpg]

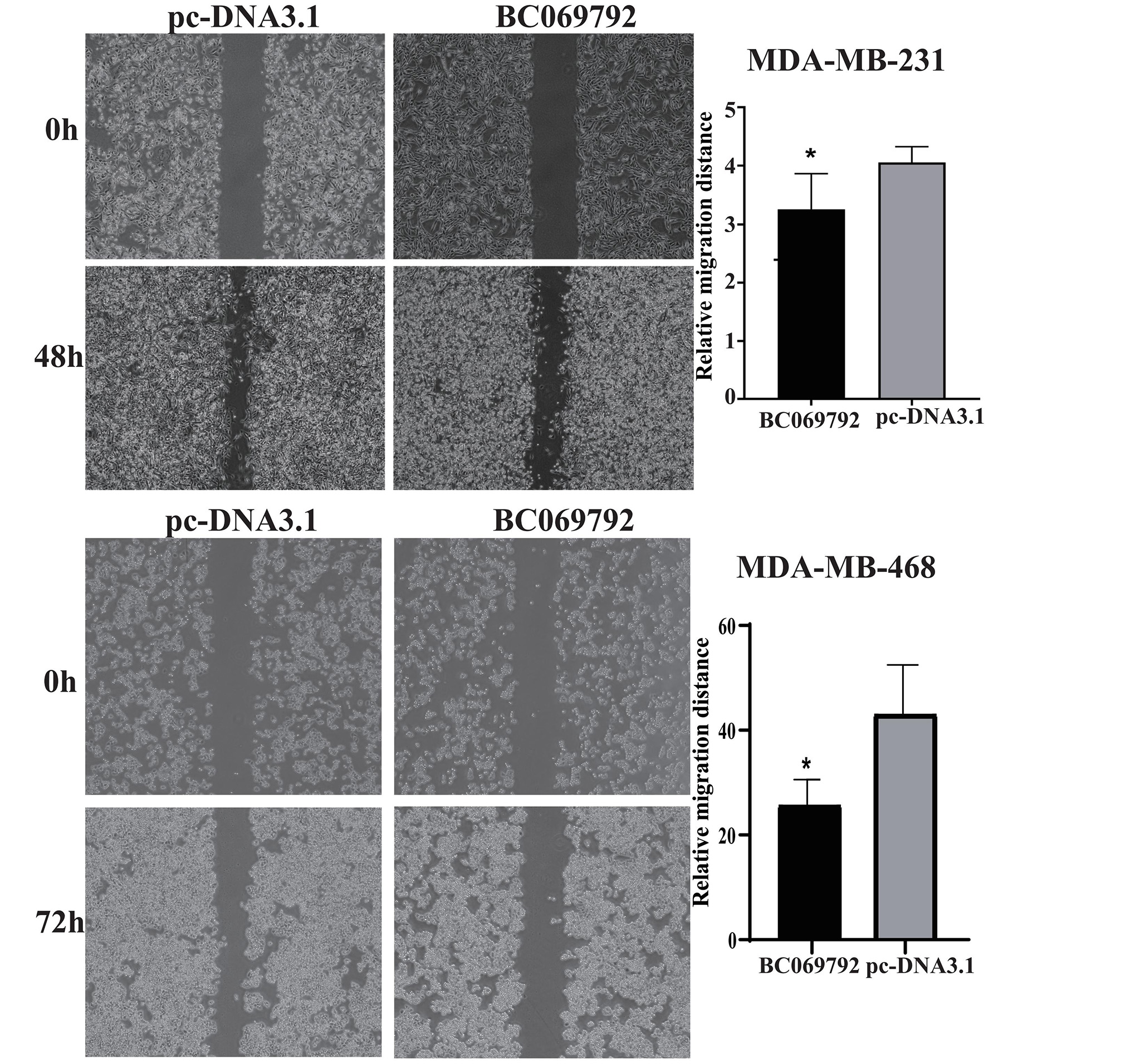

Supplement: Supplementary file 1 — Additional file 1: Supplementary Fig. 1. a In breast cancer cell line and non-tumor cell line MCF-10A, the expression of BC069792 was the highest in non-tumor cell line MCF-10A, while in breast cancer cell line, the expression of BC069792 in MDA-MB-231 and MDA-MB-468 cell lines was the lowest. b CCK-8 experiments showed that si-BC069792 can promote the proliferation of MDA-MB-231 cells (*P=0.43) and MDA-MB-468 (*P=0.026) cells. c The results of EdU experiments showed that si-BC069792 promoted the proliferation ability of breast MDA-MB-231 (*P=0.042) and MDA-MB-468 (*P=0.050) cancer cells. d Compared with the control group, the si-BC069792 knockdown group can effectively promote the migration (P=0.044) and invasion ability (P=0.002) of MDA-MB-231 cells, while the si-BC069792 knockdown group can effectively promote the migration (P=0.002) and invasion (**P=0.005) of MDA-MB-468 cells, and the number of cells passing through the underfloor membrane of the chamber is significantly increased. *P< 0.05, **P< 0.01, ***P< 0.001. Supplementary Fig. 2. Wound healing experiment confirmed that BC069792 can effectively inhibit the migration ability of breast cancer cells. Supplementary Fig. 3. Gene differential expression results after breast cancer cells overexpressed BC069792 a The results of principal component analysis showed that the consistency within the two sample groups was good and had difference. b The results of gene difference analysis showed that the BC069792 overexpression group could cause differential expression of 1209 downstream genes. c The differential expression pathway shown in the figure related to the transduction function of synaptic transmission signal. Supplementary Fig. 4. The exprssion of KCNQ4 protein in the knockdown BC069792 group was significantly reduced (*p=0.014). [file 12943_2023_1747_MOESM1_ESM.zip › Supplementary Materials/Supplementary Figure 2/sup. 2.jpg]

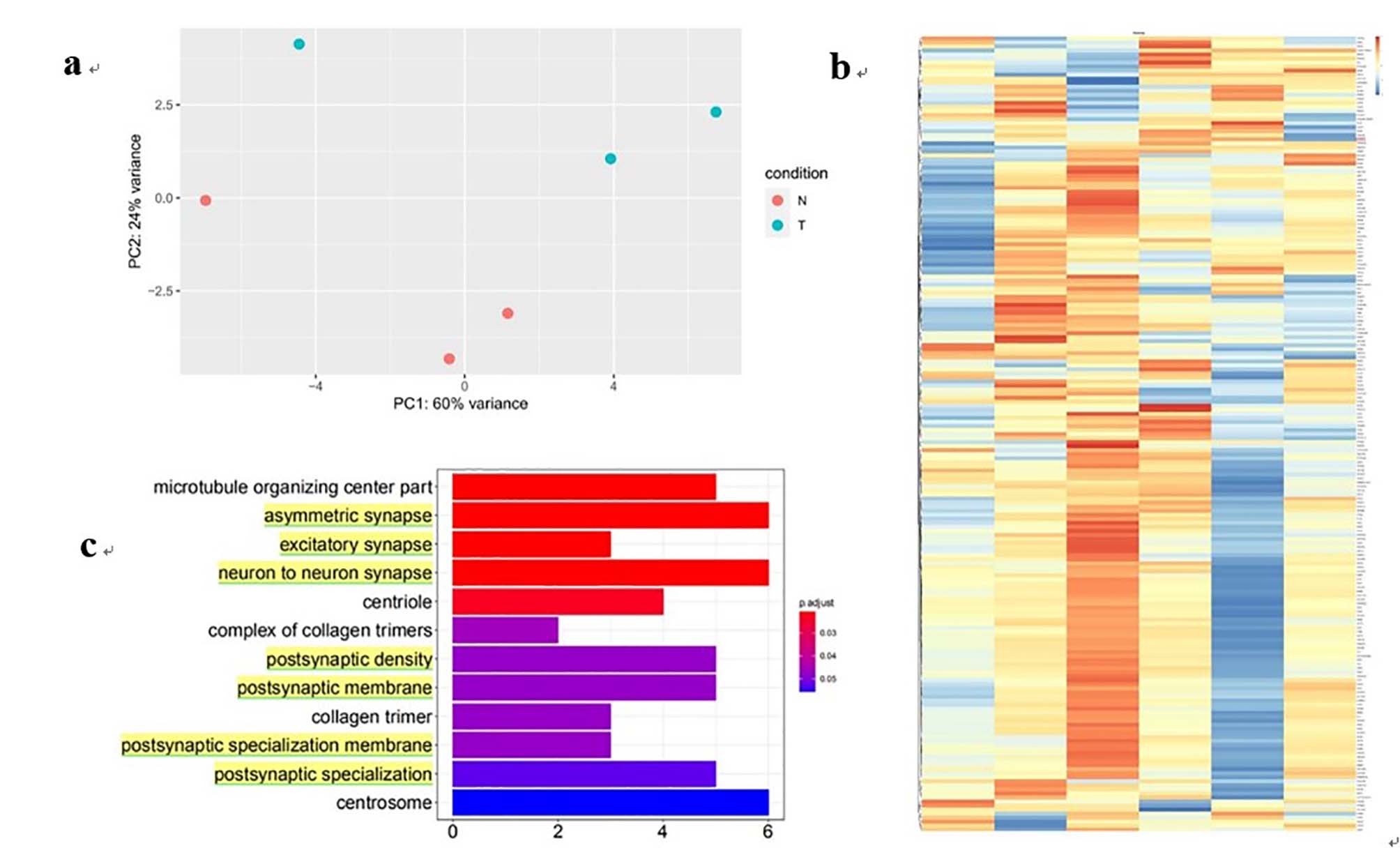

Supplement: Supplementary file 1 — Additional file 1: Supplementary Fig. 1. a In breast cancer cell line and non-tumor cell line MCF-10A, the expression of BC069792 was the highest in non-tumor cell line MCF-10A, while in breast cancer cell line, the expression of BC069792 in MDA-MB-231 and MDA-MB-468 cell lines was the lowest. b CCK-8 experiments showed that si-BC069792 can promote the proliferation of MDA-MB-231 cells (*P=0.43) and MDA-MB-468 (*P=0.026) cells. c The results of EdU experiments showed that si-BC069792 promoted the proliferation ability of breast MDA-MB-231 (*P=0.042) and MDA-MB-468 (*P=0.050) cancer cells. d Compared with the control group, the si-BC069792 knockdown group can effectively promote the migration (P=0.044) and invasion ability (P=0.002) of MDA-MB-231 cells, while the si-BC069792 knockdown group can effectively promote the migration (P=0.002) and invasion (**P=0.005) of MDA-MB-468 cells, and the number of cells passing through the underfloor membrane of the chamber is significantly increased. *P< 0.05, **P< 0.01, ***P< 0.001. Supplementary Fig. 2. Wound healing experiment confirmed that BC069792 can effectively inhibit the migration ability of breast cancer cells. Supplementary Fig. 3. Gene differential expression results after breast cancer cells overexpressed BC069792 a The results of principal component analysis showed that the consistency within the two sample groups was good and had difference. b The results of gene difference analysis showed that the BC069792 overexpression group could cause differential expression of 1209 downstream genes. c The differential expression pathway shown in the figure related to the transduction function of synaptic transmission signal. Supplementary Fig. 4. The exprssion of KCNQ4 protein in the knockdown BC069792 group was significantly reduced (*p=0.014). [file 12943_2023_1747_MOESM1_ESM.zip › Supplementary Materials/Supplementary Figure 3/sup. 3.jpg]

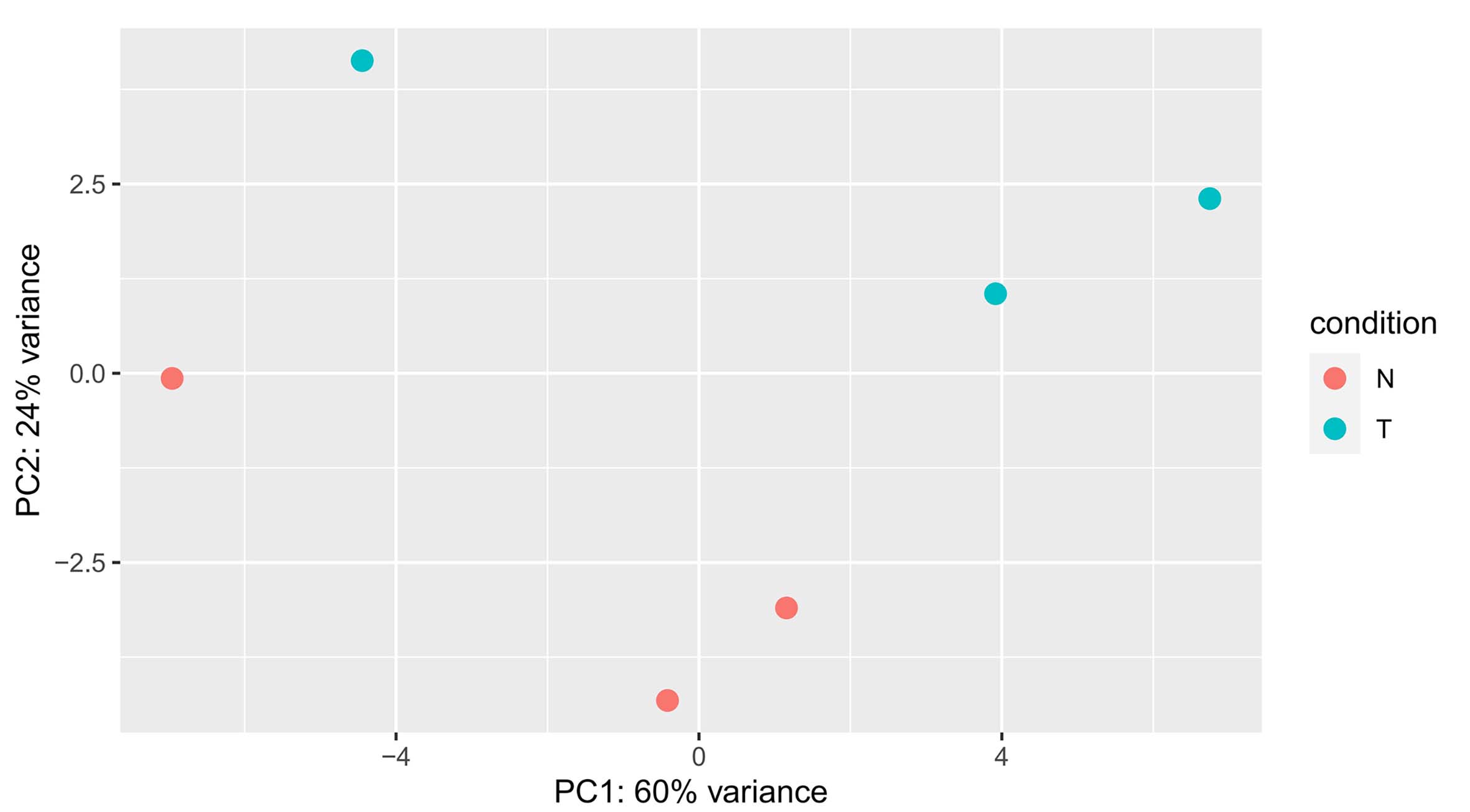

Supplement: Supplementary file 1 — Additional file 1: Supplementary Fig. 1. a In breast cancer cell line and non-tumor cell line MCF-10A, the expression of BC069792 was the highest in non-tumor cell line MCF-10A, while in breast cancer cell line, the expression of BC069792 in MDA-MB-231 and MDA-MB-468 cell lines was the lowest. b CCK-8 experiments showed that si-BC069792 can promote the proliferation of MDA-MB-231 cells (*P=0.43) and MDA-MB-468 (*P=0.026) cells. c The results of EdU experiments showed that si-BC069792 promoted the proliferation ability of breast MDA-MB-231 (*P=0.042) and MDA-MB-468 (*P=0.050) cancer cells. d Compared with the control group, the si-BC069792 knockdown group can effectively promote the migration (P=0.044) and invasion ability (P=0.002) of MDA-MB-231 cells, while the si-BC069792 knockdown group can effectively promote the migration (P=0.002) and invasion (**P=0.005) of MDA-MB-468 cells, and the number of cells passing through the underfloor membrane of the chamber is significantly increased. *P< 0.05, **P< 0.01, ***P< 0.001. Supplementary Fig. 2. Wound healing experiment confirmed that BC069792 can effectively inhibit the migration ability of breast cancer cells. Supplementary Fig. 3. Gene differential expression results after breast cancer cells overexpressed BC069792 a The results of principal component analysis showed that the consistency within the two sample groups was good and had difference. b The results of gene difference analysis showed that the BC069792 overexpression group could cause differential expression of 1209 downstream genes. c The differential expression pathway shown in the figure related to the transduction function of synaptic transmission signal. Supplementary Fig. 4. The exprssion of KCNQ4 protein in the knockdown BC069792 group was significantly reduced (*p=0.014). [file 12943_2023_1747_MOESM1_ESM.zip › Supplementary Materials/Supplementary Figure 3/sup. 3a.jpg]

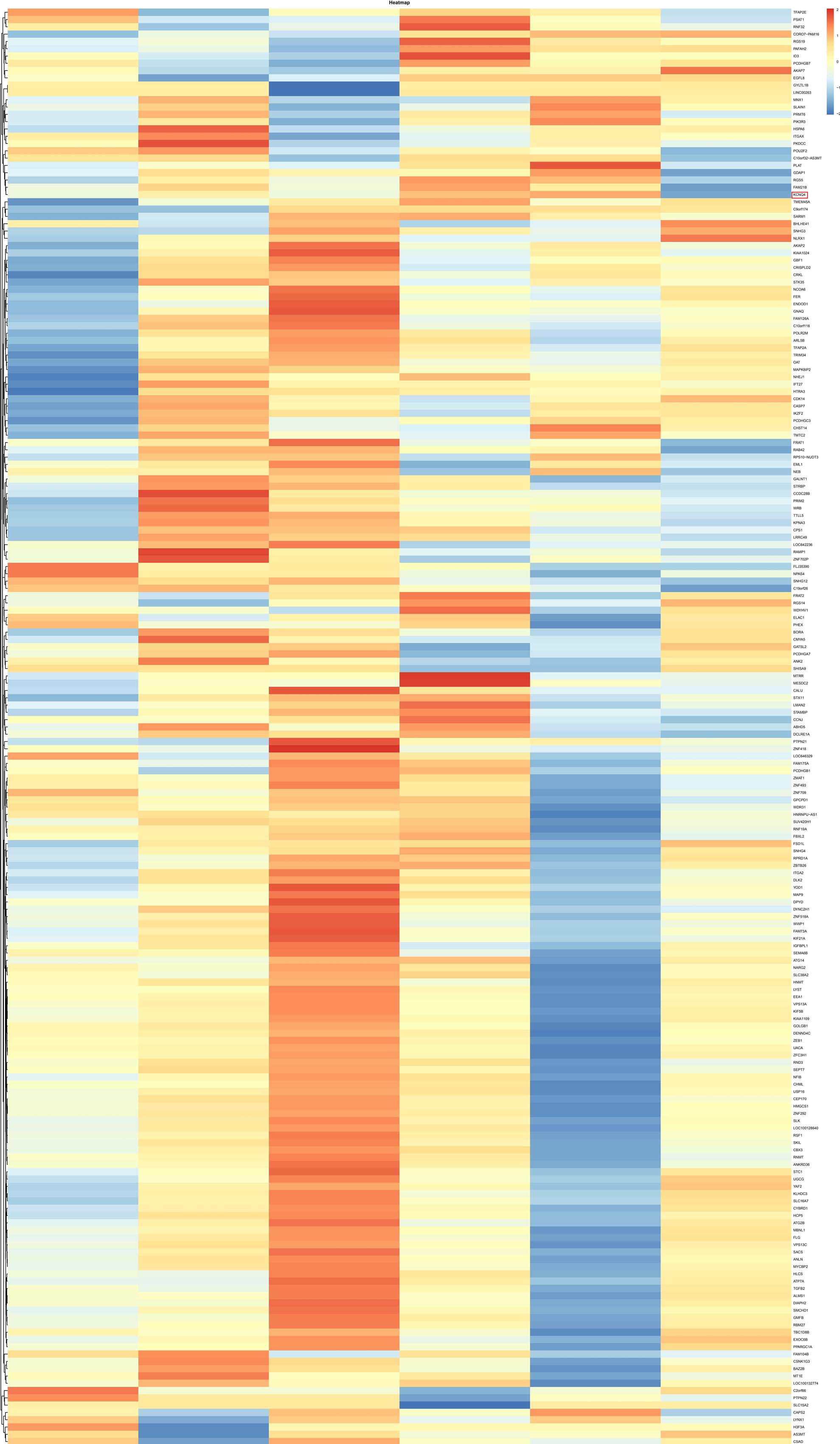

Supplement: Supplementary file 1 — Additional file 1: Supplementary Fig. 1. a In breast cancer cell line and non-tumor cell line MCF-10A, the expression of BC069792 was the highest in non-tumor cell line MCF-10A, while in breast cancer cell line, the expression of BC069792 in MDA-MB-231 and MDA-MB-468 cell lines was the lowest. b CCK-8 experiments showed that si-BC069792 can promote the proliferation of MDA-MB-231 cells (*P=0.43) and MDA-MB-468 (*P=0.026) cells. c The results of EdU experiments showed that si-BC069792 promoted the proliferation ability of breast MDA-MB-231 (*P=0.042) and MDA-MB-468 (*P=0.050) cancer cells. d Compared with the control group, the si-BC069792 knockdown group can effectively promote the migration (P=0.044) and invasion ability (P=0.002) of MDA-MB-231 cells, while the si-BC069792 knockdown group can effectively promote the migration (P=0.002) and invasion (**P=0.005) of MDA-MB-468 cells, and the number of cells passing through the underfloor membrane of the chamber is significantly increased. *P< 0.05, **P< 0.01, ***P< 0.001. Supplementary Fig. 2. Wound healing experiment confirmed that BC069792 can effectively inhibit the migration ability of breast cancer cells. Supplementary Fig. 3. Gene differential expression results after breast cancer cells overexpressed BC069792 a The results of principal component analysis showed that the consistency within the two sample groups was good and had difference. b The results of gene difference analysis showed that the BC069792 overexpression group could cause differential expression of 1209 downstream genes. c The differential expression pathway shown in the figure related to the transduction function of synaptic transmission signal. Supplementary Fig. 4. The exprssion of KCNQ4 protein in the knockdown BC069792 group was significantly reduced (*p=0.014). [file 12943_2023_1747_MOESM1_ESM.zip › Supplementary Materials/Supplementary Figure 3/sup. 3b.jpg]

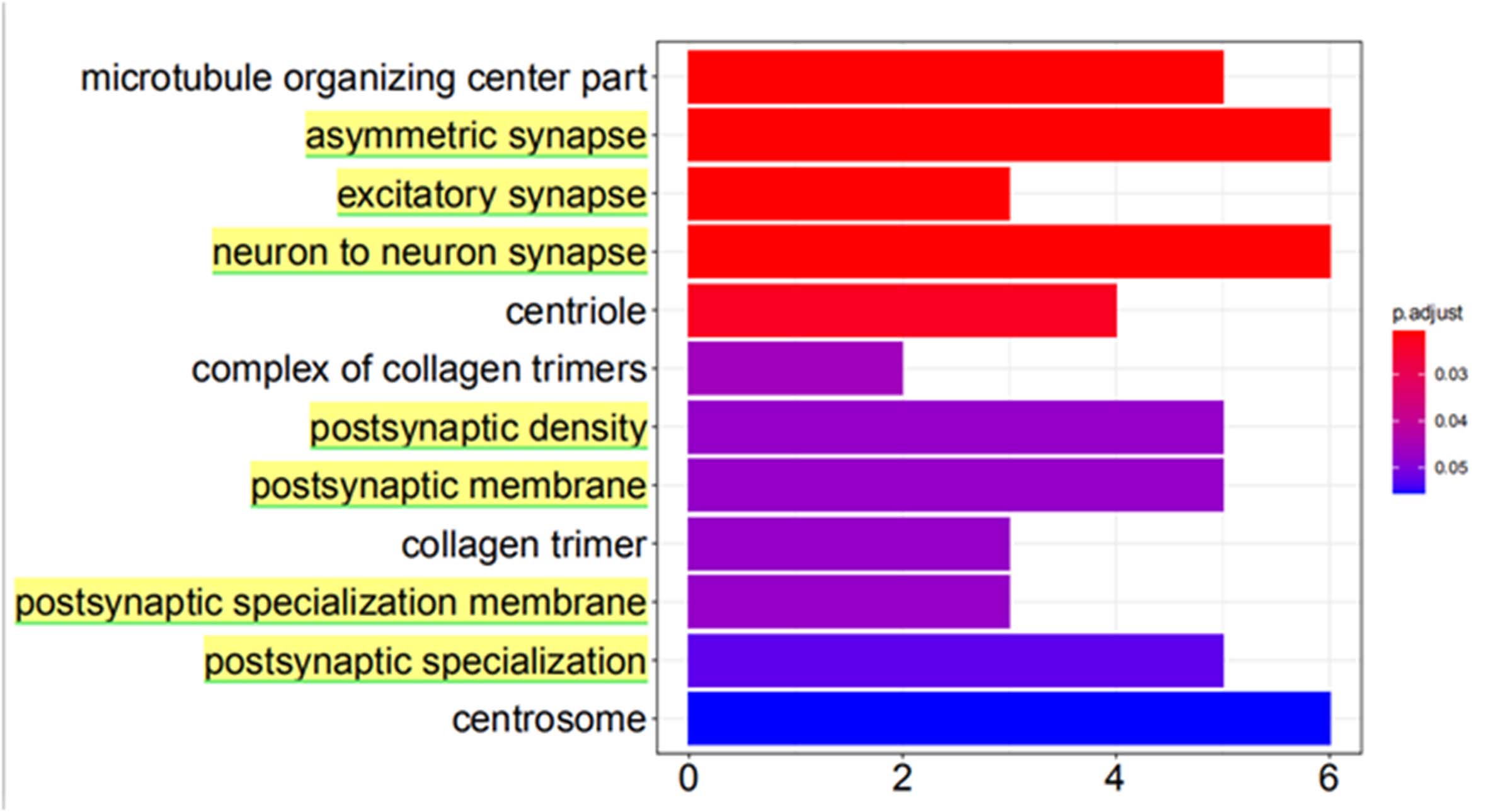

Supplement: Supplementary file 1 — Additional file 1: Supplementary Fig. 1. a In breast cancer cell line and non-tumor cell line MCF-10A, the expression of BC069792 was the highest in non-tumor cell line MCF-10A, while in breast cancer cell line, the expression of BC069792 in MDA-MB-231 and MDA-MB-468 cell lines was the lowest. b CCK-8 experiments showed that si-BC069792 can promote the proliferation of MDA-MB-231 cells (*P=0.43) and MDA-MB-468 (*P=0.026) cells. c The results of EdU experiments showed that si-BC069792 promoted the proliferation ability of breast MDA-MB-231 (*P=0.042) and MDA-MB-468 (*P=0.050) cancer cells. d Compared with the control group, the si-BC069792 knockdown group can effectively promote the migration (P=0.044) and invasion ability (P=0.002) of MDA-MB-231 cells, while the si-BC069792 knockdown group can effectively promote the migration (P=0.002) and invasion (**P=0.005) of MDA-MB-468 cells, and the number of cells passing through the underfloor membrane of the chamber is significantly increased. *P< 0.05, **P< 0.01, ***P< 0.001. Supplementary Fig. 2. Wound healing experiment confirmed that BC069792 can effectively inhibit the migration ability of breast cancer cells. Supplementary Fig. 3. Gene differential expression results after breast cancer cells overexpressed BC069792 a The results of principal component analysis showed that the consistency within the two sample groups was good and had difference. b The results of gene difference analysis showed that the BC069792 overexpression group could cause differential expression of 1209 downstream genes. c The differential expression pathway shown in the figure related to the transduction function of synaptic transmission signal. Supplementary Fig. 4. The exprssion of KCNQ4 protein in the knockdown BC069792 group was significantly reduced (*p=0.014). [file 12943_2023_1747_MOESM1_ESM.zip › Supplementary Materials/Supplementary Figure 3/sup. 3c.jpg]

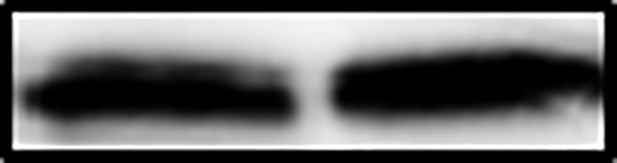

Supplement: Supplementary file 1 — Additional file 1: Supplementary Fig. 1. a In breast cancer cell line and non-tumor cell line MCF-10A, the expression of BC069792 was the highest in non-tumor cell line MCF-10A, while in breast cancer cell line, the expression of BC069792 in MDA-MB-231 and MDA-MB-468 cell lines was the lowest. b CCK-8 experiments showed that si-BC069792 can promote the proliferation of MDA-MB-231 cells (*P=0.43) and MDA-MB-468 (*P=0.026) cells. c The results of EdU experiments showed that si-BC069792 promoted the proliferation ability of breast MDA-MB-231 (*P=0.042) and MDA-MB-468 (*P=0.050) cancer cells. d Compared with the control group, the si-BC069792 knockdown group can effectively promote the migration (P=0.044) and invasion ability (P=0.002) of MDA-MB-231 cells, while the si-BC069792 knockdown group can effectively promote the migration (P=0.002) and invasion (**P=0.005) of MDA-MB-468 cells, and the number of cells passing through the underfloor membrane of the chamber is significantly increased. *P< 0.05, **P< 0.01, ***P< 0.001. Supplementary Fig. 2. Wound healing experiment confirmed that BC069792 can effectively inhibit the migration ability of breast cancer cells. Supplementary Fig. 3. Gene differential expression results after breast cancer cells overexpressed BC069792 a The results of principal component analysis showed that the consistency within the two sample groups was good and had difference. b The results of gene difference analysis showed that the BC069792 overexpression group could cause differential expression of 1209 downstream genes. c The differential expression pathway shown in the figure related to the transduction function of synaptic transmission signal. Supplementary Fig. 4. The exprssion of KCNQ4 protein in the knockdown BC069792 group was significantly reduced (*p=0.014). [file 12943_2023_1747_MOESM1_ESM.zip › Supplementary Materials/Supplementary Figure 4/GAPDH.jpg]

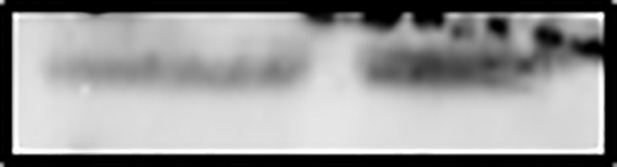

Supplement: Supplementary file 1 — Additional file 1: Supplementary Fig. 1. a In breast cancer cell line and non-tumor cell line MCF-10A, the expression of BC069792 was the highest in non-tumor cell line MCF-10A, while in breast cancer cell line, the expression of BC069792 in MDA-MB-231 and MDA-MB-468 cell lines was the lowest. b CCK-8 experiments showed that si-BC069792 can promote the proliferation of MDA-MB-231 cells (*P=0.43) and MDA-MB-468 (*P=0.026) cells. c The results of EdU experiments showed that si-BC069792 promoted the proliferation ability of breast MDA-MB-231 (*P=0.042) and MDA-MB-468 (*P=0.050) cancer cells. d Compared with the control group, the si-BC069792 knockdown group can effectively promote the migration (P=0.044) and invasion ability (P=0.002) of MDA-MB-231 cells, while the si-BC069792 knockdown group can effectively promote the migration (P=0.002) and invasion (**P=0.005) of MDA-MB-468 cells, and the number of cells passing through the underfloor membrane of the chamber is significantly increased. *P< 0.05, **P< 0.01, ***P< 0.001. Supplementary Fig. 2. Wound healing experiment confirmed that BC069792 can effectively inhibit the migration ability of breast cancer cells. Supplementary Fig. 3. Gene differential expression results after breast cancer cells overexpressed BC069792 a The results of principal component analysis showed that the consistency within the two sample groups was good and had difference. b The results of gene difference analysis showed that the BC069792 overexpression group could cause differential expression of 1209 downstream genes. c The differential expression pathway shown in the figure related to the transduction function of synaptic transmission signal. Supplementary Fig. 4. The exprssion of KCNQ4 protein in the knockdown BC069792 group was significantly reduced (*p=0.014). [file 12943_2023_1747_MOESM1_ESM.zip › Supplementary Materials/Supplementary Figure 4/KCNQ4.jpg]

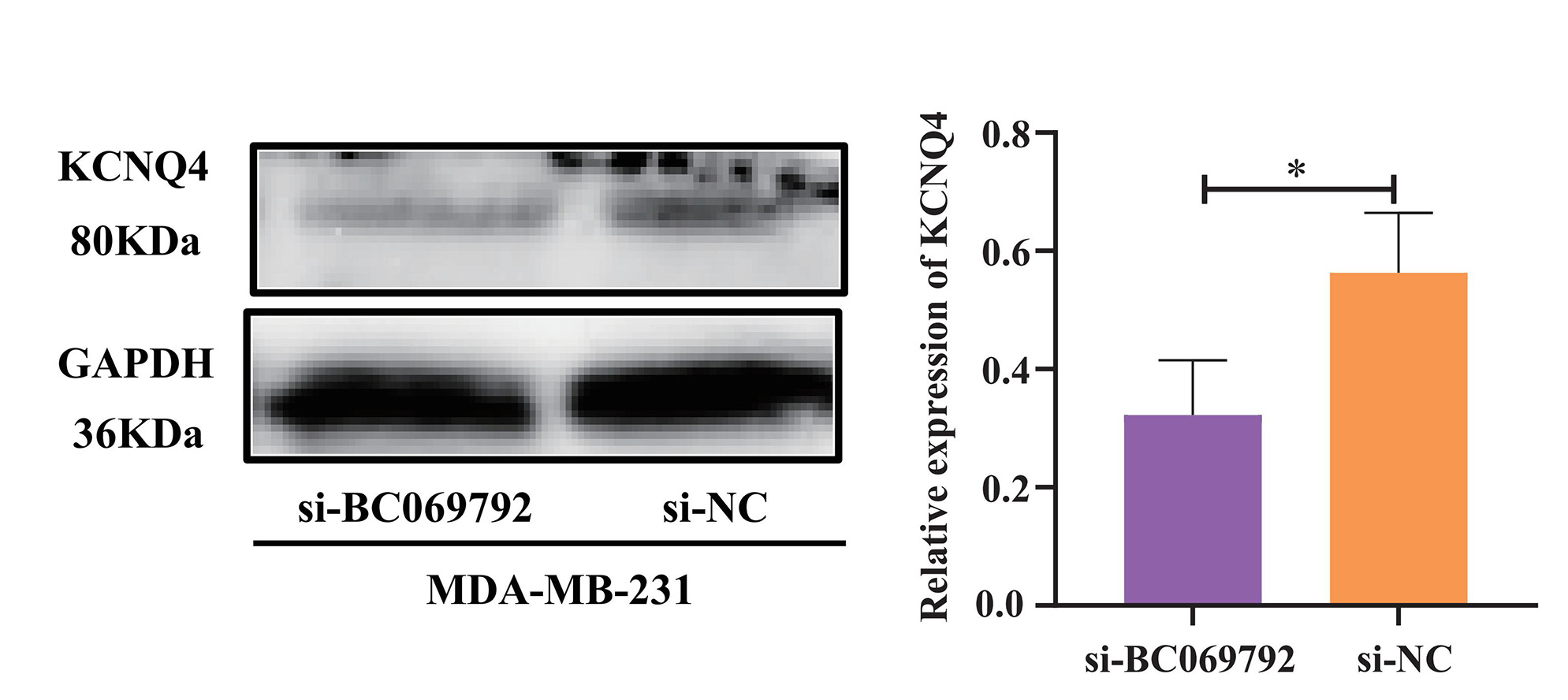

Supplement: Supplementary file 1 — Additional file 1: Supplementary Fig. 1. a In breast cancer cell line and non-tumor cell line MCF-10A, the expression of BC069792 was the highest in non-tumor cell line MCF-10A, while in breast cancer cell line, the expression of BC069792 in MDA-MB-231 and MDA-MB-468 cell lines was the lowest. b CCK-8 experiments showed that si-BC069792 can promote the proliferation of MDA-MB-231 cells (*P=0.43) and MDA-MB-468 (*P=0.026) cells. c The results of EdU experiments showed that si-BC069792 promoted the proliferation ability of breast MDA-MB-231 (*P=0.042) and MDA-MB-468 (*P=0.050) cancer cells. d Compared with the control group, the si-BC069792 knockdown group can effectively promote the migration (P=0.044) and invasion ability (P=0.002) of MDA-MB-231 cells, while the si-BC069792 knockdown group can effectively promote the migration (P=0.002) and invasion (**P=0.005) of MDA-MB-468 cells, and the number of cells passing through the underfloor membrane of the chamber is significantly increased. *P< 0.05, **P< 0.01, ***P< 0.001. Supplementary Fig. 2. Wound healing experiment confirmed that BC069792 can effectively inhibit the migration ability of breast cancer cells. Supplementary Fig. 3. Gene differential expression results after breast cancer cells overexpressed BC069792 a The results of principal component analysis showed that the consistency within the two sample groups was good and had difference. b The results of gene difference analysis showed that the BC069792 overexpression group could cause differential expression of 1209 downstream genes. c The differential expression pathway shown in the figure related to the transduction function of synaptic transmission signal. Supplementary Fig. 4. The exprssion of KCNQ4 protein in the knockdown BC069792 group was significantly reduced (*p=0.014). [file 12943_2023_1747_MOESM1_ESM.zip › Supplementary Materials/Supplementary Figure 4/sup. 4.jpg]

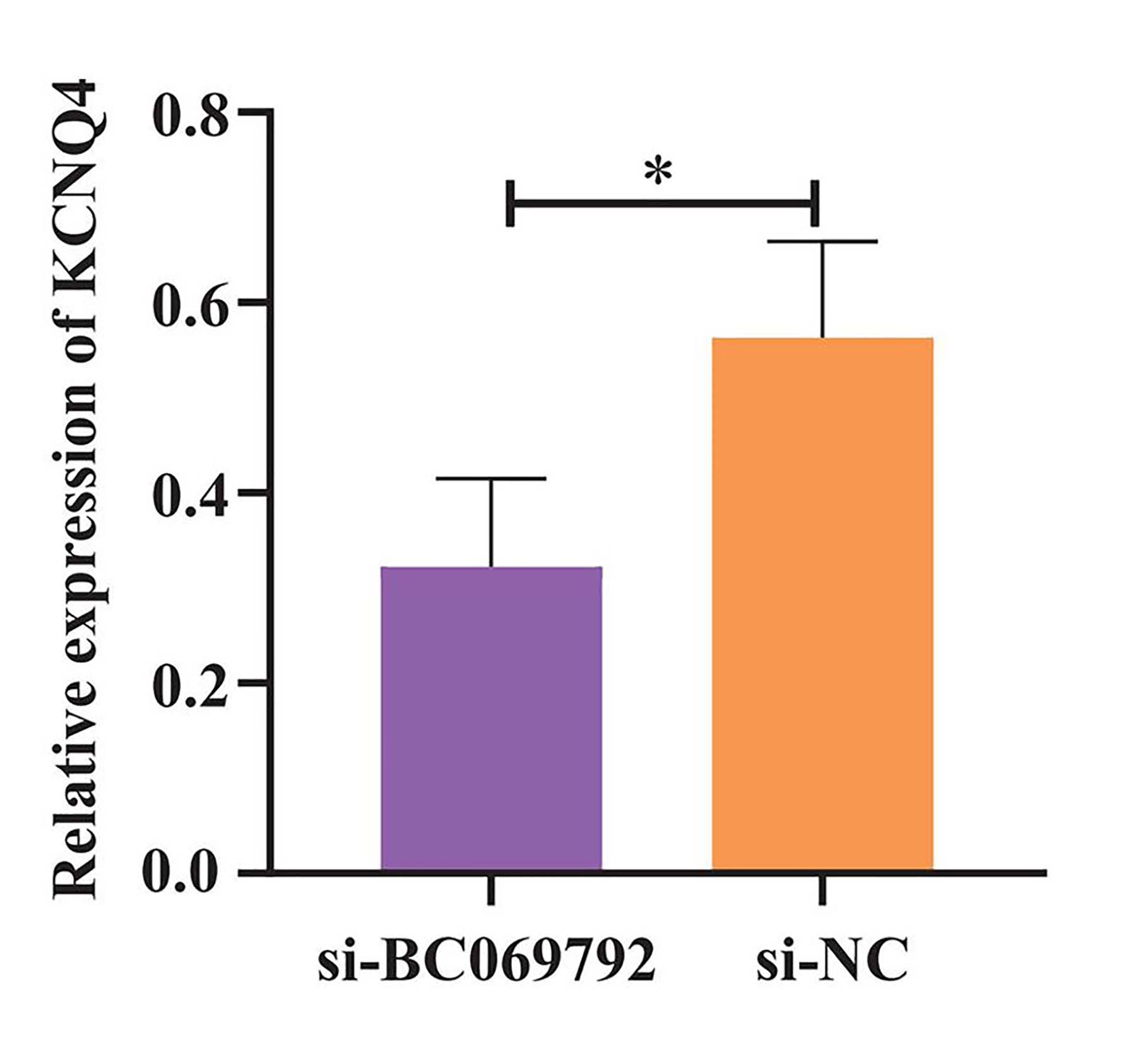

Supplement: Supplementary file 1 — Additional file 1: Supplementary Fig. 1. a In breast cancer cell line and non-tumor cell line MCF-10A, the expression of BC069792 was the highest in non-tumor cell line MCF-10A, while in breast cancer cell line, the expression of BC069792 in MDA-MB-231 and MDA-MB-468 cell lines was the lowest. b CCK-8 experiments showed that si-BC069792 can promote the proliferation of MDA-MB-231 cells (*P=0.43) and MDA-MB-468 (*P=0.026) cells. c The results of EdU experiments showed that si-BC069792 promoted the proliferation ability of breast MDA-MB-231 (*P=0.042) and MDA-MB-468 (*P=0.050) cancer cells. d Compared with the control group, the si-BC069792 knockdown group can effectively promote the migration (P=0.044) and invasion ability (P=0.002) of MDA-MB-231 cells, while the si-BC069792 knockdown group can effectively promote the migration (P=0.002) and invasion (**P=0.005) of MDA-MB-468 cells, and the number of cells passing through the underfloor membrane of the chamber is significantly increased. *P< 0.05, **P< 0.01, ***P< 0.001. Supplementary Fig. 2. Wound healing experiment confirmed that BC069792 can effectively inhibit the migration ability of breast cancer cells. Supplementary Fig. 3. Gene differential expression results after breast cancer cells overexpressed BC069792 a The results of principal component analysis showed that the consistency within the two sample groups was good and had difference. b The results of gene difference analysis showed that the BC069792 overexpression group could cause differential expression of 1209 downstream genes. c The differential expression pathway shown in the figure related to the transduction function of synaptic transmission signal. Supplementary Fig. 4. The exprssion of KCNQ4 protein in the knockdown BC069792 group was significantly reduced (*p=0.014). [file 12943_2023_1747_MOESM1_ESM.zip › Supplementary Materials/Supplementary Figure 4/The expression of KCNQ4 protein in the knockdown BC069792.JPG]
